# Convergent Evolution Has Led to the Loss of Claw Proteins in Snakes and Worm Lizards

**DOI:** 10.1093/gbe/evae274

**Published:** 2024-12-19

**Authors:** Karin Brigit Holthaus, Julia Steinbinder, Attila Placido Sachslehner, Leopold Eckhart

**Affiliations:** Department of Dermatology, Medical University of Vienna, Vienna 1090, Austria; Department of Dermatology, Medical University of Vienna, Vienna 1090, Austria; Department of Dermatology, Medical University of Vienna, Vienna 1090, Austria; Department of Dermatology, Medical University of Vienna, Vienna 1090, Austria

**Keywords:** gene family, gene loss, reptiles, worm lizard, snake

## Abstract

The evolution of cornified skin appendages, such as hair, feathers, and claws, is closely linked to the evolution of proteins that establish the unique mechanical stability of these epithelial structures. We hypothesized that the evolution of the limbless body anatomy of the Florida worm lizard (*Rhineura floridana*) and the concomitant loss of claws had led to the degeneration of genes with claw-associated functions. To test this hypothesis, we investigated the evolution of three gene families implicated in epithelial cell architecture, namely type I keratins, type II keratins, and genes of the epidermal differentiation complex in *R. floridana* in comparison with other squamates. We report that the orthologs of mammalian hair and nail keratins have undergone pseudogenization in *R. floridana*. Likewise, the epidermal differentiation complex genes tentatively named *EDYM1* and *EDCC*s have been lost in *R. floridana*. The aforementioned genes are conserved in various lizards with claws, but not in snakes. Proteomic analysis of the cornified claws of the bearded dragon (*Pogona vitticeps*) confirmed that type I and type II hair keratin homologs, EDYM1 and EDCCs, are protein components of claws in squamates. We conclude that the convergent evolution of a limbless body was associated with the convergent loss of claw keratins and differentiation genes in squamates.

**Significance**

The protein components of cornified skin appendages, such as claws, and the cornified layer of the epidermis, are encoded by three gene families, namely type I and type II keratins and epidermal differentiation complex (EDC) genes. To determine which members of these gene families are associated with functions in claws, we performed comparative genomics of reptiles with claws and limbless reptiles that lack claws. We report that both the Florida worm lizard and snakes have lost homologs of keratins and EDC genes that are expressed in the claws of limbed lizards. These results suggest that worm lizards and snakes have undergone convergent evolution at the morphological and molecular levels.

## Introduction

Cornified skin appendages, such as claws, hair, and feathers, have evolved in land-dwelling vertebrates as important and partly lineage-specific adaptations (Wu et al. 2004; Alibardi et al. 2009; Fleckman et al. 2013; Chen et al. 2015; Akat et al. 2022; Eckhart et al. 2024). They are formed by terminal differentiation of epithelial cells, known as keratinocytes, which accumulate cross-linked proteins. Upon cornification, the keratinocytes undergo programmed cell death but remain interconnected by stable cell junctions (Candi et al. 2005; Harland and Plowman 2018; Matsui 2023). The main protein components of hair and nails are the so-called hair keratins, which are cysteine-rich proteins of the monophyletic subfamilies of type I and type II keratin intermediate filament proteins (Eckhart and Ehrlich 2018; Shim et al. 2022). Orthologs of hair keratins are expressed in the claws of reptiles (Eckhart et al. 2008; Alibardi et al. 2011) and amphibians (Carron et al. 2024), but do not exist in lungfish, suggesting that hair keratin subclades of type I and type II keratins have originated in basal tetrapods (Vandebergh et al. 2013; Carron et al. 2024). In the green anole lizard, some hair keratin homologs are expressed exclusively in claws, whereas others are expressed in both claws and skin (Eckhart et al. 2008), suggesting functional diversification of these keratins. Scales and feathers of sauropsids (reptiles and birds) contain additional keratins (hard acid sauropsid-specific [HAS] keratins and hard basic sauropsid-specific [HBS] keratins) that have convergently evolved a high cysteine content (Ehrlich et al. 2020). Other important protein components of cornified keratinocytes are encoded by genes of the epidermal differentiation complex (EDC) (Candi et al. 2005; Strasser et al. 2014, 2015; Alibardi et al. 2016; Holthaus and Eckhart 2024). Many of these epidermal differentiation proteins are covalently cross-linked by transglutamination under the control of transglutaminases (Candi et al. 2005; Sachslehner et al. 2023), and others have a high abundance of cysteine residues indicative of disulfide bond–dependent cross-linking (Lachner et al. 2019; Ishitsuka and Roop 2020).

Skin appendages such as claws have been lost in several lineages of tetrapods (Gans 1975; Apesteguía and Zaher 2006; Alibardi 2009; Yenmiş and Ayaz 2023). The loss of cornified skin appendages was associated with the degeneration of genes that function specifically in skin appendages (Cohn and Tickle 1999; Emerling 2017; Leal and Cohn 2018). The most prominent examples of pseudogenization and gene loss are provided by hair/claw keratin homologs in whales and dolphins (*Cetacea*, *Mammalia*) (Hecker et al. 2017; Ehrlich et al. 2019), snakes (*Serpentes*, *Sauropsida*) (Dalla Valle et al. 2011; Ehrlich et al. 2020), and caecilians (*Gymnophiona*, *Amphibia*) (Carron et al. 2024). Two types of genes of the EDC, tentatively named *EDYM1* and *EDCC*s, were found to be conserved in diverse reptiles but not in snakes, pointing to roles of these genes in cornified claws (Holthaus et al. 2017). Recently, we have reported that the gene for transglutaminase 9 has degenerated upon the evolutionary loss of the cornified egg tooth in mammals and upon the loss of cornified claws in snakes and worm lizards (Sachslehner et al. 2024).

The Florida worm lizard (*Rhineura floridana*) is the first member of the clade *Amphisbaenia* whose genome sequence has been determined (Rhie et al. 2021). Phylogenetically, *Amphisbaenia* are positioned within the clade *Lacertoidea* (Vidal and Hedges 2005; Streicher and Wiens 2017). The fossorial lifestyle of amphisbaenians has led to several anatomical adaptations such as the degeneration of eyes, modifications of the head, the elongation of the body, and the reduction or loss of limbs and digits (Gans 1975; Kearney and Stuart 2004; Wiens et al. 2006; Foureaux et al. 2010; Allam et al. 2016). The molecular changes underlying the modifications of the skin and its appendages in relation to the fossorial adaptation of squamates are not known.

Here, we performed a comparative genomics study to determine which skin epithelium-associated genes were convergently lost during the evolutionary degeneration of limbs in the Florida worm lizard and in snakes.

## Results

### Homologs of Type I Hair Keratin Genes Are Disrupted by Mutations in the Worm Lizard

To test the hypothesis that evolution of a limbless body has rendered claw-specific genes dispensable and allowed for appearance of disruptive mutations or even gene loss, we investigated three clusters of genes in which candidate claw genes were identified in other squamate reptiles: type I keratin genes, type II keratin genes, and the EDC (Eckhart et al. 2008; Holthaus et al. 2017; Ehrlich et al. 2020; Alibardi 2021). Gene sequences were obtained by downloading gene predictions available in GenBank and by the identification of additional genes and pseudogenes following a published approach (Holthaus et al. 2017; Ehrlich et al. 2020) in the genome sequences of the Florida worm lizard (*R. floridana*) (Rhie et al. 2021) and the green anole lizard (*Anolis carolinensis*) (Alföldi et al. 2011; supplementary table S1, Supplementary Material online).

**Fig. 1.**
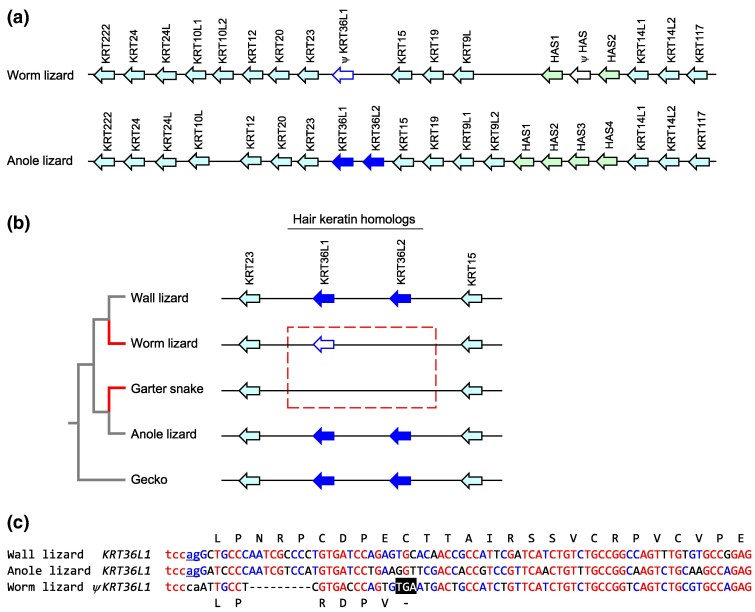
Mutations disrupt hair keratin homologs in the type I keratin gene cluster of the worm lizard. a) The type I keratin gene clusters of the worm lizard and the anole lizard are depicted schematically. Genes are represented by arrows pointing in the direction of transcription. Hair keratin homologs (*KRT36-likes*, *KRT36L*) are shaded dark blue, green indicates sauropsid-specific HAS keratin genes, and other keratin genes are shaded in light blue. Pseudogenes are marked with the symbol ψ and displayed as white arrows. b) Comparison of the *KRT36L* loci between different lepidosaur species reveals convergent loss of *KRT36L* genes in the worm lizard and snakes. In both sister groups and the outgroups, two *KRT36L* genes are present. Phylogenetic relationships of species are shown on the left. c) Nucleotide sequence alignment of a fragment of the highly degenerated *KRT36L1* pseudogene of the worm lizard and homologous segments of intact *KRT36L1* genes of other species. Above and below the nucleotide sequences, the amino acid sequence of the corresponding protein is shown. A premature stop codon is indicated by white fonts on black background. Red and blue letters indicate conservation in at least three and two species, respectively. Red letters indicate sequence identity. Lower case letters belong to introns. The consensus motifs of splice acceptor sites are underlined. Note the loss of the splice site in the worm lizard. The sequence of worm lizard *ψKRT36L1* corresponds to GenBank accession number NC_084490.1, nucleotides 49197581 to 49197865. Keratin names were assigned according to the study by Ehrlich et al. (2020). Species: anole lizard (*A. carolinensis*), garter snake (*T. elegans*), gecko (*G. japonicus*), wall lizard (*P. muralis*), and worm lizard (*R. floridana*).

The arrangement of genes in the type I keratin cluster of the worm lizard is similar to that of the green anole lizard (Fig. 1a; supplementary table S1, Supplementary Material online). However, there is no protein-coding homolog of the anole lizard *KRT36-like* (*KRT36L*) genes, previously referred to as hard acidic (HA) 1 and HA2 keratins (Eckhart et al. 2008), between *KRT23* and *KRT15* in the worm lizard (Fig. 1a). Next, we compared the specific locus of *KRT36L* genes and their flanking genes in a phylogenetically broader group of lepidosaurs (Fig. 1b). Homologs of hair keratins (*KRT36*-like genes) are absent in the worm lizard and in the garter snake (supplementary table S2, Supplementary Material online), which is in agreement with the proposed loss of type I hair keratin genes at the base of *Serpentes* (Dalla Valle et al. 2011; Emerling 2017; Ehrlich et al. 2020). By contrast, two *KRT36*-like genes are present in the wall lizard, the anole lizard, and the gecko (Fig. 1b). Since the gecko is a phylogenetically basal squamate, it can be deduced that the ancestral condition for squamates was the presence of two *KRT36*-like genes. A gene remnant (pseudogene, *ψKRT36L1*) was found at the expected locus of *KRT36L1* in the worm lizard (Fig. 1b). Alignment of *KRT36-like* nucleotide sequence stretches with the sequences of *KRT36L1* genes of the wall lizard, and the anole lizard revealed mutations leading to the loss of a consensus splicing signal, a premature stop codon (Fig. 1c), and other defects in *ψKRT36L1* of the worm lizard.

### Homologs of Type II Hair Keratin Genes Are Disrupted by Mutations in the Worm Lizard

**Fig. 2.**
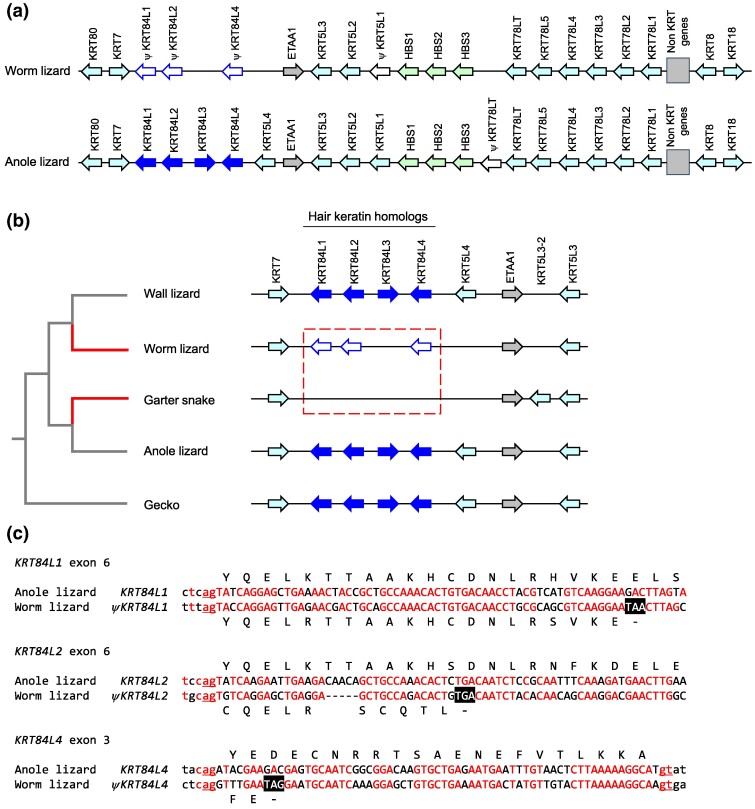
Mutations disrupt hair keratin homologs in the type II keratin gene cluster of the worm lizard. a) The type II keratin gene clusters of the worm lizard and the anole lizard are depicted schematically. Genes are represented by arrows pointing in the direction of transcription. Hair keratin homologs (*KRT84-likes*, *KRT84L*) are shaded in dark blue, green indicates sauropsid-specific HBS keratin genes, and other keratin genes are shaded in light blue. Pseudogenes are marked with the symbol ψ and displayed as white arrows. b) Comparison of the *KRT84L* loci between different lepidosaur species reveals convergent loss of *KRT84L* genes in the worm lizard and snakes. In both the snake and the worm lizard sister groups, four *KRT84L* genes are present as well as in the outgroup (gecko). Phylogenetic relationships of species are shown on the left. c) Alignments of nucleotide sequences of partial *KRT84L* pseudogenes of the worm lizard and homologous segments of intact *KRT84L* genes of the anole lizard. Premature stop codons are highlighted by white fonts on black background. Above and below the nucleotide sequences, the amino acid sequences of the corresponding proteins are shown. Red letters indicate sequence identity. Lower case letters belong to introns. The consensus motifs of splice acceptor and donor sites are underlined. The sequences of worm lizard pseudogenes correspond to GenBank accession number NC_084482.1, nucleotides 21376268 to 21376341 (*ψKRT84L1*), 21360260 to 21360328 (*ψKRT84L2*), 21357117 to 21357190 (*ψKRT84L4*). Keratin names were assigned according to the study by Ehrlich et al. (2020). Species: anole lizard (*A. carolinensis*), garter snake (*T. elegans*), gecko (*G. japonicus*), wall lizard (*P. muralis*), and worm lizard (*R. floridana*).

Like the keratin type I keratin gene cluster, the type II keratin gene cluster of the worm lizard resembles the homologous gene cluster of the anole lizard (Fig. 2a; supplementary tables S3 and S4, Supplementary Material online). Different from previously investigated species of vertebrates, both the worm lizard and the wall lizard contain nonkeratin genes that interrupt the type II keratin cluster (Fig. 2a). The worm lizard lacks orthologs of *KRT5L1* and *KRT5L4* of the anole lizard, and, most importantly, the genes encoding type II hair keratins are nonfunctional in the worm lizard. In contrast to the presence of four protein-coding *KRT84*-like genes in squamates with limbs (wall lizard, anole lizard, and gecko) (Fig. 2b), no *KRT84*-like genes exist in the garter snake and other snakes (Ehrlich et al. 2020), and three *KRT84*-like pseudogenes (*ψKRT84L1*, *ψKRT84L2*, and *ψKRT84L4*) are present in the worm lizard. Notably, these pseudogenes are incomplete remnants of ancestral genes with sequence segments being similar to only few of the original exons. Alignment of the nucleotide sequences showed that premature stop codons in these exon remnants and a frameshift in a degenerated exon of *ψKRT84L2* disrupt the coding sequences (Fig. 2c).

### The EDC of the Worm Lizard Is Largely Syntenic With the EDC of the Green Anole Lizard

In contrast to the availability of reliable predictions for most keratin genes of the worm lizard, only few genes of the EDC were correctly predicted prior to our study. To enable the comparative analysis of the EDC, we applied a combination of tBLASTn searches using EDC proteins of other squamates as queries and de novo gene predictions from translated nucleotides sequences (Strasser et al. 2014) in the EDC regions of the Florida worm lizard (*R. floridana*). In particular, we used sequences of EDC genes of the common wall lizard (*Podarcis muralis*) (Holthaus et al. 2024). Preliminary names were assigned to the numerous newly identified genes of the EDC. Full gene names with brief explanation are listed in supplementary table S5, Supplementary Material online, whereas in the text and figure, abbreviations are used starting with ED (epidermal differentiation) followed by a term indicating either a specific amino acid composition or motif of the protein encoded. The genomic locations of EDC genes identified in the worm lizard are listed in supplementary table S6, Supplementary Material online. Amino acid sequences of EDC proteins are listed in supplementary fig. S1, Supplementary Material online.

**Fig. 3.**
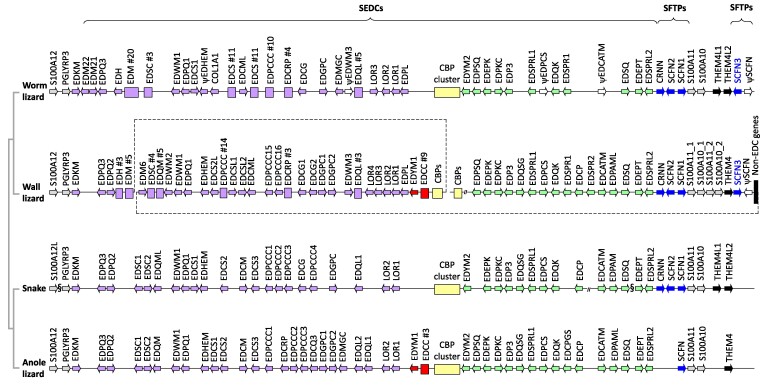
The EDC of the worm lizard in comparison with the EDC of other lepidosaurs. The EDC of the worm lizard (*R. floridana*) is schematically depicted, whereby the genes are aligned to those in the EDC of the wall lizard (*P. muralis*), a snake (*Ophiophagus hannah*), and the anole lizard (*A. carolinensis*). Phylogenetic relationships of species are shown on the left. Single-copy genes are represented by arrows pointing in the direction of transcription, whereas arrays of three or more paralogous genes are shown as boxes, with the number of genes being indicated after the symbol #. Colors highlight the following groups of EDC genes: red, genes missing in both worm lizard and snakes; yellow, corneous beta-proteins; green, genes located between *CBP*s and *SFTP*s; blue, *SFTP*s; and violet, genes located between *PGLYRP3* and *CBP*s. Note that the EDC of the wall lizard has undergone a rearrangement with three segments, here connected by broken lines, being syntenic with segments of the EDC in other lepidosaurs (Holthaus et al. 2024). Non-EDC genes are shown in black. Members of gene families are numbered in each species without inferring 1:1 orthology to genes of the same number in other species. The symbol ψ indicates pseudogenes. The symbol // is used to indicate gaps in the sequence assembly. The symbol § indicates separation of genes on different scaffolds. The schematic depiction is not drawn to scale.

The general organization of the EDC of the worm lizard is similar to that of other lepidosaurs (Strasser et al. 2014; Holthaus et al. 2017, 2020) with the exception of the wall lizard in which EDC genes are rearranged (Fig. 3). S100A genes at the extremes of the EDC, *PGLYRP3*, S100 fused-type protein (SFTP), and single-coding exon EDC (SEDC) genes were identified in the worm lizard like in the EDC of other amniotes. Like other lepidosaurs, the worm lizard has a cluster of corneous beta protein (CBP) or beta-keratin genes in the central region of the EDC with SEDC genes encoding proline-rich proteins on one side and different types of SEDC genes on the other side (Fig. 3). Most of the EDC genes of the wall lizard are conserved in the worm lizard, but there are notable exceptions, namely *EDCATM*, *EDCC*, *EDCP*, *EDHEM*, *EDPAML*, *EDPCS*, *EDPQ2*, *EDQM*, *EDQSG*, *EDWM2*, *EDWM3*, and *EDYM1*. The *EDYM1* and *EDCC* genes are also absent in the python and cobra (Holthaus et al. 2017). As the latter genes represent candidates for an evolutionary association with limbs, we investigated them further.

### EDC Genes Associated With Claws in Lepidosaurs Have Been Lost in the Worm Lizard

**Fig. 4.**
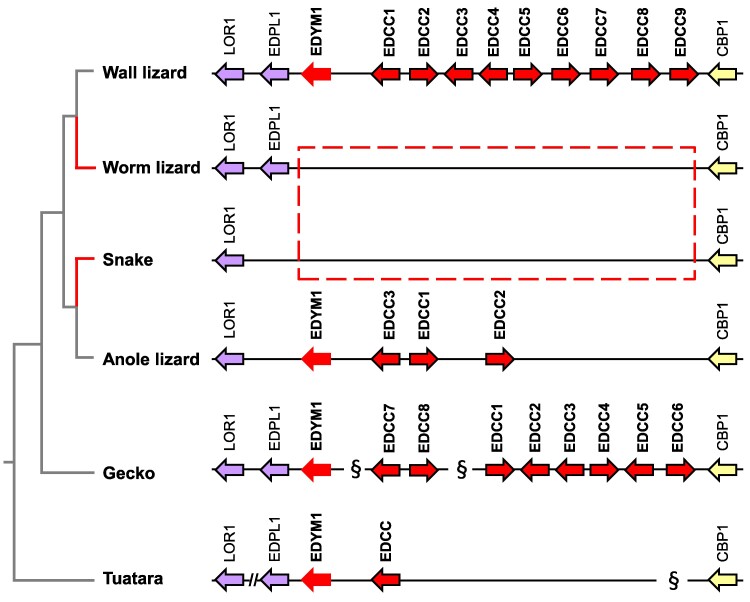
EDC genes associated with claws in lepidosaurs have been lost in the worm lizard. Comparison of the *EDCC* and *EDYM1* gene locus between the worm lizard and other lepidosaurs. In both the limbless worm lizard and snakes, *EDCC* and *EDYM1* genes have been lost while they are present in the wall and anole lizard, gecko, and tuatara. Phylogenetic relationships of species are shown on the left. Genes are represented by arrows pointing in the direction of transcription. The symbol // is used to indicate gaps in the sequence assembly. The symbol § indicates separation of genes on different scaffolds.

*EDYM1* and *EDCC* were previously found in the anole lizard but not in snakes, leading to the hypothesis that they might have a specific function in claws (Holthaus et al. 2017). Here, we show that *EDYM1* and *EDCC* are also absent from the EDC of the worm lizard (Fig. 4). By contrast, *EDYM1* is conserved as a single-copy gene, and three to nine *EDCC* paralogs are present in other lepidosaurs except snakes (Fig. 4).

Having identified *EDYM1* and *EDCC*s as potentially claw-associated genes, we investigated their expression by reverse transcription polymerase chain reaction (RT-PCR) and mass spectrometry–based proteomics of claws of the green anole lizard. RT-PCR showed that *EDYM1*, *EDCC1*, *EDCC2*, and *EDCC3* are expressed in clawed toes (supplementary fig. S2, Supplementary Material online). The proteomic analysis confirmed the expression of EDCC1 and EDCC2 in clawed toes but not in the skin of the back (supplementary tables S7 and S8, Supplementary Material online).

Finally, we performed a proteomic analysis of claws and skin of the bearded dragon (*Pogona vitticeps*), in which claws could be separated from adjacent tissue more efficiently than from the small toes of *A. carolinensis*. This analysis showed that EDYM1- and EDCC-like proteins were present in claws, but not in the skin (Table 1). Likewise, homologs of hair keratins were detected in the claws and not in the skin (Table 1). Among all proteins detected, a type I hair keratin homolog (GenBank gene LOC110074843) and a type II hair keratin homolog (GenBank gene LOC110070014) had the highest Sequest score, which is a measure of confidence of detection (supplementary tables S9 and S10, Supplementary Material online). These data show that orthologs of genes that have been lost or pseudogenized in the worm lizard are expressed in the claws of limbed lizards.

**Table 1:** Proteomic analysis of claws and skin of the bearded dragon (*P. vitticeps*)

Protein type	Protein	Accession (UniProt)	Gene symbol (GenBank)	Description (GenBank)	Expression sites (detection by MS)		Evolutionary fate of ortholog in worm lizard
					Claws	Skin	
Keratins, type I	KRT36L1	A0A6J0T4Z5	LOC110074843	Keratin, type I cuticular Ha6-like	+	−	Loss
	KRT36L2	A0A6J0T2L7	LOC110074851	Keratin, type I cuticular Ha6-like	+	−	Loss
	KRT14L2	A0A6J0T2V3	LOC110074892	Keratin, type I cytoskeletal 14-like	+	−	
	KRT14L1	A0A6J0T2W2	LOC110074898	Keratin, type I cytoskeletal 14-like	+	+	
	KRT15	A0A6J0SXS2	LOC110074850	Keratin, type I cytoskeletal 15-like isoform X1	+	+	
	KRT24L	A0A6J0T561	LOC110074871	Keratin, type I cytoskeletal 15-like isoform X2	−	+	
	KRT24	A0A6J0SY29	LOC110074904	Keratin, type I cytoskeletal 24-like isoform X2	−	+	
	KRT9L1^a^	A0A6J0SXS9	LOC110074853	Keratin, type I cytoskeletal 10-like	−	+	
	HAS4/KRT9LC4	A0A6J0SXW5	LOC110074873	Keratin, type I cytoskeletal 10-like	−	+	
Keratins, type II	KRT84L1	A0A6J0SDW8	LOC110070014	Keratin, type II cuticular Hb5-like	+	−	Loss
	KRT84L2	A0A6J0SEM6	LOC110070016	Keratin, type II cytoskeletal 8-like	+	−	Loss
	KRT84L3	predicted	LOC110070017	Keratin, type II cuticular Hb2-like	+	−	Loss
	KRT84L4	A0A6J0SA49	LOC110070015	Keratin, type II cytoskeletal cochleal-like isoform X1	+	−	
	KRT5L4	A0A6J0SA54	LOC110070018	Keratin, type II cytoskeletal cochleal-like	+	+	
	KRT5L2	A0A6J0VBD4	LOC110090296	Keratin, type II cytoskeletal 5-like isoform X2	+	+	
	KRT78L2	A0A6J0VFV2	LOC110090297	Keratin, type II cytoskeletal cochleal-like	+	+	
	KRT78L5	A0A6J0VFU1	LOC110090289	Keratin, type II cytoskeletal 5	−	+	
	HBS2/KRT78LC2	A0A6J0VBD1	LOC110090293	Keratin, type II cytoskeletal 7-like	−	+	
	KRT78L4	A0A6J0VFV7	LOC110090299	Keratin, type II cytoskeletal 5	−	+	
EDC proteins	EDYM1	n.a.	n.a.	NW_018151906.1:92820-93404 (coding sequence)	+	−	Loss
	EDCCL1	n.a.	n.a.	NW_018151906.1:c70382-70750 (coding sequence)	+	−	Loss
	EDCCL2	n.a.	n.a.	NW_018151906.1:c80185-80565 (coding sequence)	+	−	Loss
	EDCCL4	n.a.	n.a.	NW_018151906.1:84888-85103 (coding sequence)	+	−	Loss
	CBP1	n.a.	n.a.	NW_018151906.1:54057-55130 (coding sequence)	+	−	
	EDGY1	n.a.	n.a.	NW_018151895.1:c48244-48633 (coding sequence)	+	−	
	EDCP	n.a.	n.a.	NW_018151765.1:13812-14501 (coding sequence)	+	−	
Desmosomal proteins	DSP	A0A6J0TYQ4	DSP	Desmoplakin	+	+	
	PKP1	A0A6J0SNS3	PKP1	Plakophilin-1	+	+	
	DSG1	A0A6J0TDZ5	LOC110077646	Desmoglein-1-beta-like	+	+	
	DSC1	A0A6J0UWW8	LOC110087251	Desmocollin-1-like	+	+	
	JUP	A0A6J0T2Q4	JUP	Junction plakoglobin	+	+	
	PKP3	A0A6J0STP6	PKP3	Plakophilin-3	+	+	

Proteins were named according to the nomenclature used previously for other sauropsids (Ehrlich et al. 2020). Orthology is uncertain for some proteins (^a^). Amino acid sequences of EDC proteins were obtained by translation of predicted genes. GenBank accession numbers of coding sequences are provided under “Description.” Detailed information on results of proteomics is provided in supplementary tables S7 and S8, Supplementary Material online. In the column “Evolutionary fate of ortholog in worm lizard,” the loss of proteins is highlighted. Other evolutionary changes are not indicated.

MS, mass spectrometry; n.a., not applicable.

**Fig. 5.**
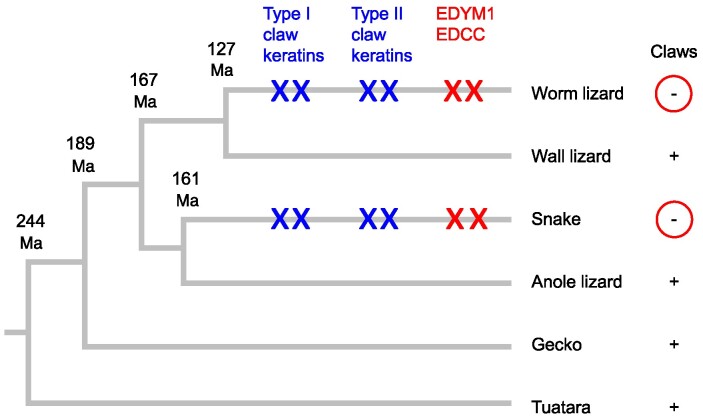
Model of convergent evolution in limbless reptiles: snakes and the worm lizard. The phylogenetic tree shows relationships of lepidosaurs investigated in this study and divergence times (Ma) (Kumar et al. 2022) of the corresponding lineages. In both the worm lizard and snakes, limbs and claws have been lost. Xs on the branches indicate the loss of at least two genes of the following groups: type I claw keratins (orthologs of mammalian hair keratin *KRT36*), type II claw keratins (orthologs of mammalian hair keratin *KRT84*), and EDC genes (*EDYM1* and *EDCC*). Species: worm lizard (*R. floridana*), wall lizard (*P. muralis*), snake (*T. elegans*), anole lizard (*A. carolinensis*), gecko (*G. japonicus*), and tuatara (*Sphenodon punctatus*).

Taken together, the comparative analysis of keratin and EDC genes in the selected panel of squamate species leads to a model for the evolutionary degeneration of cornified claws at the molecular level (Fig. 5). The Florida worm lizard and snakes lack homologs of type I hair/claw keratins (*KRT36-likes*), type II hair/claw keratins (*KRT84-*likes), and specific EDC genes (*EDYM1* and *EDCC*s), which are conserved in other squamates investigated and which are expressed in the claws of at least one lizard, the bearded dragon. This indicates that all the aforementioned genes were present in the last common ancestor of squamates and that they underwent convergent loss or pseudogenization in two lineages that lost limbs and also claws (Fig. 5).

## Discussion

The loss of a phenotypic trait is an intriguing evolutionary process that allows to identify trait-specific genes by comparative genomics and gene expression analyses. The loss of pigments in cave-dwelling animals of diverse taxa (Aardema et al. 2020); the loss of teeth as adaptation to specializations in food uptake in frogs, birds, turtles, baleen whales, anteaters, and others (Meredith et al. 2013, 2014; Paluh et al. 2021); and the loss of limbs in cetaceans, snakes, and caecilians (Cohn and Tickle 1999; Thewissen et al. 2006; Kvon et al. 2016; Leal and Cohn 2016, 2018; Roscito et al. 2018, 2022; Sun et al. 2022; Ovchinnikov et al. 2023) have been studied extensively, leading to important insights into the molecular regulation of traits and dynamics of evolution. Importantly, the independent loss of a particular trait in different lineages is predicted to lead to the parallel loss of those genes that are required for the development and function of this trait, but not for other traits under selective constraints. The present study contributes another example to this concept by showing that parallel loss of claws manifests in loss-of-function mutations in claw-specific genes.

Our results show that specific intermediate filament proteins of the type I and type II keratin families and a subgroup of EDC proteins have been lost in the Florida worm lizard. The keratins affected by pseudogenization are homologs of the so-called hair keratins, which actually evolved as claw keratins (Eckhart et al. 2008; Langbein et al. 2010; Carron et al. 2024) and were co-opted to hair only in mammals. Type I and type II hair/claw keratins originated in amphibians and were demonstrated to be components of cornified claws of clawed frogs (Carron et al. 2024), anole lizards (Eckhart et al. 2008), mice (Jaeger et al. 2019), and humans (Perrin et al. 2004). In the first report on hair keratin homologs in the lizard *A. carolinensis*, two genes (then termed HA1 and HB1) were detected by RT-PCR exclusively in claws, whereas the other homologs of hair keratins (HA2, HB2, and HB3) were expressed in the claws, abdominal skin, and the tongue (Eckhart et al. 2008). In line with these mRNA data, we detected the protein Krt36L2, which is equivalent to HA2 (Eckhart et al. 2008), not only in claws (supplementary table S7, Supplementary Material online) but also in back skin (supplementary table S8, Supplementary Material online). Given the apparently pleiotropic roles of some hair keratin homologs, it cannot be predicted with certainty that the evolutionary loss of claws (and hair in mammals) will make the corresponding genes dispensable and therefore prone to pseudogenization. A disruptive point mutation was detected in a type I hair keratin gene of the limbless slowworm (*Anguis fragilis*) (Dalla Valle et al. 2011). The results of the present study reveal degeneration of all hair keratin orthologs in the Florida worm lizard, indicating that the main ancestral function of these proteins was the molecular architecture of claws, which are absent in this limbless lizard. The identification in the Florida worm lizard of pseudogenes, representing gene fossils, at loci syntenic with orthologous genes of other species, confirms that genes were not missed by our search, but indeed inactivated by mutations. Notably, the genes flanking hair keratin homologs were conserved—with variable distances between them—in the species investigated (supplementary table S11, Supplementary Material online).

The role of EDC proteins in claws has been much less investigated than the role of keratins (Alibardi 2021). Several papers refer to a subgroup of EDC proteins as claw beta-keratins or “claw keratins” (Whitbread et al. 1991). These proteins are CBPs, the corresponding genes are located in the EDC, and their expression was detected in cornified claws of birds (Holthaus et al. 2018). However, “claw keratins” of birds are not specifically expressed in claws (Wu et al. 2015). In squamates, some CBPs are expressed predominantly in claws (Dalla Valle et al. 2010). Moreover, a relatively high number of presumably redundant CBP paralogs is present in all sauropsid species investigated (Holthaus et al. 2018), including snakes (Holthaus et al. 2017), and CBP pseudogenes have been identified in many sauropsids with and without claws (Holthaus et al. 2016, 2017, 2020). Therefore, we screened other EDC genes of the worm lizard for inactivating mutations. We did not find loss-of-function mutations in SFTP genes, which are expressed in epithelial compartments, such as the subunguis of the claw (Mlitz et al. 2014; Alibardi et al. 2015). Possibly, the conservation of SFTPs in worm lizards and snakes is due to additional functions of SFTPs in the periderm during embryonic development (Eckhart et al. 2024). However, we did detect the loss of particular EDC genes in worm lizards, and strikingly, two of these genes, *EDYM1* and *EDCC*, have also been lost in snakes (Holthaus et al. 2017). The expression of *EDYM1* and *EDCC* in the clawed toes of the green anole lizard supports the hypothesis that they play a role in claws; however, the toe samples comprised the claw-forming epithelium and adjacent tissue. Strong evidence for functions of these proteins in claws is provided by the detection of EDYM1 and EDCC homologs in the isolated claws of the bearded dragon. Notably, the avian ortholog of EDYM1 was detected by proteomics in the claws, but also in the beak and scales of the chicken (Strasser et al. 2014). *EDYM1* and *EDCC* are not conserved in the python (Holthaus et al. 2017), although this snake develops claw-like structures on vestigial hind limbs, known as pelvic spurs (Gillingham and Chambers 1982). The molecular composition of the epithelium on pelvic spurs and its evolutionary relationship to the cornified epithelium of claws should be investigated in future studies.

The EDC proteins convergently lost in the worm lizard and in snakes do not feature particular amino acid sequences that would suggest specific functions. More cysteine-rich proteins (tentatively named EDCRPs and EDPCCCs), which are assumed to be preferred substrates of disulfide bond–mediated cross-linking, are conserved in both worm lizard and snakes. This suggests that cysteine-dependent protein cross-linking of EDC proteins is not limited to claws. Although the precise contribution of EDYM1 and EDCCs to the cornification of claws remains to be determined, the apparent association of these EDC-encoded proteins with the evolution of claws establishes a predominant role of these EDC proteins in claw formation of squamates. Previously, EDC-encoded CBPs were identified as components of lizard claws (Dalla Valle et al. 2010), and mRNAs of other EDC genes were detected in the toes of *A. carolinensis* (Strasser et al. 2014).

In summary, the results of this study suggest convergent evolution toward loss of claw proteins in limbless squamates. The convergent loss of specific keratins and EDC proteins upon loss of claws supports the concept that the ancestral and predominant function of these proteins was to build cornified claws.

## Materials and Methods

### Comparative Analysis of Keratin Type I and Type II Gene Loci

The complete keratin gene clusters were analyzed in the genomes of the Florida worm lizard (*R. floridana*) and the green anole lizard. For the analysis of keratin type I and type II gene clusters, gene predictions of the Florida worm lizard in GenBank (chromosome accession numbers NC_084490.1 and NC_084482.1, respectively) (Rhie et al. 2021) were used and compared with keratin sequences of the green anole lizard (*A. carolinensis*) (Alföldi et al. 2011; Ehrlich et al. 2020). The sequences of type I and type II keratins (Ehrlich et al. 2020) were corrected, where necessary, based on the current genome sequence version (GenBank accession numbers NC_085846.1 and NC_085842.1, respectively) (supplementary fig. S3, Supplementary Material online). Basic Local Alignment Search Tool (Altschul et al. 1990) searches using keratin sequences of other species were performed to identify the gene segments not predicted in GenBank and pseudogenes. GenBank accession numbers of type I and type II keratins are shown in supplementary tables S1 and S3, Supplementary Material online, respectively.

For comparison of homologous loci, in addition to the above-mentioned species, we investigated the common wall lizard (*P. muralis*) (Andrade et al. 2019), the western terrestrial garter snake (*Thamnophis elegans*) (Vertebrate Genomes Project 2019), and the Japanese gecko (*Gekko japonicus*) (Liu et al. 2015). Accession numbers of genes at the *KRT36L* and *KRT84L* loci are listed in supplementary tables S2 and S4, Supplementary Material online. Sequence alignments were performed with MUSCLE and MultAlin (Corpet 1988).

### EDC Gene Identification in Genome Sequences

A multiple-step approach was used to identify the sequences of EDC genes in the Florida worm lizard (*R. floridana*) (Rhie et al. 2021). EDC genes predicted in the GenBank were compared with EDC genes of other lepidosaurs (Strasser et al. 2014; Holthaus et al. 2017, 2020, 2024) with regard to exon–intron structure and sequence conservation. Genes conforming to the consensus structures of SEDC and SFTPs were used for further studies. Additional EDC genes were identified by tBLASTn searches and de novo prediction of coding sequences according to a published approach (Strasser et al. 2014). The filter for low-complexity regions was deactivated to avoid erroneous exclusion of typical EDC genes containing segments of low sequence complexity. De novo-predicted EDC genes were used as queries to identify similar proteins in the same and in other species. The EDC gene predictions were validated by RNA-seq evidence, if available in the NCBI GenBank browser for “genomic regions, transcripts, and products” (last accessed on 2024 June 5). The loci of *EDYM1*, *EDCC*s, and flanking genes were compared using previously reported EDC gene data (Strasser et al. 2014; Holthaus et al. 2017, 2020, 2024) and new gene predictions in the worm lizard.

### RT-PCR Analysis of Putative Claw-Associated EDC mRNAs of *A. carolinensis*

RNA was prepared from tissues of *A. carolinensis* and reverse-transcribed into cDNA as described previously (Strasser et al. 2014). The following cDNAs of *A. carolinensis* were amplified with intron-spanning primers as indicated: *EDYM1*, 5′-ATCCGCTCGAGAAGGCTTC-3′ and 5′-TGAGGGCTTCATCATACCATACT-3′, *EDCC1*, 5′-GTCTCCAAACGTTGCATTCCACG-3′ and 5′-CAGGGGTGACGTGGATATCT-3′, *EDCC2*, 5′-ACTGTTTCTTCTCCAGACGTCT-3′ and 5′-GTGCAACCATGATCTCGGAC-3′, *EDCC3*, 5′-CCGTTGATGTCCATCTCCATCC-3′ and 5′-ATTCTGAAGGGCTGGTTGGT-3′, *EDQM*, 5′-ACCTTGCTTCAATTTCCAGAGA-3′ and 5′-CTGATCCTGTGACGGCTTTG-3′, *EDCM*, 5′-CGAACTTCCTACACTTGAGCA-3′ and 5′-TGGCAAGATGAGCACAAAAG-3′, and *EEF1A1*, 5′-TTGCCACACTGCCCATATTG-3′ and 5′-CGCTTTCTTGTCAACTGCCT-3′. The PCR products were analyzed by agarose gel electrophoresis in comparison with DNA length marker VI (Sigma Aldrich). The identity of the bands was confirmed by Sanger sequencing.

### Proteomic Analysis of Claws and Skin of Anole Lizard and Bearded Dragon

Claw-bearing toes and back skin of *A. carolinensis* (Strasser et al. 2014) and claws and back skin of *P. vitticeps* were dissected and incubated in lysis buffer containing 30 mM Tris, 7 M urea (VWR), 2 M thiourea (Sigma Aldrich), 4% CHAPSO (Pierce), and 0.2 M dithiothreitol (DTT, Roth) at 70 °C for 3 h (Holthaus et al. 2024). Subsequently, the samples were homogenized in Precellys tubes prefilled with CK14 ceramic beads in a Precellys (VWR) homogenizer. Specimens were centrifuged at 4 °C and 18,000 × *g* for 15 min to remove insoluble debris. The samples were afterwards reduced (0.5 M DTT, Roth), alkylated with iodoacetamide (1 M, Sigma), and bound to sp3 beads (Preomics) at a 10:1 ratio of beads:protein. Specimens were supplemented with 50% acetonitrile solution. After a wash with 80% ethanol, the samples were digested with trypsin/LysC and incubated overnight at 37 °C. Then, the samples were acidified with 30% trifluoroacetic acid (TFA) and washed with acetonitrile and 0.1% TFA. Solid phase extraction was done with 90% acetonitrile and 0.4% formic acid. An Orbitrap Exploris 480 MS with FAIMS (Thermo Fisher) connected to an Ultimate 3000 (Thermo Fisher) nano-high-performance liquid chromatography system was used for the proteomic analysis as described previously (Holthaus et al. 2024). The protein reference databases for *A. carolinensis* (taxonomy ID: 28377, reference proteome: UP000001646) and *P. vitticeps* (taxonomy ID: 103695, reference proteome: UP000504619) were downloaded from UniProt. Peptides were identified with Proteome Discoverer Software (Version: 2.4.1.15).

## Supplementary Material

evae274_Supplementary_Data

## Data Availability

Proteome data were deposited in the PRIDE database under the accession number PXD054063. Other data are incorporated into the article and its online Supplementary material.
