# Supplementary material for: Convergent Evolution Has Led to the Loss of Claw Proteins in Snakes and Worm Lizards
Source: Genome Biol Evol. 2024 Dec 19;17(1):evae274. doi: 10.1093/gbe/evae274 (PMC11704414; doi:10.1093/gbe/evae274)
Supplement: evae274_Supplementary_Data [file evae274_supplementary_data.pdf]

Supplementary Material: Supplementary Figures and Tables

Convergent evolution has led to the loss of claw proteins in snakes and worm lizards

Karin Brigit Holthaus, Julia Steinbinder, Attila Placido Sachslehner, Leopold Eckhart

Content

Supplementary Figures S1-S3

Supplementary Tables S1-S11

A

>Rf_CBP1
MSSSGVKCVTTPCVASCPDAKVVIHPPPLVLTLPGLSLTTTPNQCLVETKTSCLTNGSEENKGGCSTAVVTKSTGLRALSGGDTPCCTTTCPDSQVVIQP
PPVCITIPGAVLSSYPNECLIETSNPCVPSGSQPRAIARSTSSSDCCLTPHRMSRSTSVPCGLNSTPVCMTQGPSSKVVIHPPPIEVTIPGPILEIAAEE
CAVEVYNPCTTAGALTSGEEKCAITSGEEGEVKALTARALSCTTVCGVLGASSSCISQGPEMKIVIQPPPIEVDLPGPILQVFPEACKVETLNPCAPETQ
ALCSSSEATTSTDLATKNTSLYAIKRPLPDIRRPARPWAEMYSRSLTPRAIIAQPSRFSKYRRSFSTTSCQSSY

>Rf_CBP_last
MSCQTPICSIPCSSPSIGLGSGGFGSGGGYGGGYGGGYGGGYGGGYGGGYGGGISGGAVSSSSLGIIPGASVGCISQVPPSEVVVQPPPFVLTIPGPILS
ASCDPVYVGGYSPCASGGYGSGSSGYGYGSSGYGYGSSGSGYGCGGGSSGGYIGGGYKGGNRGHGPSSKCYPC

>Rf_EDCG
MSCSCCSNCGCCGRSSTRIIYCSMPSQPCCCPCYTPRQSCCCSPCCSSTQSYYAPTKATGCAQMQQNYMPSKKCC

>Rf_EDCML
MSCCSQCGCSLCCCKQCCCSPCSCSPSSCNSCCGGQSRCCGSCCSDSKCCYPKQCKC

>Rf_EDCRP1
MDYSKCSQCGCSPCICKTPSCPECGCSLCICKAPLCAQCGCSPCICKTPRCPECGYSPCIYKTPHCPQCGYSPCICKTPRCPQCGCSPCICKTPCCPECG
CSPCICKTPCCPQCGCSPCICKIPRCPQCGCSPCICKTPCCPECGCSPCICKTPCCPQCGCSPCICKIPRCPQCGCSPCICKTPSCPQCGCSPCICKTPS
CPRCGCSPSICKTPRCPQCGCSPCICKTPSCPQCGCSPCICKSPRCPQCGCSPSICKTPSCPQCGCSPSICKTSRCPQCGCSPCICKTPSCPQCGCSPSI
GKTPRCPQCGCSPCICKTPRCPQCGYSPCICKTPRCPQCGYSPCICKTPRCPQCGCSPSICKTPRCPQCGCSPCICKTPHCPQCGCSPCICKTPRCPQCG
CCPSICKTPRCPQCGCSPCICKTPRCPHCGCSPCICKTPKCPQCGCSPCICKTPRCPQCGCSPCICKTPPCPQCGCSPCICKIPRCPQCGCSPCICKTPR
CPQCGCSPCICKTPRCPQCGCSPCICKSPRCAQCGCSPCICKTPRCPQCGCSPCICKTPRCPQCGCSPCICKTPRCPQCGCAPCI
CKTPRCPQCGCSPCICKTPRYPQCGCTPCIYKTPRCPQCGCSPCICKTPSCPQCGCSPCICKTPCCPQCGCSPCICKPPKCPQCGCSPCICKTPPCPQCG
CSPCICKTPRCPQCGCSPCICKTPRCPQCGCSPCICKTPRCPQCGCSPCICKSPHCAQCGCSPCICKTPRCPQCGCSPCICKTPCCPQCGCSPCICKPPK
CPQCGCSPCICKTPPCPQCGCSPCTCKTPRCPHCGCSPCICKAHRCPQCGCSPCICKTPRCPQCGCSPCICKTPRCPQCGCSPCICKTPCCPQCGCSPCI
CKTPRCAQCGCSPCICKTPRCPQCGCSPCICKTPRCPQCGCSPCICKTPSCPQCGCSPCICKTPSCPQCGCSPCICKTPSCPQCGCSPCICKTPRCPVAA
PLASVRFRTVLTVASSCESARLPGVPSVAALLAFAKLPTVPSVAAVLASASLPRCSKCGCSPCCCHLLKF

>Rf_EDCRP2
MTCCGSKTVCCCNTPSCPKCGCYPCCCKPPHCPECGCSPCICKPPCCPECGCSPCICKTPRCPECGCSPCICKPPCCPECGCSPCICKTPRCPECGCSPC
ICKPPCCPECGCSPCICKPPCCPECGCSPCISKAPRCPECGCSPCICKPPRCPECGCSPCICKTPCCPECGCSPCCCKPPRWSKCRWSPSCCKPPTCPRS
GCSPCICKTPCCPECGCSPCICKPPSCPKCGCSPCICKPPRCPECGCSPCICKTPRCPECGCSPCICKTPCCPQCGCSPCICKPPRCPECGCSPCICKPP
SCPKCGCSPCICKPPRCPECGCSPCICKPPHCPECGCSPCICKTPRCPECGCSPCICKTPCCPQCGCSPCICKPPRCPECGCSPCICKPPSCPKCGCSPC
ICKPPRCHECGCSPCICKPPSCPKCGCSPCICKPPHCPECGCSPCICKTPCCPECGCSPCICKPPRCPECGCFPCICKTPCCPECGCSPCICKTPCCPKC
GCSPCICKTPRCPQCGCSPCICNTPRCLQCGCSPCICKPPSCPKCGCSPCICKPPRCPECGCSPCICKTPCCPECGCSPCICKTPCCPKCGCSPCICKTP
RCPQCGCSPCICNTPCCLQCGCSPCICKPPRCPECGCSPCICKTPHCSKCGCSSCTCKSPWCPKCGCSPCCCKPPRCPKCGCFPCICKALWCPKCGCSPC
CCGQSCCGSKSSSACSCYQSRF

>Rf_EDCRP3
MASCGSKTPCAAQISSYPKCGCTPCICKAPSCPKCGCSPCICKSPHCPKCGCSPCICKTPSCPKSGCSLCICKAPSCPKCGCSPCICKTPHCPKCGCSPC
ICKTPSCPKCGCSPCICKAPSCPKCGCSPCICTAPRCPKCGCSLCCCKTPSCPKCGCSPCICKPPSCPKCGCSPCICKTPSCPKCGCSPCICKAPSCPKC
GCSPCICKAPSCPKCGCSPCICKAPSCPTCGCSPCICKAPSCPKCGCSPCICKAPSCPKCGCSPCICKAPSCPTCGCSPCICKAPSCPKCGCSPCICKAP
TCPKCGCSPCICKPPSCPKCGCSPCICKAPRCPKCGCSPCICKTPSCPKCGCSPCICKAPSCPKCGCSPCICKAPSYPKCGCSPCICKAPSCPKCGCSPC
ICKAPRCPKCGCSPCICKAPSCPKCGCSPCSCNPPSCPKCGCSPCCCKPPCCPKCGCSPCICKAPSCPKCSCSPCICKAPSCPKCGCSPCICKAPSCPKC
GCSPCICKAPRCPKCGCSPCCCKPPSCPKCGCSPCICKAPCCPKCGCSPCICKAPCCPKCGCSPCICKAPCCPKCGCSPCCCGECCCYTPCYCYQSCCCP
YCCHFCTPYRANQCVCSPCFFGWSCCGSQPCCSCSCSWYQSKW

>Rf_EDCRP4
MACCGSKTVCSCNPPSCPKCGCSSCICKAPSCPECGCTPCSCKPPCCPKCGCSPCICKPPSCPRCSCTPCSCKPPCCPKCGCSPCICKPPSCPKCGCSPC
ICKPPSCPKCGCSPCICKPPCCPKCGCTPCICKPPSCPKCGCSPCICKPPSCPKCGCSPCICKPPSCPKCGCSPCICKPPSCPKCGCSPCICKPPCCPKC
GCSPCICKPPCCPKCGCSPCICKPPCCPKCGCSPCICKAPSCPKCGCSPCICKPPCCPKCGCSPCICKPPCCPKCGCSPCICKAPSCPKCGCSPCICKAP
SCPKCGCSPCICKPPSCPKCGCSPCICKPPCCPECGCSPCICKPPCCPKCGCSPCICKAPSCPKCGCSPCICKAPSCPKCGCSPCICKPPCCPKCGCSPC
ICKPPCCPECGCSPCICKPPCCPKCGCSPCICKPPCCPKCGCSPCICKPPCCPECGCSPCICKPPCCPKCGCSPCICKPPCCPECGCSPCICKPPCCPKC
GCSPCICKAPSCPNCGCSPCICKAPSCPKCGCSPCICKAPCCPKCGCSPCCCKPPCCPKCGCSPCICKAPCCPKCGCSPCICKPPSCPKCGCSPWCCKPP
SCPKCGCSPCCCKPPSCPKCGCSPCCCKPPSCPKCGCSPCICKPPSCPKCGCSPCICKPPCCPKCGCSPCICKPPCCPKCGCSPCCCKPPSCPRCSCTPC
SCKPSSCPKCGCSPCICKPPSCPKCGCSPCICKPPCCPKCGCSHCCCKPPSCPRCSCTPCSCKPSSCPKCGCSPCICKPPSCPKCGCYPCICKPPCCPKC
GCSHCCCKPPYCPKCGCSHCCCKPPYCPKCGCSHCCCKPPYCPKCGCSHCCCKPPSCPKCGCSPCICKPPCCPKCGCSHCCCKPPYCPKCGCSHCCCKPP
CCPKCGCSHCCCKPPCCPKCGCSHCCCKPSCCPKCGCSPCCCKPPYCPKCGCSHCCCKPPCCPKCGCSPCSCGQSFCGSKPYCSSTCSFYQSKS

>Rf_EDCS1
MGSECCTCCGGNQSNQQFICVPTQSYYSSGSSCCGSGCGSSCGSCCGSCCGCCGSSGQSCKVIYVRPMQYCCCMPVQYSQNCCMPKNQCC

>Rf_EDCS2L_1
MGSGCCSYADRGPNVMCIPCGGGGGGNSCCSPCYSSPMSYGACQCYSCGGCGGYSGGGCCSGGCNGGGCNGGCSIYMVSNGRRSSCCGSC

>Rf_EDCS2L_2
MGAGCCSSADRGPNVICVPCGGGGGGGCNSCCSPMSYSSPMSYGACQCYSSGGCGCYSGGCNGGGCNGGCSIYMVSNGRRSSCCGGC

>Rf_EDCS2L_3
MGNCNACGGYSDEGPDVICIPCGGGNNGCNSCCSPMTYSSSMSYGGCQCYGCGGCGGCNGGGCNGGCSVYVISNGRRSSCCGM

>Rf_EDCS2L_4
MGACNGCCGYSDEGPNIICIPCGGGNNGCNSCCSPMTYSSPMSYGGCQCYSCGGCGGCNGGGCSGGCSIYVISNRRRSCCGCM

>Rf_EDCS2L_5
MGNCNACGGYSDEGPDVICIPCGGGNNGCNSCCSPMTYSSSMSYGGCQCYGCGGCGGCNGGGCNGGCSVYVISNGRRSSCCGM

>Rf_EDCS3L_1
MTCCSTCGCSPSCCGGCSPSGCSQCGGSSCGCSQCGCSPSCCGGCSPSGCGQSSCSPCGCSQCGCSPSGCGQSSCSPCGCSQCGCSPSGCSQCGCSPSGC
GQSSCSPCGCSQCGCCPCGCSQCGCCPCGCGQSSCSPCGCGQSSCSPSGCSQCGCSPSGCGQSSCSPCGCSQCGCSPCGCGQSSCSPCGCGQSSCSPCGC
GQSSCSPCGCSQCGCSPSGCGQSSCSPCGCSQCGCSPSGCSQCGSSSCGCSNGGCSSCGCSQCGCSSCGCSNGGCSPCGCSQCGCSPSGCSQCGCSPCGC
GQSYCSPCCGSSCGCGSKGYYPQQSMC

>Rf_EDCS3L_2
MTCCSTCGCSPCVCGQSSCSSCGPSKGGCSSKGGCSSKGGCSSKGGCSSKGGCSSKGGCSSCGCGQSSCSSCGSSKGGCSSKGGCSSCGCGQSSCSSCGS
SKGGCSSKGGCSSCGCGQSSCSSCASSKGGCSSKGGCSSCGCGQSSCSSCGSSKGGCSSKGGCSSCGCGQSSCSSCGSSKGGCSSKGGCSSCGCGQSSCS
SCGPSKGGCSSKGGCSSCGCGQSSCSPCGCSQCDCSPCSSGQSSCSSCGCSQCGCSPCGCGQSSCSPCGCGQSSCSPSCGSSCCGSSCGSGSKGCYPHQS
KC

>Rf_EDCS3L_3
MTCCSTCGSSPSCCGGCSPSGCSQCGGSSCGCSQCGCSPCGCSQCGCSPSGCGQSFCSPCGCSQCGCSQCGCSQCGCSQCGCSPCGCSQCGCSPCGCSQC
GCSPSGCSQCGCSPSGCGQSSCSPCGCSQCGCSPCGCGQSSCSPCGCGQSFCSPCGCSQCGCSPCGCSPCGCSPCGCSQCGSSSCGCGQSSCPPSGCSQC
GCSPCGCGQSSCSPCCGSSCCGSSCGCGSKGCYPQQSMC

>Rf_EDCS3L_4
MTCCSTCGCSPCVCGQSSCSSGGPSKGGCSSKGGCSSKGGCSSCGCGQSSCSSCGSSKGGCSSCGCGQSSCSSCGSSKGGCSSKGGSSSCGCGQSSCSSC
GSSKGGCSSKGGCSSCGCGQSSCSSCGCGQSSCSSCGCGQSSCSSCGSSKGGCSSKGGCSPCGCGQSSCSSCGSSKGGCSSKGGCSPCGCGQSSCSSCGS
SKGDCSSKGGCSSCGCGQSSCSSCGSSKGGCSSKGGCSSCGCGQSSCSSCGSSKGGCSSKGGCSSKGGCSSCGCGQSSCSSCGPSKGGCSSKGGCSSCGC
GQSSCSPCGCSQCDCSPCSSGQSSCSSCGCSQCGCSPCGCGQSSCSPCGCGQSSCSPSCGSSCCGSSCGSGSKGCYPHQSKC

>Rf_EDCS3L_5
MTCCSTCGCSPSCCGGCSPSGCSQCGGSSCGCSQCGCSPCGCSQCGCSPCGCSQCGCSPCGCSQCGCSPCGCSQCGCSPSGCGQSSCSPCGCSQCGCSPC
GCGQSSCSPCGCSQCGCSPCGCSPCGCSPCGCSQCGSSSCGCGQSSCSPSGCSQCGCSPCGCGQSSCSPCGCGQSFCSPCGCSQCGCSPCGCSPCGCSPC
GCSQCGSSSCGCGQSSCSPSGCSQCGCSPCGCGQSSCSPCCGSSCCGSSCGCGSKGCYPQQSMC

>Rf_EDCS3L_6
MTCCSTCGCSPCVCGQSSCSSCGPSKGGCSSKGGCSSKGGCSSKGGCSSKGGCSSCGCGQSSCSSCGSSKGGCSSKGGCSSCGCGQSSCSSCGSSKGGCS
SKGGCSSCGCGQSSCSSCASSKGGCSSKGGCSSCGCGQSSCSSCGSSKGGCSSKGGCSSCGCGQSSCSSCGSSKGGCSSKGGCSSCGCGQSSCSSCGPSK
GGCSSKGGCSSCGCGQSSCSPCGCSQCDCSPCSSGQSSCSSCGCSQCGCSPCGCGQSSCSPCGCGQSSCSPCGCGQSSCSPSCGSSCCGSSCGSGSKGCY
PHQSKC

>Rf_EDCS3L_7
MTCCSQCGCSPSGCSQCGCSPSGCGQSSCSPCGCSQCCCSQCGYSPCGCSQCGCSPSGCSQCGCSPSGCSQCGCSPSGCSQCGCSPCGCGQSSCSPCGCG
QSFCSPCGCSQCGCSPCGCSPCGCSPCGCSQCGSSSCGCGQSSCSPSGCSQCGCSPCGCGQSSCSPCCGSSCCGSSCGCGSKGCYPQQSMC

>Rf_EDCS3L_8
MSCCSQCGCSQCGCSQCGCSQCGCSQCGCSQCGCSKGGCSKGGCSSCGCSKGGCSKGGCGCSKGGCGCSKGGCGCSKGGCSSCGCGCSKGGCSKGGCGCS
KGGCGCSKGGCGCSKGGCSSCGCGCSKGGCSKGGCGCSKGGCSSCGCSKGGCGCGCSKGGCGKGGCSQCGCGQSACSPCCGSSCCGCSCGCGSKGCCPQQ
SKC

>Rf_EDCS3_1
MGCCGGYSDRGPKVICIPCGGGNNGCNSSCSPSCCCAPCCGCAPCCGCAPCCAPCCGCAPCCGCAPCCGCAPCCGCAPCCGCAPCCCCAPCCCCAPCCGC
SNGGGSNGGGSICIISNGRRSSCCGM

>Rf_EDCS3_2
MGCCGGYSNRGPKVICIPCNGGDNGCNSSCPPCCCCAPCCGCAPCCGCAPCCGCAPCCGCAPCCGCAPCCGCAPCCCCCPCCCCVPCCGCSNGGSCNGGG
SSGGCSICIISNGKRSSCCGM

>Rf_EDCS3_3
MGCCGGYSDRGPKVICIPCGGGDNGCNSSCSPCCCCAPCCGCVPCCGCAPYCGCAPCCGCAPCCGCAPCCGCAPCCCCAPCCCCAPCCGCSNGGGSSGGC
SLYLISNGRRSSCCGM

>Rf_EDCS4_1
MGCNDCCSSSCGQSSCSPCCGQSSCKPSCGQSSCSSCCGQSTCSPCCGQSSCSPCCGQSSCSPCCGQSSCKPSCGQSSCSPCCGQSTCSPCCGQSTCSPC
CGQSTCSPCCGQSTCSPCCGQSSCKPCCGQSTCSPCCGQSTCSPCCGQSSCKPCCGQSSCSSCCGQSSSPCCGQSLCAPCCPAAISCCQTKIDCGAKK

>Rf_EDCS4_2
MGCNDCCSSSCGQSTCSPCCGQSTCSPCCGQSTCSPCCGQSTCSPCCGQSTCSPCCGQSSCSSCCGQSTCSPCCGQSTCSPCCGQSSCSPSCGQSSCSPC
CGQSSCSSCCGQSSSPSCGQSLCAPCCPAAISCCQTKIDCGAKKC

>Rf_EDCS4_3
MGCNDCCSSSCGQSSCSPCCGQSSCKPSCGQSSCSSCCGQSTCSPCCGQSTCSPCCGQSSCSPCCGQSTCSPCCGQSSCKPSCGQSSCSPCCGQSSCSSC
CGQSTCSPCCGQSTCSPCCGQSSCSPSCGQSSCSPCCGQSSCSSCCGQSSSPSCGQSLCAPCCPAAISCCQTKIDCGAKKC

>Rf_EDCS5_1
MGCNDCCSSSCMPSCCSPCCSQSCSPCCSQFCCGSSKSCCAPCCPLVKSCCTSQSKCCSTMKCYGGFWSTSVSRQKPPAGVTSLTTEQFYLQSHAAELNA
EGFEPPSFTQLFDSLFPFVPPPTQKSLLDSGGGKPLSLCI

>Rf_EDCS5_2
MGCNDCCSSPCMPSCCSPCCSQSCSPCCSQSCSPCCSQSCCGSSKSCCAPCCPLVKSCCTSQSKCCSTMKCYGGFWSTSVSRQKLPAGVTSLTTEQFYLQ
SRAAELNAEGFEPPSFTQLFDSLFPFVPPPTQKSLLDSGGGKPLSLCI

>Rf_EDCS5_3
MGCNDCCSSPCMPSCCSPCCSQSCSPCCSQSCSPCCSQSCCGSSKSCCAPCCPLVKSCCTSQSKCCSTMKCYGGFWSTSVSRQKPPAGVTSLTTEQFYLQ
SRAAELNAEGFEPPSFTQLFDSLFPFVPPPTQKSLLDSGGGKPLSLCI

>Rf_EDEPK
MSVEQSHQQQKQSSVLPPALSKVTSEPISEPLTLHGPDLEPEHQPPATKDPENLLRRDKEHHERNKPPSQPPTSDAEVEDKLESKRGPPSGEEEERPSQA
EQQHKEGLDLPRALSQEFLKTGHPPPPKEAEGHLPTQPPPLVEQHKHQHQQKKQPPQWPPK

>Rf_EDEPT
MAYQCRQPCLPPPTGAPQCPTEAARAPCGEVSSAPCGDPLSNMYQSTQPCLPPPICMPPCPTESLTACAAACSAGEQPCPTPCMEVSSAPCGDPLSNMYQ
STQPCLPPPICMPPCPTESLTACAAACSAGEQPCPTPCMEVSSAPCGDPPSNMYQSTQPCLPPPICMPPCPTESLTTCAPACSASEQPCPLPCMEASSAP
CGSSTLLADAGPCAPPCVTLCSSSSSSECLGASSAPSREAVGCYVMPGSVVCLNPSGHVSVKPGPPSEGEACCDSVFMQPYTPHFS

>Rf_EDETM
MTFSGSLCGYNTSCCVSLPPTTLMLQPPPFAAETKLVDLQIVTPKPPPCEIPECDFHPCSLPPLCCRMPPEPPMVFATFSTCLHEL

>Rf_EDM1
MARVMDSLPFDNLNHPLDESLSVFDSYLKYPGSYPPVQRPSWQCPRGYVRRWWGPFNIPGCGACYIPICCRPW

>Rf_EDM2
MAQVMDSLPLDELHHPEDESLSTFDSFYQLPGLRYPRGYVLRWWGPFNIRGCGYCYIPVCCRRRSYP

>Rf_EDM3
MAQVMDSLPLDELHHPEDDDLSAFDSFRQYPGYCPPVQPVGWRRPYGYVQRWWGPFYIPGCGSCYIPVYCRRRRYP

>Rf_EDM4
MARGMEGIQLDDLYHQEDDIFPPFDSFRQYPGYYPPVQPVGWRCPYGYVQRWWGPFNIPGCGSCYIPICCRRRWYP

>Rf_EDM5
MARAMDNLPFDNLYHQEDESLSAFDSFRQYPGYCPPVQRPNWQCPPSYVQRWWGPFNIPGCGSCYIPVYCRRRWYP

>Rf_EDM6
MARGMEGIQLENLYHPEDESFPFFDSFSQYPGYCPPVQRPNWQCPPSYVQRWWGPFNIPGCGSCYIPVYCRRWYRQ

>Rf_EDM7
MARAMDSLPFDNLFHPEDESLSAFDSFRQYPGYCPPVQRPNWQCPPSYVQRWWGPFNIPGCGSCYIPVYCRRWYRQ

>Rf_EDM8
MALGMNGIQLDNLYHPADDSLSSLDSFCQYPDCYPPQRQKSIRCPPGCVVTWCPIYIKGCGYGYIPLCRPCYRQNNYYPGCRPC

>Rf_EDM9
MALGMNGIQLDNLYHPADDSLSSLDSFCQYPDCYPPQRQKSIRCPPGCVVTWCPVYIKGCGYGYIPLCRPCYRQNNYYPGCRPC

>Rf_EDM10
MALGMSGIQLDNLYHPVDDSLSFLDFFCQYRGCYPPVQHPGFQCPYGYVQKWYGPLIFPGCGCGYIPICCRPGYPQNNYYPGCRPC

>Rf_EDM11
MALGMNGIQLDNLYHPADDSLSSLDSFCQYPDCYPPQRQKSIRCPPGCVVTWCPVYIKGCGYGYIPLCRPCYRQNNYYPGCRPC

>Rf_EDM12
MALGMSGIQLDNLYHPVDDSLSFLDFFCQYRGCYPPVQHPGFQCPYGYVQKWYGPLIFPGCGCGYIPICCRPGYPQNNYYPGCRPC

>Rf_EDM13
MALGMNGIQLDNLYHPADDSLSSLDSFCQYPDCYPPQRQKSIRCPPGCVVTWCPVYIKGCGYGYIPLCRPCYRQNNYYPGCRPC

>Rf_EDM14
MALGMSGIQLDNLYHPVDDSLSFLDFFCQYRGCYPPVQHPGFQCPYGYVQKWYGPLIFPGCGCGYIPICCRPGYPQNNYYPGCRPC

>Rf_EDM15
MALGMSGIQLDNLYHPVDDSLSFLDFFCQYRGCYPPVQHPGFQCPYGYVQKWYGPLVFPGCGCGYIPICCRPGYPQNNYYPGCRPC

>Rf_EDM16
MTVDDSLSLFDSFCQYPCYYPPLQRPWWRFPRRYVPRWWGPCYPQWGCDTSYVPIPWGPCYPERYYPGGWPC

>Rf_EDM17
MTVDDSLSLFDSFCQYPCYYPPLQRPWWRFPRRYVPRWWGPCYPQWGCDTSYVPIPWGPCYPERYYPGGWPC

>Rf_EDM18
MAPGLNNILLGDFYHPEDDNLLFDSFCQYPGCYPPRQRYGCYYPGGYPQYPGCVMPYPKQWCDPYYPKQWCGPYYRQKGWGNCYSPKLYPGCGWPC

>Rf_EDM19
MALELDNILLGDFYHPEDDSLLFDSFCQYPGCYPPRQRYGCYYPGGYPQYPGCVMPYPKQWCDPYYPKQWCGPYYRQKGWGNCYSPKLYPGCGWPC

>Rf_EDM20
MAPGLNNILLGDFYHPEDDSLLFDSFCQYPGCYPPRQRYGCYYPGGYPQYPGYVMPYPKQWCGPYYPKQWCGPYYPKQWCGPYYPQRWCGPYYPQNSGGS
CYPSKLYPCE

>Rf_EDM21
MSLGMRSIQLDNFYHPVPDRLSAFDSFCQYSCCLPAPPPPSPPAWQCPPGYQRCLLGPYRLQNPCGIYYIPICWCPC

>Rf_EDM22
MSLGMRSIQLDNFYHPVPDSLSAFDSFCQYPCCLPAPPPPSPPAWQCPPGYQRCLLGPYRLQNPRGICYIPICWCPC

>Rf_EDGPC
MGSCESCCGGAEDSTIICVPNSRSNGGCQPCGGCQPCCCCSPCCCCQPCCYCQPCCNSCCTPQCSAQCCSTQCKQC

>Rf_EDGY1
MFFYRQLSCYEPCYEPCYQPCYQPCYQPCYQPCDYGYGIDYSRGVRCYDPCSSYGSCGYNSGISGNSSGDYSYASGSGTTCMPPCSYGYRYSQRGSCYMP
CGYRYGYGARVNSLSSSSRRSGRNYGRPC

>Rf_EDGY2
MSSFKYQSYCDPCDYRPISSSSCYGGFGRGSGYSSGKSYGGFGSRIRYSSGSCYGGYKLGGYGQGNSCISSGYGSGYGSGGYGSGGYGSQGYGGGCYQPI
SYGGWSGIQGYGRGCYQPISYGGWSGSQGSGRVCYQPISYGYGSGYGSGGYGSGGYGSQGYGGGCYQPISYGGWSGSQGYGRGCYQPISYGGYGDGGYGY
GSSGYGGRSSCYQPCYSYDSICGPC

>Rf_EDH
MTLGNGQHYGVLFNSLYNPTGDSCNISSLESFTYCHPHWLPGHHHHHCYYPGGYGWAPGCGSRWGPDSDWRYGPGHGYPRGCEPCYPLGGPRCPLGGPRY
SLGGPRYPLGGPRYPWGGVCYRRRGPCWLY

>Rf_EDMGC
MGCCCAHCKKQNDEHSCYEAHRHTACVPNPSCPQGGAPGPYQGGRFYPAPHCLPPQEGCKQDGAYPLQYDPVKS

>Rf_EDPCCC1
MSCCGSHRCSRCGCSPCCCCRTTCCSRCGCSPCCCRRVTCCSRCGCSPCCCRRVTCCSRCGRSPCCCGQSYSSSQQYKR

>Rf_EDPCCC2
MSCCGSHRCSRCGCSPCCCRRTTCCSRCGCSPCCCRRTTCCSRCGCSPCCCRRTTCCSRCGCSPCCCRRVTYCSQSCSSSQQYKR

>Rf_EDPCCC3
MSCCGSHRCSRCGCSPCCCRRRACCSRCGCSPCCCRRTTCCSRCGCSPCCCRRTTCCSRCGCSPCCCRRVTYCSQSCSSSQQYKR

>Rf_EDPCCC4
MSCCGSHRCSRCGCSPCCCHRRACCSRCGCSPCCCRRTTCCSRCGCSPCCCRRTTCCSRCGCSPCCCRRVTYCSQSCSSSQQYKR

>Rf_EDPCCC5
MANGECQSSCCSKCGCSPCRCQKSGWCPTCGRFPCCCDRVTCCSKCGRSPCCCRRVTCCARCGCCPCSCRRGI

>Rf_EDPCCC6
MSCCGSHRCSRCGCSPCCCHRRACCSRCGCSPCCCRRTTCCSRCGCSPCCCRRTTCCSRCGCSPCCCRRVTYCSHSCSSSQQYKR

>Rf_EDPCCC7
MANGECQSSCCSKCGCSPCRCQKSGWCPTCGRFPCCCDRVTCCSKCGRSPCCCRRVTCCARCGCCPCSCRRGI

>Rf_EDPCCC8
MSCCGSHRCSRCGCSPCCCHRRACCSRCGCSPCCCRRTTCCSRCGCSPCCCRRTTCCSRCGCSPCCCRRTTCCSRCGCSPCCCRRTTCCSRCGCSPCCCR
RVTYCSQSCSSSQQYKR

>Rf_EDPCCC9
MANGECQSSCCSKCGCSPCRCQKSGWCPTCGRFPCCCDRVTCCSKCGRSPCCCRRVTCCTRCGCCPCSCRRGI

>Rf_EDPCCC10
MSCCGSHRCSRCGCSPCCCRRSTCCSRCGCSPCCCRRTTCCSRCGCSPCCCRRVTCCSRCGRSPCCCGQSWSSSQQYKR

>Rf_EDP3
MSQQQQCKQTPSCPPTSCTPPSGCVKPYPPQTCPTEPQCPGKPFPGSTGSHGQCQSGKDDGSYHQHDSRQSKQC

>Rf_EDPKC
MASSSNQQQCKQTPTLPPALCKTVPEPSPCPPVVKIPQCPQEEHQCKQTITFPPVTCPQPTPCPPVEQPCKEPPVVVIPTPCPQPTPCPQQKTPCKEPPV
VVIPTPCPQPTPCPQQKTPCKEPPVVVIPTPCPQPTPCPQQKTPCKEPPVVVIPTPCPQPTPCPQQKTPCKEPPVVVIPTPCPQPTPCQEKPPCKEPPVV
VIPTPCPQPAPCQEKPPCKEPPVVVIPTPCPEPTPCPQEKPPCKEPPVIVVPIPCPQPEPVPCPQVKQPCKKPPVVDIPTPCLEPTPCPQKNSPCKEPPV
VVIPTPCPQPIPCPQEKPPCKEPPVVVVPIPCPQPEQVLCPQVKQPCKKPPVVVIPTPCPLPTPCPEEKPPCKEPPVVVIPAPCPEKQPPCKEPTPVPCC
PPSAKCPPPTDQQQRKQPCQWPPQK

>Rf_EDPL
MSSQQPQCPTNPPPCKDNQEKCPSKQDPACWPSKPCTLPQDQQKKSPPTCPQAPK

>Rf_EDPQ1
MHTSGCDDGQGSYNNYGRSVCSEPSCGTSGASCDPCHSVRWTAEVDCCSVPPQQYCPPVQKYCPPQQKYCPPVQKYCPPVQKYCPPVKKCYPPQQKYCPP
VQQCCPPVQKYRPPVEKYCPPVQKYCPPVQKCCPPRQKYCPPVQQCCPPVQKYCPPVEKYCPPVQKYCPPVQKCCPPVQKCCPPVQQCCPPVQKCCPPVQ
QCCPPVQKYCPPVQKYCPPVQQYCPPVTKCCPPVQQCYPPKQKYCPPVQQYCPPVQKCCPPLQQTCTSIGEPDICQIQEICQAPPRLLKK

>Rf_EDPQ3
MSYQDQQCKQPCPPPQCKQTIPPPQKCCPPPQQCCPPPQQCCPPPQKSCPPPKQCCPPQQSQDPKCC

>Rf_EDPSQ
MQCYEQQCKQACLPPPICQQKTGGKGKSGCPPQYKHQSYQSFTPEYAPPYPPQRQQSSAQQYIQPYSSQWQTSYAPQSIQSGAPQQQQSYGAPRLPESQQ
YLPAQYTQQCPQELLFSSSQGIQKPSSKSVSFAYQQPCAPKGKGQSASKGFQEYDTIQRSQACTVKGRQPPVTKGRQQYASQQGISKGGPGRTTEGSQAD
ASQLGISKGGPGRTTEGSQADASQQGITKGGPGRTTKGSQADASQQGITKGGPGRTTKGSQAYASQQGITEGGPGRTTKGSQAYASQQGITKGGLGYTTK
GSQAYASQQGITKGGPGRTTKGSQAYASQQGISKGGPGRTTEGSQAYASQQVITKGGPGRTTKGSQAYASQQGITKGGLGSQQYVSQQRATKGDPAYATK
SYQQGPSQQFNTKDGPINSTKGSQQYSRKGGQSSEVKISTSGEKYCSADDWLW

>Rf_EDQK
MSCPNQQQKQQSSVPPCSKQCPLKQNPQTCPLKQNPQTCPNKPSCGGKPLCSAGKPLCSRSKQTPCQEEQGSK

>Rf_EDQL1
MCSRNDRGCHNVPRDNSCHRGGSSCHDQGSCTKPIPGCQQQPPVVCPPPVVCPPPPCKQEPPVLCPPPVICPPPCQQQQQVKQPTQWPPQQQK

>Rf_EDQL2
MCSRNDRGCHNVPRDNSCHRGGSSCQDQGSCTKPVPGCQQQPPVVCPPPVVCPPPVVCPPPPCKQEPPVLCPPPVICPPPCQQQQQVKQPPQWPPQQQK

>Rf_EDQL3
MCSRNDRGCHNVPRDNSCHRGGSSCQDQGSCTKPVPGCQQQPPVVCPPPVVCPPPVVCPPPVVYPPPPCKQEPPVLCPPPVICPPPCQQQQQVKQPPQWP
PQQQK

>Rf_EDQL4
MCSRNDRGCHNVPRDNSCHRGGSSCQDQGSCTKPVPGCQQQPPVVCPPPVVCPPPLCKQEPPVLCPPPVICPPPCQQQQQVKQPTQWPPQQQK

>Rf_EDQL5
MCSRQEQGCCGTPLKPSCHSGGNSCHSGRSSCYCGGSSCHGSGTSCRWGGSCCHGSETFCHCHESSCHSGQSSCCRPRVEFCHNVRPLPQIQVPLSPPAV
IYPPCTHQQVKQPCQFLPQK

>Rf_EDSC1
MSQQQQQSGSCCCGGGGCCGGSGGGCCGGRSHGGSGGCCGSRVGGYSQQSQSSGGCCGGRSSGGGCCGGSSGGGGCCGSSGGGCCSGQQKVYPQKLK

>Rf_EDSC2
MSQQQQQSGSCCGGGGGCCGGSGGGCCGGRSHGGSGGCCGSRVGGYSQQSQSSGGCCGGRSSGGGCCGGSSGGGGCCGSSGGGCCSGQQKVYPQKLK

>Rf_EDSC3
MSQQQQQSGSCCGGGGGCCGGSGGGCCGGRSHGGSGGCCGSRVGGYSQQSQSSGGCCGGRSSGGGCCGGSSGGGGCCGSSGGGCCSGQQKVYPQKLK

>Rf_EDSCP1
MVFTYAFPFGATPFEPITLLCDQSYGVKSCSIPSSCVPCGPTIPVSSCQPCSVQSCAVPCGPVIPVSSCQPSSVQSCAVPCGPSIKVSSCQPSSVQSCAS
PCGPVIPVSSCQPSCVQPCAIPCGPTIKVSSCQPSSVQSCANPCGPVIPVSSCQPSCVQPCAVPCGPVVPVSSCQPSCVQPCAVPCGPVIPVSSCHPSCV
QPCAVPCGPVIPDSSCKPACHQTCVIPSGPIYPVSSCQPSYVPVSVQSCNPSCHPC

>Rf_EDSCP2
MASCCLPSGTIPCVPIRCSPRPSPSCAYPCGPIFPVSSSQPSRRQTYQCRPLAPLCVQPSSGQSSSGPTCAIPCGPIYPVSSGQSSSGPTCAIPCGPIYP
VSSGQSSSGPTCAIPCGPIYPVSSGQSSSGPTYAIPCGPIYPVSSGQSSSGPTCAIPCGPIYPVSSGQSSSGPTCAIPCGPIYPVSSGQSSSPPVSSCQP
CYVPICIEPCAPCRFPC

>Rf_EDSCP3
MASCCLPSCTIPCRPVIPIRSSPPPSPSCAYPCGPLAPLCVQPRSGQSSSVPTCAIPCGPIYPVSSSQSSSGPTCAIPCGPIYPVSSGQSSSGPTYAIPC
GPIYPVSSGQSSSGPTCAIPCGPIYPVSSGQSSSGPTYAIPCGPIYPVSSSQSSSGPTYAIPCGPIYPVSSGQSSSGPTCAIPCGPIYPVSSGQSSSSPV
SSGQPRYIPIRIEPCTPCRLPC

>Rf_EDSCP4
MAFCCVPSSCIPSGQISGSQPSSHPSCAIRFELTYPGSSSQHSSGQTSSSGRVAYLCLQPSSGQSSSLPMSSGQSSSRSSSCTPCVAILPVSSGQSSSFP
ASSGQSSSPPCYVIPCGPINAASSGQSSSQPSSSTPCVAILPVSSGQSSSFPASSGQSSSPPCYVIPCGPINAVSSGQSSSQPSSSTPCVAILPVSSGQS
SSFPASSGQSSSPPCYVIPCGPIDAVSSGQSSSQSSSSTPCVAILPVSSGQSSSFPASSSQSSSLPCCIIPCGPINPVSSGQSSSQPSSGIPCVAIVPVS
SGQSSSPPVSRGQPCYVPICFEPSSPCRHPC

>Rf_EDSCP5
MAFCCVPYSSIPSGQISGRQPSSHPSCAIQCELTYAGSSSQHSSGQTSSSGHVAYLCLQPSSGQSSSLLMSSGQSSSQPACGIPCVAILPASSSQTSSFP
ASSGQSSSPPCYVIPYGPIDAVSSGQSSSQSSSSTPCVAILPVSSSQSSSFPASSGQSSSPPCYVIPYGPIDAVSSGQSSSQSSSSTPCVAILPVSSSQS
SSFPASSGQSSSPPCYVIPYGPIDAVSSGQSSSQSSSSTPCVAILPVSSSQSSSFPASSGQSSSLPCYVIPYGPIDAVSSGQSSSQSSSSTPCVAILPVS
SGQSSSFPASSGQSSSLPCYVIPYGAIDAMSSGQSSSPPASSGQSSYAPVSSGQPCYVLI

>Rf_EDSCP6
MAFCCVPYSSIPSGQISGRQPSSHPSCAIQCELTYAGSSSQHSSGQTSSSGHVAYLCLQPSSGQSSSLLMSSGQSSSQPACGIPYVAILPASSSQSSSFP
ASSGQSSSPPCYVIPYGPIDAVSSGQSSSQSSSSTPCVAILPVSSSQSSSFPASSGQSSSPPCYVIPYGPIDAVSSGQSSSQSSSSTPCVAILPVSSSQS
SSFPASSGQSSSPPCYVIPYGPIDAVSSGQSSSQSSSSTPCVAILPVSSSQSSSFPASSGQSSSLPCYVIPYGPIDAVSSGQSSSQSSSSTPCVAILPVS
SGQSSSFPASSGQSSSLPCYVIPYGAIDAMSSGQSSSPPASSGQSSYAPVSSGQPCYVLI

>Rf_EDSPR1
MSCYQQKCKQACLPPPMCMKTCSPRCVDPCQLCPPRQCVKVCHPQCGCQCIPVCGPACGPTCGPARGPACGPPCGPACAAPCVKKCADKCMDPCSPKWK

>Rf_EDSPRL1
MAYQFQQCKQPCLPPPNCCPKGDTKYGDVFPPVCPVPCGPSCIQPPPDKSMSACPPPCSDPCAKIIGEPCAPVGTTPYAPKCNDAYTPKCVDPCAPKCVV
PCPEPCQSVYAPPCVPVGIDSCASKCVPVDQPCKQRPC

>Rf_EDSPRL2
MSQCKQGCKAPPTKTVPCCPPAVKCPPKTVPCCPPVVKCPPKTKQPPKGCC

>Rf_EDSQ
MSYQCKQPCLPPPSCMKGTTVCAEPGGAICVTPSQTSCADVGAPKCATVCTGPGGSICMAPGQGQCATVCQGQSNSVCVTPQRPQCATVCAGPGGSVCVT
PQQTQGPTVCATPSGSVFVPQGQSQCTTVCQGAGGSVCVTPQQPQCATVCTGPGGSVCVTPQQTQGPTVCATPSGSVFVPQGQSQCATICQGAGGSACVT
PQQPQCATVCTGPGGSVCVTPQQTQGPTVCATPSGSVFVPQGQSQCATVCQGAGGSVCVMPQQPQCATVCQGPGGSICVTPQQTPGGSVCVAPGKGQCAT
VCQDPCGNVSITPCETKCGAPGATVCTSTCGAAACVKTCATKGGNPGVTVCSDQVSVKSCPSINMGQCNVKKS

>Rf_EDSRWM
MTGFSLLSCYPSSGLQNIYRPQSICYPGYYNPCSYGYLTSNGSSNSQTSCYTPLCYSFLSGCGESCYKPCGYGYSSSYGSGSCYEPHGYGYSSSYGSGSC
YEPHGYGYSSSYGSGSCYEPHGYGYSSSYGSGSCYEPHGYGYSSSYGSGSCYEPHGYDYSSSYGSGSCYEPRGYSSQSSYGRNCSGPCSYGYLSGSGRSC
YEPSVCSSPSGDGGSCSEPHGYRYISDDAGTCYRPRGYRYLSRYSGRCYEPRGHNSPSGYDKSCYGSRGYGYLSSQDGSCDNPCGYSSPSSYGKSCYDPC
GNGYMSSNGGSCYDPCSDSCAPSCRQPRYSYRRRYSCRPLSSDPCSYDSGSSDPSSHRRNQKERSSSCGPC

>Rf_EDWM
MSEEGVIYSSGREPYYNLNSTWYDPAGSWLDTRRKPFRYADNTACVTCCNPRCDIPRRGGHDYRCYCYCCSTCESGCNSRVTCCVHNPSGGPRDYWGRPI
GDACNGCTGGHYSNEDSCCGSCGGTSGGCGAGQGGACAQPSVLAGGCGGGRGVCSEPGCRSSGGCGGGRGVCSEPGCRSSGGCGGGRGVCSEPGCHSSGG
YPPQGTGVCAEPLSSYSGGCGNEGRASGGC

>Rf_LOR1
MSSGQQKQSTSSCWGSSSGGCSSCGGSSGGGSSSYPSQGKLSDCCWGGSGGGRSSGQKTITISGGSSGGGGGQSCCGGSTGSSGGSGQIAICSRGGGGSG
QQSSGCCSGGGSSGGSGGGICQQKIPSLSGGGGICCGGGGSGGSSGVKMIGGGSGGGCGGGGTCGGVKIIGSNSSSGGGFGIGGGSSGGVKNIGGGSGCG
GGSGIKVVGAGSSGGGSICGGGSSGGSGGKTITVSGGSSGGQSSGGCIGGGSGGGGGSGGQTVIVPCGGGSGWGGQTVIDPCSGSSCGGSSGQSSGCSIG
GGSGSGGSSGGQSIIVPCGGSSGGGQSSGCFTGGVSGAGSSGGQTIIVPCGGSSGSGSSGQSSGCCIGGGSGSGGSSGAQSIIVPCGGSSGGGQSSGCFT
GGVSGGGSSGGQTIIVPCGGSSGSGSCGQSSGGCIGGVSSGCGGSSGQTIILSGASSGGGCGAGQSSGNCIRGGSGGGSMQTKQPVCLPPGIGQTKQSCQ
WPPSQK

>Rf_LOR2
MSKQKQTGGSCCCCASSGGGCCCCASGGGRRCCCCSRGGGYGQPIIVVPGGGSCCGGGGGGGYQQGYYGGSAGGLLLPDGGDGIGQQKIPLIGGGGGSVC
CSASGSGGSVCCSSGGSSGGLKVSGPGVGSGGPVCCSSSGRGGSVCCSSGGLPVVSSGGGSGGSVCCSSSGSGGSVCCSSGGSSGGQKVVGLGVGSGGPV
CCSSSGSGGSVCCSSGGSSGGLPVVGPGGGSGGSVCCSSSGSGGSVCCSSGGSSGGLGVIGSQTKCPIVVPPCLEQTKQVCPLPSIK

>Rf_LOR3
MSKQKQTGGSCCCCASSGGGCCCCASGGGRRCCCCSRGGGYGQPIIVVPGGGSCCGGGGGGGYQQGYYGGSAGGLLLPDGGDGIGQQKIPLIGGGGGSVC
CSASGSGGSVCCSSGGSFGGLKVSGPGVGSGGPVCCSSSGRGGSVCCSSGGSSGGLPVIGPGGGSGGSVCCSSSGSGGSVCCSSGGSSGGQKVVGLGVGS
GGPVCCSSSGSGGSVCCSSGGSSGGLPVVGPGGGSGGSVCCSSSGSGGSVCCSSGGSSGGLGVIGSQTKCPIVVPPCLEQTKQVCPLPSIK

B

>Rf_Crnn
MSQLLGNIDSIISAFNKYAKSDGDCATLTKGELKHIIQKEFAEVIVNPHKPETIETILQLLDKDFDGKVDFQEFTVLVFKVAKACYKKDQECQGTEGQPK
KSGSSRRQQDASAPDRDSTRQASREQEKASDADSQCSSQDGKWEPHKSDSCCDKELLKREQEPTSRSPQKPLGVCPNQKETKEHLRAEKEPKHRASRVER
RAEHCCTKPLQKEPQTVDEDLNGRSPQEALRLDEDSAQETQRSKVRQSSQLHKREEQGCTGPHKPEQDQPGQQGTHEREQTLSQRDTHKPPKKEEDVVKP
KSQGTVGRAYQRQEQEPQASRAIQDHQAERSSSGCQEGRASMFEQHSTCRREQGPQRGERSTRQQGCNPKTREQDPNSKPAEEHQETEGDSCEQPTKEIQ
GSERARSCQPGCRFPTHGKQLTRPQECQSRGPKQASTGREEQESQAPEEAATSHEECQSLTAEQRQAHREDQKPKNAEQNQYRCHECPPLTPEQDLSPQE
ETKPQSSEKEASCEEAQTSEKVTTGHQSLTRKQHQPCSEETKPQTSGHSQYGHHECQSPEQAPSSQEETKPQTSKTAASCEPTETPEKATAGLQKDEPLT
SGQCKPPQDKKKPQNSGQGQYRSDKCRSLTSEQAHSPQKDTKPQTQEREESYESAETPEKTTTGHRECRSLTRRHHQPCSEETKPQNSGHCQCRRHECQS
PEQAPSSQEKTKPQTSKTAASCEPTETPEKATAGHQKDEPLTSGQCKPPQDKRKPQNSGQGQYRSDECQSRTSEQAHSPQKDTKPQTQEREESCESAETP
EKAPTHHQEDESLTSERGKPPQDERKPQNSGHSQYQCNECQPLTPQQALSPQEETKPMVSETEACCKPMERAAAGHHKCPSSIPRHHQPHPEERKPRSSG
QSQYRCHCDCCSLTPEQAPSPQPGMPLQTQEREANCGLMETPATQGPRESLARQPFRSAWPQQHRRVLQFPSPWSPKH

>Rf_Scfn1
MPHLLDSICTIIGVFYKYAQQYGDCSTLNRREMKKLIQKEFAEVIKNPSDPETVKLTFQLLDINGDSLVDFNEYLLLIFKVAKACYSHLQPRECLIPKGE
TSRGPREGERWKDGQDRHQLQEDEREGNYVRERQDSDRAHLRQTEASTRGEERRDYLPWKPVAQEGERNWQAHHQKLRDDRGRDHPSRGPQEWENVYEGR
PREFEMQGEVESQWHAHEHDPQRDEAGRHQLRQREPEGGRQHQPRENVSRDYHERRLPREPLPREGDEREQYSQEATERGKNERQHQLQESERQAEEDLT
RPREFGPWVGEESCRHPRAAQKIRSNYGRCRPSHESQEREGGRREDYPRERETEDEARSRDLDWRVADEHWDQRRGREPVDSYDDFGPLPREGERRSRSH
DLERKQRRSETREPERRERSWASDPEHRAVEWTRQNQYAPQSREAEQHEPEVREAERRRQTQSVDQDEWTRPRTREPTTRVEVEDQRWSREPERRQREQS
GLCPYKPEPTDCERRRPRSHEGDRRRSQQHAQTPNEEDCRRQPCRQDEAQEEENQRRRPMPAELASSDRESRRPQAREQEPREDGQRQQQYYDTEPVERD
GERRRPDARGPAQREGSRGRQQYARSEILERDGERRRCQPRDSEGERRHQQTGFRDDEWERPQSREPRTRPREQDACYPSKGGLIDCDQNRQRTYKSKPW
EGERRTHRLGDPEHEDYEERSQMYCEPGVRRHERSRPLQRQTQSPNTDLIDVDERQTQTWEIDPRDGEQQRRSQFRDTEESDSKRSWTLIHDSEARDGDQ
RRDRLCDIDQQDDEQERRTHFHDADVRDVDQRRRGTDPRMGEQRRRVPSRDIDAADIDQRQTQMSDFDPRDDETQRRAQECDIGVRDVVQGRTHSRDGEP
GDREQQRPTQSCAIQPIEVGQRQAQSLEVGPRDGEQRRRNPSQDTEQRQTQRHDVDRRGNEQQRGTPSCDIDAGDIDQRRSQSHDVEPRDNEQQRRAPSC
GTDARDSDRRQTQRYDVDQRYDQRQRRALSHVLDAGDADQRQSQSHDVEPRDYEQQRRTQSHNTVARDGGQRPALTRDVDRRDGEQHQRTPSWETDARNA
DRQRTVSHEVDPRGSEQERSTQSRDTEQRDSQQNRTQSQQRDASDGEQRRTHSGDTDPRDGEQRGRTPSQDTDVTQRQRQTHGIDPREAEEQMRTQSCET
VTTDVDRQQTKALEADPNDCEQQRQTQRWAHEADPRDSEQQRPTQSCDPKRRDVDQVTAQDAAQEPEPKDDIQRRQGEPETQAAQSSCAQPLKPLQQEGE
GSRAHSHEAAPMTDTPRRPQRQELKPAEGALEGQGVAESADAQQSQVRRERAQPREPQRSEREQGRARPRGPELRQATRGSQCPRGPERSGTERNLPQLH
HHGPQEGDSRRQQRQDPGSPAGKATTSQPCEPQATEGQRGKPQGREPNPEPKVKGKPDAPDKRDGAQVQQPGAPEQRQPPVLGASHEGDGGWPASTEPPS
EEGSDRSSPQAREAQPPLEEEGQPGEGRAESESKPESSATDASQEKPGFLCDKMPLVCNPLYQYLLAQKKQEQH

>Rf_Scfn2
MAYLLDGICTIIGVFYKHAQCQGGSFSLTRREMKRLIQKEFVEVLRNPSDPETVELTFQLLDINGDSLVDFNKYLLLIFKVAKACYSHLQPRECLFRKGE
ARCEGERWRDGQDCRQLWEDEREERRDSDCARLRQTEASTRGENPIAHEGERNWQAHHQKLRDDRGRDHPSRGPQEWENVYEGRPREFEMQGEVESQWHA
HEHDPQRDEAGRHQLRQREPEGGRQHQPRENVSRDYHERRLPREPLPREGDEREQYSQEATERGKNERQHQLQESERQAEEDLTRPHEFGPWVGEESCRH
PRAAQKIRSNYGRCRPSHESQEREGGRREDYPRERETEDEARSRDLDWRVADEHWDQRRGREPVDSYDDFGPLPREGERRSRSHDLESKQRRSETREPER
RERSWASDPEHRAVEWTRQNQYAPQSREAEQHEPEVRGAERRRQTQSVDQDEWTRPRTREPTTRVEVEDQRWSREPERRQREQSGLCPYKPEPTDCERRR
PQSHEGDRRISQRHAQTPNEEDCRRQPCRQDEAQEEENQRRRPMPAELASSDLESRRPQAREQESREDGQRQQQYYDAEPVERDGEQRRPDARGPAQREG
SRGRQQYARSEILERDGERRRCQSCDSEGERSPQQTGFRDDEWERPQSREPRTRPREQDACCPSEGSPRDCDQNWQRTYESKPWEAERRSHQLREPKPER

GARDLSRQTCCEPGAIGRERRALPRGSEHRLPEQRKPQRRDLEARERGNKRQDVNRNNAI

>Rf_Scfn3
MAHLLDSIHDITGVFYKHAKRHGGSVTLSQREMKRLIKEELAEVIKNTCDPQTLQLMFQQLEHNKEGVVDFNSYLHLLFNTAKACFRHQEARGCFLPCDG
SRRKEERRDYLREPVVQVDEGNCCVCQLELRGDCRKDCPPHDHPEREDECEDQCREFECQAIVENCGQSREQELQRNEASCRDLLQRDDLERCHPLCQS
QLREGDERDQCSIRRGEDRRQCQRQETEEENMRRPWLCGSGEGEGGRAQPRRRDDEPNKESFQSKSLQRDCERRRPQICEATLREAIQRKEDCYEALEKG
CKSRKSQQVCYNESVDCEKRRSWSCEQALWDEVQRSLEHYEHELLENANDGRFQACKPGVCEDDQRKQGFYDPGTFEGDVEWRRSEACKPGLCEDVQKKQ
QYCDPEAFERDAERRRPQTCKPGLREDSQKQQQQCEIGSFKKGAGRRRSARRQPQLCEDVQKKQLFCDPETFERDTEPRKSEACKPEPCEDRQHHQCYEA
GAFKRGMEQRRSLRHERVCQEDIRGRQQCFQPEPMECDGERRRLQGCESGLRECIPKKQLYCEPGALDRKCERRRPLLRVEKVRRWDLKSANLP

C

>Rf_EDKM
MSKLFKAFTDMIEGNSKANHKKVNESEKCKQSEFKKLIHQEISPVTRTSSNKYKHMKKLPDSDVELMNDKEVAPCVY

>Rf_EDYM2
MDDAIPSPPLIPSPPLNFSHRYHRASQKVPYYGYHPGAPLPPFVKKRLPKYSSQYIPLYGLRSPPSKNIPSHGKDPYAPLYIPPGVTNYQPWNATVRPVP
HAAKYPKQNLIKDPKPPYADVYQKPLPFTIPYNGMTKGPHGTINKGSLPCVTSTPLPRMSKGPVLYSANASQPCINKVPLPRVTKGPVLYATKNPLPHTI
KDPLARVNKGPVLYSAKGPHAFTAHLSQVNVTKGTQPCTVKDTLPRTTKGPRLVVTKVPRFSALRASRSFLKRGSRPNLTKGSQPCLVKGSRPSLAKGSR
FSLAKTSQPSLAHTSMHPAPKKLSKNVKITSTGKKYCSATKWPF

>Rf_PGLYRP3
MVKQEILLLVLSALSQAMGCFQLITPSKWGAKPANCSAPLKDLLPEYVIIIHTVQNPCKTPAECAREVRNVQDYHLHLKEWCDVAYNFLIGEDGLVYEGR
GWRSEGSHTYGYNDLSLGIAFIGTFTQRSPNEAAWKALKCLLDFSVKIGYLSPDYLIMAHSDISDVVSPGEPIRSEISKWPNYRHN

>Rf_S100-A10
MPSQLEHAMETAMFTFHKYAGDKNYLTKEDLRQLMEKEVPGYMENQKDPMAIDRIMKNLEECRDGKVNFEGYLSLFAGLTNGCNEYYVKKMKPSGKKY

>Rf_S100-A11
MLSKQACLALSFQVIAQAGQGREGGRLSLQRESRALAGPRRSQRASHRPSDPSLRASQPSSPPASQLSGPRRLSLSPPSLSTAMSSRYTVGPTETERCIE
SLLAVFQRYAGRDGDASALTKKEFLTFMNTELASFSKSQKDPAILDRMMKKLDMNCDGKIDFGEFLNLIGGMAQACHAHVLSSPTGGLPQKP

>Rf_S100-A12
MSKAKTAMQIVADQLIDIFHKYAGQSDDLDKKEFKKMIDEQFPDCVEYPRKQEGKDKLFKDLDKNKNDRISFEEWTTLLGSFLTCSHIKFHQQHGDHHHH

Supplementary Figure S1. Amino acid sequences of proteins encoded by EDC genes of the Florida worm lizard. **(A)** Amino acid sequences of proteins encoded by the Florida worm lizard single coding exon epidermal differentiation complex (SEDC) genes excluding corneous beta proteins (CBPs) except for the first and last one. **(B)** Amino acid sequences of S100 fused-type proteins (SFTPs). **(C)** Amino acid sequences of proteins encoded by other EDC genes of the Florida worm lizard. Several amino acid residues are highlighted to indicate residues that are either important for protein cross-linking or highly represented in the amino acid composition of SEDCs and SFTPs: cysteine residues (C) are potential sites of disulfide bonds; lysine (K) and glutamine (Q) are potential sites of transglutamination; glycine (G), proline (P) and serine (S) are highly abundant residues not directly involved in cross-linking. Only S100A proteins flanking *PGLYRP3* and *Scfn1* are shown here. Rf, *Rhineura floridana*.

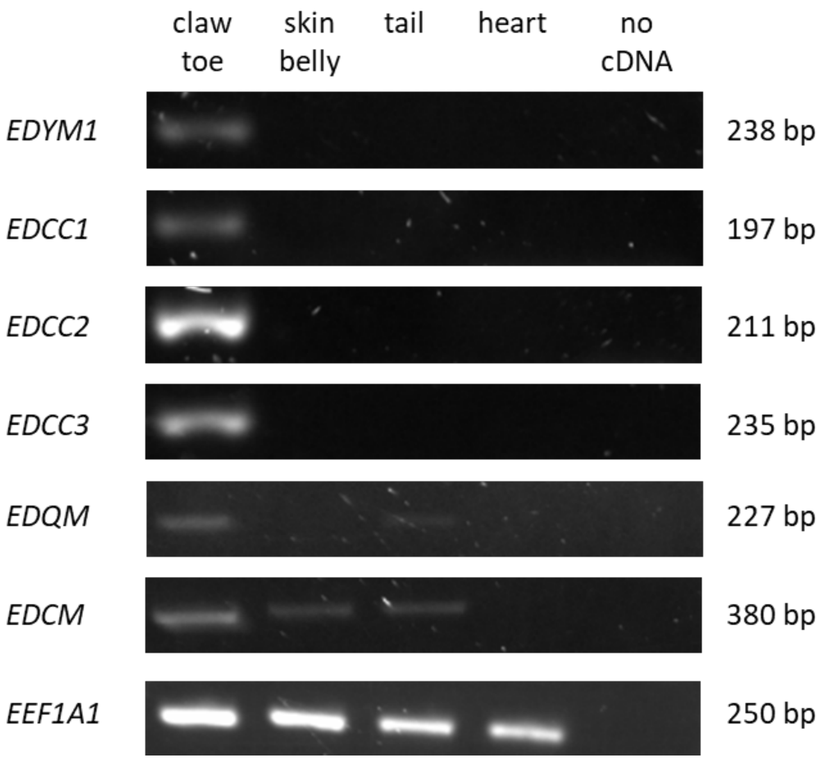

Supplementary Figure S2. Putative claw-associated EDC genes are expressed in the clawed toes of the green anole lizard (*A. carolinensis*). mRNAs from the indicated tissues were subjected to RT-PCR analysis with primers specific for EDC genes and the house-keeping gene *EEF1A1*. PCR products were analyzed by agarose gel electrophoresis in comparison to a DNA size marker. Lengths (base pairs, bp) of products were in agreement with the predicted size, which is shown on the right. The identity of the products was confirmed by Sanger sequencing.

A

```
>Ac_KRT84L1
MSFHSIPACGPRNFSSCSAVLQHPGNTSRISDSCSSRISGISCGGAGCFGTRSLGGIGSCGPRIAIGNCPPPRCRYSCCGAGYGCGYGHSGICMPCSPNI
TRVTCNQDLLEPLNLGIDTTAQATKCQEKNELQCLNSKFASFIDKVQFLEQQNLMLKTKWDFLQERKCCKSNMEPMFKEYIANLKKEQECMECERAQLQA
EMKNWREALEVNKKKFEEECHRRTCTENEYVSIKKEVDCVFMDKSEKEAKVQALMKDLFFYKPTFYQEIHELQSCISDTCVTVQMDNSRGLNMDCVMEVY
RRQFEEISSRSRAEAEAWCRCQYQELKTTAAKHCDNLRHVKEELSEITRVVHRLESEVSNVKAQCTKLEEEVAAAEERGGIAVKDARCKLVELEDALHKA
KQDMACQLREYQDLMNMKMGLDIEITTYKKLLDGEELRMGDGDCAVNISVRRSQGAVVGGSDPRPPCGPHVLCGPNKRTVTTCGPSRERCIDTMLPCTAA
HCMNTCSPSGDLCSLPRGFSCESGKCSNIKCVTSIGPYGQRY

>Ac_KRT84L2
MSSPPSPAKRPSNFTSCSALLPSTESRNYFGFPSTSKGYSSGFKGAGYFSSQSLSGSSSHGPKITGASCHPAIYGYGHRGIGAGYGYSGTGCSYSLYGPY
GFNQAGIGTSGTPAITPVICNMTLLQPLNLEINSAAQAVKCQEKNQLQCLNSKFACFIDKVRFLEQHNLMLKTKWDFLKEKKCQKSNMDPMFHEYISRLR
KELERLQWERNQLQVEVNNWRDTMEGNKKKYEEEYNRRASAENEYVTLKKEVDYIFMEKSEKEAKVETLMQDIFFYKTTFEEEICEMQSSISDTCVTVKM
DNSRDLDVACIIEEFRCRYEDIASRSRAEAEAWCQCQYQELKTTAAKHSDNLRNFKDELEELTRTVNRLQAEIANVKAQRCKLEEEVAGAEERGEMAVKD
AKCKLFDLEDALHKAKQDMACQLREYQELMHLKLALDIEIATYRKLLEGEECRINDGECAVNISVQRSQGAVVCNSDLIGGPKRGGSIMMNGRDTCNPAG
YKQSLSSCGAPCAVQSPPGGDMCNPPPPPQPHGIKSGKGPNVNVGATPAPCASRG

>Ac_KRT84L3
MPYHINPGCGPRNYNFYNGGQGPASHGFCPPIDTGMCNGGTGGFGSQSVGSCGPWPAFKNSFPGNGHPGNVGAGYNHCGIRPSHVFMNTGGAGAPGQSRI
TSVTCNESLLKPLDLGIDTEALAEKSETKNELQSLNSKLASFINKVQLLEQHNLMLKTKWDFVQEMKQHRSDMEPLFDDHTSRLKKELECLEREKKEMQI
EHDSSAQTLEKNKSRYEEELNKRATAENGFVLLKKDLDFAFADKAELEAKVEKLAKHISFLKHIYAQEISELQNCISETCVMVQLDNRRALDMNRTIEEF
RRQHEYIASRTRAEAEAWLQHQYQELKTTAAKNNDNLNAVKEEIQALTRTAHQLESQITSIKTQRCRLEDEVVEAKEHGETAVKDARCKLSDLEEALRKA
KQDMTCQLREYQSLMNVKMAMNIEIATYRKLIEGEECWLNRGDRAVNICVQRSEGAIVSGGNFHHGSGRTLPCETKTSKKDVHDSDKATPGSSVTAKSSP
GDAGGSGDVGGSSSSSSPVPH
```

B

```
>Ac_EDCC1
MSCCGGGCCGGCCGGSYGGGYGGGHGGAGGGGGSFVGYTLGRDIHVTPGECYMITPGPYLRDCCGVSVCCHCVCVPDCCPCGSGGYGGYGGYGGGCGGGG
LEVDIIGESRNITMPATYLCSRVHCAESCCTCCEPCCCTC

>Ac_EDCC2
MACCGSGCCGDCCGGGYGGYGGYGGGYGGDGGGVLAAVGVNKNVHVRDHGCTMVFPTPYITNNGDFRMCCREVCAHECCKPCCGGGGYGGYGGGDGGYGG
QGPGLRVDMRQAGSTVHVPGSYLFSCYPCASSCTECCDPCCCGSCCGPC

>Ac_EDCC3
MACCMSSCTSHGGGFGSNSDDGAVLVGWTSDKVVRVSPGRSSLLIPPPSAKTTNQPFRISCHIVRCLGVGNGEMVWLNRCNYG

>Ac_EDYM1
MTNCNYQCWPQNFPPQGPQFGPGPQCGPGLQCGPGPQCGPGPQCGPGPQCGPGPQYGMMKPSASFMNMNQPPFMNMNQPANFPEPYKMNFCNIQGSMPCA
PCGPAPCDSKYVESSDAKSTEKHASSCDTLSHESCTMKNSSQPTDRCSSQRPPCPSMPGMSLCSPSCGPSMGRPHGFPSSSQCFPPRNMYQYTTSKTFKS
CYAK
```

C

```
>Ac_EDMGC
MGCCCGRCSRRHNQNPTYEGHGQNMQGGAKGGASYSKGGSSGGQSEGYKGAPQPQYSSGSGQGGKS
```

Supplementary Figure S3. Update of amino acid sequences of proteins of the anole lizard. **(A)** Amino acid sequences of keratin proteins that were updated for this study according to the current assembly of the anole lizard. Keratin sequences of the anole lizard were analysed in Ehrlich et al. (2020). **(B)** Amino acid sequences of SEDC genes that were updated for this study according to the current assembly of the anole lizard. **(C)** Newly identified EDC amino acid sequence in this study based on similarity to Rhineura proteins. Other EDC genes of *A. carolinensis* have been reported in Strasser et al. (2014) and Holthaus et al. (2017). KRT, keratin**;** SEDC, single coding exon epidermal differentiation complex; Ac, *Anolis carolinensis*.

Supplementary Table S1. Genes of the keratin type I cluster of the Florida worm lizard and the green anole lizard

Species	Gene name	Gene symbol (GenBank)	Accession nr. of genomic DNA	Notes
Rhineura floridana	KRT222	KRT222	NC_084490.1	
Rhineura floridana	KRT24	LOC133367183	NC_084490.1	
Rhineura floridana	KRT24L	LOC133367217	NC_084490.1	
Rhineura floridana	KRT10L1	LOC133367216	NC_084490.1	
Rhineura floridana	KRT10L2	LOC133366031	NC_084490.1	
Rhineura floridana	KRT12	LOC133365869	NC_084490.1	
Rhineura floridana	KRT20	LOC133366034	NC_084490.1	
Rhineura floridana	KRT23	KRT23	NC_084490.1	see Table S2
Rhineura floridana	KRT36L1_mutated	n.a.	NC_084490.1	see Table S2
Rhineura floridana	KRT15	LOC133366142	NC_084490.1	see Table S2
Rhineura floridana	KRT19	LOC133366143	NC_084490.1	
Rhineura floridana	KRT9L	LOC133367211	NC_084490.1	
Rhineura floridana	HAS1	LOC133366002	NC_084490.1	
Rhineura floridana	HAS_mutated	LOC133368034	NC_084490.1	
Rhineura floridana	HAS2	LOC133365843	NC_084490.1	
Rhineura floridana	KRT14L1	LOC133367237	NC_084490.1	
Rhineura floridana	KRT14L2	LOC133367208	NC_084490.1	
Rhineura floridana	KRT117	LOC133368032	NC_084490.1	
Anolis carolinensis	KRT222	krt222	NC_085846.1	
Anolis carolinensis	KRT24	LOC100552076	NC_085846.1	
Anolis carolinensis	KRT24L	LOC103279244	NC_085846.1	
Anolis carolinensis	KRT10	LOC100552270	NC_085846.1	
Anolis carolinensis	KRT12	LOC100552468	NC_085846.1	
Anolis carolinensis	KRT20	LOC107982248	NC_085846.1	
Anolis carolinensis	KRT23	krt23	NC_085846.1	see Table S2
Anolis carolinensis	KRT36L1	LOC100337549	NC_085846.1	see Table S2
Anolis carolinensis	KRT36L2	krt36	NC_085846.1	see Table S2
Anolis carolinensis	KRT15	LOC100552862	NC_085846.1	see Table S2
Anolis carolinensis	KRT19	LOC100563808	NC_085846.1	
Anolis carolinensis	KRT9L1	LOC100553064	NC_085846.1	
Anolis carolinensis	KRT9L2	LOC100553253	NC_085846.1	
Anolis carolinensis	HAS1	LOC100553450	NC_085846.1	
Anolis carolinensis	HAS2	LOC100337544	NC_085846.1	
Anolis carolinensis	HAS3	LOC100553650	NC_085846.1	
Anolis carolinensis	HAS4	LOC100553851	NC_085846.1	
Anolis carolinensis	KRT14L1	LOC100564200	NC_085846.1	
Anolis carolinensis	KRT14L2	LOC100564396	NC_085846.1	
Anolis carolinensis	KRT117	LOC100554046	NC_085846.1	

Notes - Gene names were assigned according to the best match to the anole lizard keratins sequences in Ehrlich et al. 2020. Keratin sequences of the anole lizard from this paper were also used to check GenBank predictions in other lepidosaur species. HAS, hard acidic sauropsid-specific keratin; n.a.; not applicable. Species: Florida worm lizard (*Rhineura floridana*) and green anole lizard (*Anolis carolinensis*).

Supplementary Table S2. Genes of the KRT36L locus in squamates

Species	Gene name	Comparison to gene prediction in GenBank	Gene symbol (GenBank)	GenBank product name	Accession nr. of genomic DNA scaffold (GenBank)
Rhineura floridana	KRT23	same	KRT23	keratin, type I cytoskeletal 23 isoform X1	NC_084490.1
Rhineura floridana	KRT36L1_mutated	not predicted in GenBank	n.a.	n.a.	NC_084490.1
Rhineura floridana	KRT15	same	LOC133366142	keratin, type I cytoskeletal 15-like	NC_084490.1
Podarcis muralis	KRT23	same	LOC114582477	keratin, type I cytoskeletal 23-like	NC_041324.1
Podarcis muralis	KRT36L1	same	LOC114582628	keratin, type I cuticular Ha4-like	NC_041324.1
Podarcis muralis	KRT36L2	same	LOC114582553	keratin, type I cuticular Ha6-like isoform X1	NC_041324.1
Podarcis muralis	KRT15	same	LOC114582365	keratin, type I cytoskeletal 15-like	NC_041324.1
Thamnophis elegans	KRT23	same	LOC116522300	keratin, type I cytoskeletal 23-like	NC_045558.1
Thamnophis elegans	KRT15	correction of 3'-end	LOC116521238	keratin, type I cytoskeletal 15-like	NC_045558.1
Anolis carolinensis	KRT23	same	krt23	keratin, type I cytoskeletal 23	NC_085846.1
Anolis carolinensis	KRT36L1	same	LOC100337549	keratin, type I cuticular Ha4	NC_085846.1
Anolis carolinensis	KRT36L2	same	krt36	keratin 36	NC_085846.1
Anolis carolinensis	KRT15	same	LOC100552862	keratin, type I cytoskeletal 15	NC_085846.1
Gekko japonicus	KRT23	same	LOC107107338	keratin, type I cytoskeletal 23-like isoform X1	NW_015163643.1
Gekko japonicus	KRT36L1	same	LOC107107335	keratin, type I cuticular Ha4-like	NW_015163643.1
Gekko japonicus	KRT36L2	same	LOC107107334	keratin, type I cuticular Ha6-like isoform X1	NW_015163643.1
Gekko japonicus	KRT15	correction of 5'-end	LOC107107330	keratin, type I cytoskeletal 14-like	NW_015163643.1

Notes - Gene names were assigned according to the best match to the anole lizard keratins sequences in Ehrlich et al. 2020. Keratin sequences of the anole lizard from this paper were also used to check GenBank predictions in other lepidosaur species. n.a.; not applicable. Species: Florida worm lizard (*Rhineura floridana*), common wall lizard (Podarcis muralis), western terrestrial garter snake (*Thamnophis elegans*), green anole lizard (*Anolis carolinensis*) and Japanese gecko (*Gekko japonicus*).

Supplementary Table S3. Genes of the keratin type II cluster of the Florida worm lizard and the green anole lizard

Species	Gene name	Gene symbol (GenBank)	Accession nr. of genomic DNA	Notes
Rhineura floridana	KRT80	LOC133379509	NC_084482.1	
Rhineura floridana	KRT7	LOC133379508	NC_084482.1	see Table S4
Rhineura floridana	KRT84L1_mutated	LOC133382333	NC_084482.1	see Table S4
Rhineura floridana	KRT84L2_mutated	n.a.	NC_084482.1	see Table S4
Rhineura floridana	KRT84L4_mutated	n.a.	NC_084482.1	see Table S4
Rhineura floridana	ETAA1	ETAA1	NC_084482.1	not a keratin
Rhineura floridana	KRT5L3	LOC133379505	NC_084482.1	see Table S4
Rhineura floridana	KRT5L2	LOC133379504	NC_084482.1	
Rhineura floridana	KRT5L1_mutated	LOC133382323	NC_084482.1	
Rhineura floridana	HBS1	LOC133379507	NC_084482.1	
Rhineura floridana	HBS2	LOC133379502	NC_084482.1	
Rhineura floridana	HBS3	LOC133379500	NC_084482.1	
Rhineura floridana	KRT78LT	LOC133379499	NC_084482.1	
Rhineura floridana	KRT78L5	LOC133379498	NC_084482.1	
Rhineura floridana	KRT78L4	LOC133379497	NC_084482.1	
Rhineura floridana	KRT78L3	LOC133379496	NC_084482.1	
Rhineura floridana	KRT78L2	LOC133379495	NC_084482.1	
Rhineura floridana	KRT78L1	LOC133379494	NC_084482.1	
Rhineura floridana	KRT8	KRT8	NC_084482.1	
Rhineura floridana	KRT18	KRT18	NC_084482.1	
Anolis carolinensis	KRT80	KRT80	NC_085842.1	
Anolis carolinensis	KRT7	LOC100567055	NC_085842.1	see Table S4
Anolis carolinensis	KRT84L1	LOC100562411	NC_085842.1	see Table S4
Anolis carolinensis	KRT84L2	LOC100337546	NC_085842.1	see Table S4
Anolis carolinensis	KRT84L3	LOC100562208	NC_085842.1	see Table S4
Anolis carolinensis	KRT84L4	LOC100337545	NC_085842.1	see Table S4
Anolis carolinensis	KRT5L4	LOC134296307	NC_085842.1	see Table S4
Anolis carolinensis	ETAA1	ETAA1	NC_085842.1	not a keratin
Anolis carolinensis	KRT5L3	LOC100566287	NC_085842.1	see Table S4
Anolis carolinensis	KRT5L2	LOC100566088	NC_085842.1	
Anolis carolinensis	KRT5L1	LOC100561812	NC_085842.1	
Anolis carolinensis	HBS1	LOC100561620	NC_085842.1	
Anolis carolinensis	HBS2	LOC100561422	NC_085842.1	
Anolis carolinensis	HBS3	LOC100561224	NC_085842.1	
Anolis carolinensis	KRT78LT_mutated	LOC107982338	NC_085842.1	
Anolis carolinensis	KRT78LT	LOC100560827	NC_085842.1	
Anolis carolinensis	KRT78L5	LOC100560629	NC_085842.1	
Anolis carolinensis	KRT78L4	LOC100560433	NC_085842.1	
Anolis carolinensis	KRT78L3	LOC100560234	NC_085842.1	
Anolis carolinensis	KRT78L2	LOC100560037	NC_085842.1	
Anolis carolinensis	KRT78L1	LOC100565886	NC_085842.1	
Anolis carolinensis	KRT8	KRT8	NC_085842.1	
Anolis carolinensis	KRT18	KRT18	NC_085842.1	

Notes - Gene names were assigned according to the best match to the anole lizard keratins sequences in Ehrlich et al. 2020. Keratin sequences of the anole lizard from this paper were also used to check GenBank predictions in other lepidosaur species. HBS, hard basic sauropsid-specific keratin; n.a.; not applicable. Species: Florida worm lizard (*Rhineura floridana*) and green anole lizard (*Anolis carolinensis*).

Supplementary Table S4. Genes of the KRT84L locus in squamates

Species	Gene name	Comparison to gene prediction in GenBank	Gene symbol (GenBank)	GenBank product name	Accession nr. of genomic DNA
Rhineura floridana	KRT7	same	LOC133379508	keratin, type II cytoskeletal cochleal	NC_084482.1
Rhineura floridana	KRT84L1_mutated	identfication of further parts of the pseudogene	LOC133382333	LOW QUALITY PROTEIN: keratin, type II cuticular Hb4-like	NC_084482.1
Rhineura floridana	KRT84L2_mutated	not predicted in GenBank	n.a.	n.a.	NC_084482.1
Rhineura floridana	KRT84L4_mutated	not predicted in GenBank	n.a.	n.a.	NC_084482.1
Rhineura floridana	ETAA1	n.a.	ETAA1	ewing's tumor-associated antigen 1	NC_084482.1
Rhineura floridana	KRT5L3 (X1)	same	LOC133379505	keratin, type II cytoskeletal 5-like	NC_084482.1
Podarcis muralis	KRT7	same	LOC114593010	keratin, type II cytoskeletal cochleal	NC_041313.1
Podarcis muralis	KRT84L1	same	LOC114593009	keratin, type II cuticular Hb5	NC_041313.1
Podarcis muralis	KRT84L2	same	LOC114593008	keratin, type II cuticular Hb5	NC_041313.1
Podarcis muralis	KRT84L3	correction of 5'-end	LOC114593007	intermediate filament protein ON3-like	NC_041313.1
Podarcis muralis	KRT84L4	same	LOC114593005	keratin, type II cytoskeletal cochleal-like	NC_041313.1
Podarcis muralis	n.a.	not predicted as KRT gene	LOC114593006	keratin, type II cytoskeletal 6A-like	NC_041313.1
Podarcis muralis	KRT5L4	same	LOC114593004	keratin, type II cytoskeletal 6A-like	NC_041313.1
Podarcis muralis	ETAA1	n.a.	ETAA1	ewing's tumor-associated antigen 1	NC_041313.1
Podarcis muralis	KRT5L3	same	LOC114593001	keratin, type II cytoskeletal 5-like	NC_041313.1
Thamnophis elegans	KRT7	same	LOC116504324	keratin, type II cytoskeletal cochleal	NC_045542.1
Thamnophis elegans	ETAA1	n.a.	ETAA1	ewing's tumor-associated antigen 1	NC_045542.1
Thamnophis elegans	KRT5L3_2	same	LOC116504440	keratin, type II cytoskeletal 6A-like	NC_045542.1
Thamnophis elegans	KRT5L3_1	same	LOC116504932	keratin, type II cytoskeletal 5-like	NC_045542.1
Anolis carolinensis	KRT7	same	LOC100567055	keratin, type II cytoskeletal cochleal	NC_085842.1
Anolis carolinensis	KRT84L1	correction of 5'-end	LOC100562411	keratin, type II microfibrillar, component 7C	NC_085842.1
Anolis carolinensis	KRT84L2	correction of 3'-end	LOC100337546	keratin, type II cuticular Hb5	NC_085842.1
Anolis carolinensis	KRT84L3	correction of 3'-end	LOC107116752	keratin, type II cytoskeletal 8	NC_085842.1
Anolis carolinensis	KRT84L4	same	LOC100337545	keratin, type II cytoskeletal cochleal	NC_085842.1
Anolis carolinensis	KRT5L4	correction of 5'-end	LOC100562008	keratin, type II cytoskeletal 5	NC_085842.1
Anolis carolinensis	ETAA1	n.a.	ETAA1	ewing's tumor-associated antigen 1	NC_085842.1
Anolis carolinensis	KRT5L3	same	LOC100566287	keratin, type II cytoskeletal 6A	NC_085842.1
Gekko japonicus	KRT7	same	LOC107116752	keratin, type II cytoskeletal cochleal-like	NW_015170915.1
Gekko japonicus	KRT84L1	correction of 5'-end	LOC107116741	keratin, type II cuticular Hb1-like	NW_015170915.1
Gekko japonicus	KRT84L2	correction of 3'-end	LOC107116740	LOW QUALITY PROTEIN: keratin, type II cuticular Hb5-like	NW_015170915.1
Gekko japonicus	KRT84L3	same	LOC107116739	keratin, type II cuticular Hb5-like	NW_015170915.1
Gekko japonicus	KRT84L4	same	LOC107116743	keratin, type II cytoskeletal cochleal-like	NW_015170915.1
Gekko japonicus	KRT5L4	same	LOC107116749	keratin, type II cytoskeletal 6A-like	NW_015170915.1
Gekko japonicus	ETAA1	n.a.	ETAA1	ewing's tumor-associated antigen 1	NW_015170915.1
Gekko japonicus	KRT5L3	correction of 3'-end	LOC107116736	keratin, type II cytoskeletal 5-like	NW_015170915.1

Notes - Gene names were assigned according to the best match to the anole lizard keratins sequences in Ehrlich et al. 2020. Keratin sequences of the anole lizard from this paper were also used to check GenBank predictions in other lepidosaur species.ETAA1 is not a keratin gene. n.a.; not applicable. Species: Florida worm lizard (*Rhineura floridana*), common wall lizard (Podarcis muralis), western terrestrial garter snake (*Thamnophis elegans*), green anole lizard (*Anolis carolinensis*), Japanese gecko (*Gekko japonicus*).

Supplementary Table S5. Tentative names of EDC genes of *Rhineura floridana*

Gene name abbreviation	Full gene name
CBP	Corneous Beta Protein
Crnn	Cornulin
EDCATM	Epidermal Differentiation protein containing the CAT Motif
EDCG	Epidermal Differentiation protein rich in Cysteine and Glycine repeats
EDCML	Epidermal Differentiation protein containing a CCCC Motif Like
EDCRP	Epidermal Differentiation Cysteine-Rich Protein
EDCS	Epidermal Differentiation protein, Cysteine-rich Short
EDEPK	Epidermal Differentiation protein rich in glutamic acid (E), Proline and lysine (K)
EDEPT	Epidermal Differentiation protein rich in glutamic acid (E), Proline and Threonine
EDETM	Epidermal Differentiation protein containing an ET Motif
EDM	Epidermal Differentiation protein without unique Motifs
EDGPC	Epidermal Differentiation protein rich in Glycine, Proline and Cysteine
EDGY	Epidermal Differentiation protein rich in Glycine and tyrosine (Y)
EDH	Epidermal Differentiation protein rich in Histidine
EDHEM	Epidermal Differentiation protein containing a HEM Motif
EDKM	Epidermal Differentiation protein containing a KKLIQQ Motif
EDMGC	Epidermal Differentiation protein with a MGC start
EDP	Epidermal Differentiation protein rich in Proline
EDPCCC	Epidermal Differentiation protein containing PCCC repeats
EDPCS	Epidermal Differentiation protein rich in Proline, Cysteine and Serine
EDPL	Epidermal Differentiation Proline-rich protein, close to Loricrin
EDPKC	Epidermal Differentiation protein rich in Proline, lysine (K) and Cysteine
EDPQ	Epidermal Differentiation protein rich in Proline and glutamine (Q)
EDPSQ	Epidermal Differentiation protein rich in Proline, Serine and glutamine (Q)
EDQK	Epidermal Differentiation protein containing glutamine (Q) and lysine (K) repeats
EDQL	Epidermal Differentiation protein rich in glutamine (Q), close to Loricrin
EDSC	Epidermal Differentiation protein rich in Serine and Cysteine
EDSCP	Epidermal Differentiation protein rich in Serine, Cysteine and Proline
EDSPR1	Epidermal Differentiation protein Small Proline Rich 1
EDSQ	Epidermal Differentiation protein rich in Serine and glutamine (Q)
EDSRWM	Epidermal Differentiation protein containing a SRW Motif
EDWM	Epidermal Differentiation protein containing a WYDP Motif
EDYM	Epidermal Differentiation protein containing Y Motif
Lor	Loricrin
Pglyrp3	Peptidoglycan recognition protein 3
Scfn	Scaffoldin

Note - EDC genes encoding S100A proteins are not included here.

Supplementary Table S6. Locations of EDC genes of *Rhineura floridana*

Gene	Accession nr.	CDS start	CDS end	Expression confirmed by RNA-seq data *
S100-A12	NC_084501.1	16351028	16349933	yes
PGLYRP3	NC_084501.1	16333686	16329704	yes
EDKM	NC_084501.1	16321698	16319042	yes
EDM22	NC_084501.1	16313113	16313346	yes
EDM21	NC_084501.1	16306661	16306428	yes
EDPQ3	NC_084501.1	16300997	16301200	yes
EDH	NC_084501.1	16279974	16280366	yes
EDM20	NC_084501.1	16266547	16266876	yes
EDM19	NC_084501.1	16255945	16255655	yes
EDM18	NC_084501.1	16247124	16247414	yes
EDM17	NC_084501.1	16238321	16238103	yes
EDM16	NC_084501.1	16222846	16222628	yes
EDM15	NC_084501.1	16211595	16211335	no
EDM14	NC_084501.1	16170672	16170412	no
EDM13	NC_084501.1	16165157	16165411	yes
EDM12	NC_084501.1	16157026	16156766	yes
EDM11	NC_084501.1	16151511	16151765	yes
EDM10	NC_084501.1	16143380	16143120	no
EDM9	NC_084501.1	16137853	16138107	yes
EDM8	NC_084501.1	16128949	16128695	yes
EDM7	NC_084501.1	16111294	16111064	yes
EDM6	NC_084501.1	16102674	16102444	yes
EDM5	NC_084501.1	16092403	16092173	yes
EDM4	NC_084501.1	16082846	16082616	yes
EDM3	NC_084501.1	16072237	16072007	yes
EDM2	NC_084501.1	16056000	16055797	yes
EDM1	NC_084501.1	16046564	16046343	yes
EDSC3	NC_084501.1	16027508	16027801	yes
EDSC2	NC_084501.1	16010370	16010663	yes
EDSC1	NC_084501.1	16005843	16005550	yes
EDWM	NC_084501.1	15990310	15991002	yes
EDPQ1	NC_084501.1	15979526	15980398	yes
EDCS1	NC_084501.1	15961518	15961246	yes
COL1A1	NC_084501.1	15947051	15953500	yes
EDCS2L5	NC_084501.1	15933512	15933763	yes
EDCS3_3	NC_084501.1	15926524	15926174	yes
EDCS3_2	NC_084501.1	15922473	15922108	yes
EDCS3_1	NC_084501.1	15913916	15913536	yes
EDCS2L_4	NC_084501.1	15910772	15910521	yes
EDCS2L_3	NC_084501.1	15904504	15904253	yes
EDCS2L_2	NC_084501.1	15894863	15894600	yes
EDCS2L_1	NC_084501.1	15886462	15886190	yes
EDCS4_3	NC_084501.1	15865367	15865912	yes
EDCS4_2	NC_084501.1	15859209	15859640	yes
EDCS4_1	NC_084501.1	15852574	15853173	yes
EDCML	NC_084501.1	15848210	15848037	yes
EDCS3L_8	NC_084501.1	15842225	15842836	yes
EDCS3L_7	NC_084501.1	15830522	15829947	yes

Supplementary Table S6. Worm lizard (*Rhineura floridana*) EDC genes (continued)

Gene		CDS start	CDS end	Expression confirmed by RNA-seq data *
EDCS5_3	NC_084501.1	15826824	15826378	no
EDCS3L_6	NC_084501.1	15824148	15825068	yes
EDCS3L_5	NC_084501.1	15819479	15818685	yes
EDCS5_2	NC_084501.1	15815563	15815117	no
EDCS3L_4	NC_084501.1	15812659	15813807	yes
EDCS3L_3	NC_084501.1	15807995	15807276	yes
EDCS5_1	NC_084501.1	15804154	15803732	no
EDCS3L_2	NC_084501.1	15801514	15802422	yes
EDCS3L_1	NC_084501.1	15797077	15796094	yes
EDPCCC10	NC_084501.1	15787846	15788085	yes
EDPCCC9	NC_084501.1	15781609	15781830	yes
EDPCCC8	NC_084501.1	15775018	15775371	yes
EDPCCC7	NC_084501.1	15767481	15767702	yes
EDPCCC6	NC_084501.1	15760638	15760895	yes
EDPCCC5	NC_084501.1	15753100	15753321	yes
EDPCCC4	NC_084501.1	15746280	15746537	yes
EDPCCC3	NC_084501.1	15737597	15737854	yes
EDPCCC2	NC_084501.1	15730651	15730908	yes
EDPCCC1	NC_084501.1	15655785	15656024	yes
EDCRP4	NC_084501.1	15647655	15650639	yes
EDCRP3	NC_084501.1	15636698	15638629	yes
EDCRP2	NC_084501.1	15627467	15629638	yes
EDCRP1	NC_084501.1	15620276	15617064	yes
EDCG	NC_084501.1	15612280	15612507	yes
EDGPC	NC_084501.1	15583395	15583165	yes
EDMGC	NC_084501.1	15574429	15574653	yes
EDQL5	NC_084501.1	15560806	15561168	yes
EDQL4	NC_084501.1	15551763	15552044	yes
EDQL3	NC_084501.1	15545620	15545303	yes
EDQL2	NC_084501.1	15538889	15539188	yes
EDQL1	NC_084501.1	15533682	15533401	yes
LOR3	NC_084501.1	15513993	15513118	yes
LOR2	NC_084501.1	15507473	15508336	yes
LOR1	NC_084501.1	15494902	15496422	yes
EDPL	NC_084501.1	15482282	15482449	no
CBP1	NC_084501.1	15453773	15454897	yes
EDSRWM	NC_084501.1	15198049	15199164	yes
EDGY1	NC_084501.1	15176386	15176775	yes
EDGY2	NC_084501.1	15156184	15155507	yes
EDETM	NC_084501.1	14685640	14685380	yes
EDSCP6	NC_084501.1	14457345	14456263	yes
EDSCP5	NC_084501.1	14442659	14441577	yes
EDSCP4	NC_084501.1	14430325	14431095	yes
EDSCP3	NC_084501.1	14422058	14421405	yes
EDSCP2	NC_084501.1	14412584	14413252	yes

Supplementary Table S6. Worm lizard (*Rhineura floridana*) EDC genes (continued)

Gene		CDS start	CDS end	Expression confirmed by RNA-seq data *
EDSCP1	NC_084501.1	14396962	14395967	yes
CBP_last	NC_084501.1	14295773	14295252	yes
EDYM2	NC_084501.1	14272392	14275089	yes
EDPSQ	NC_084501.1	14255407	14256768	yes
EDEPK	NC_084501.1	14234598	14235083	no
EDPKC	NC_084501.1	14208863	14207586	yes
EDP3	NC_084501.1	14201293	14201517	yes
EDSPRL1	NC_084501.1	14186238	14186654	yes
EDQK	NC_084501.1	14167610	14167831	yes
EDSPR1	NC_084501.1	14159932	14160231	no
EDSQ	NC_084501.1	14127510	14126389	yes
EDEPT	NC_084501.1	14117427	14116567	yes
EDSPRL2	NC_084501.1	14104724	14104879	yes
CRNN	NC_084501.1	14081102	14077486	no
SCFN2	NC_084501.1	14063955	14066734	no
SCFN1	NC_084501.1	14045422	14040540	no
S100-A11	NC_084501.1	14033751	14027723	yes
S100A-10	NC_084501.1	13997454	13995163	yes
THEM4-like	NC_084501.1	13982100	13974841	yes
THEM4-like	NC_084501.1	13955001	13939567	yes
SCFN3	NC_084501.1	13934307	13936892	no

Notes - * "RNA-seq evidence" corresponds to the presence of RNA-seq peaks in the "Genomic regions, transcripts and products" view at the NCBI GenBank website (www.ncbi.nlm.nih.gov, accessed on 5 June 2024). Only the S100A genes flanking PGLYRP3 and SCFN1 are shown here. CDS, coding sequence.

Suppl. Table S7. Proteomic analysis of clawed toes of *A. carolinensis*

Accession (Uniprot) or new annotation*	Gene Symbol	Description	Notes	Sum PEP Score	Score Sequest HT: Sequest HT	Exp. q-value: Combined	Coverage [%]	# Peptides	# PSMs	# Unique Peptides	# AAs	MW [kDa]	calc. pI
R4G9N5	LOC100553064	IF rod domain-containing protein OS=Anolis carolinensis OX=28377 GN=LOC100553064 PE=4 SV=1	keratin, type I cytoskeletal 10	605,7	538,9	0	73	49	230	48	657	66,3	5,82
KRT78L2	LOC100560037	KRT78L2	KRT78L2	492,5	399,8	0	54	56	205	53	652	66,5	8,21
G1KGX8	LOC100566088	IF rod domain-containing protein OS=Anolis carolinensis OX=28377 GN=LOC100566088 PE=3 SV=1	keratin, type II cytoskeletal 5	378,3	317,4	0	63	54	169	42	632	65,1	7,03
KRT5L4*		KRT5L4	KRT5L4	376,3	266,9	0	83	50	129	33	524	56,7	8,05
KRT78L4*	LOC100560037	KRT78L4	KRT78L4	338,9	210,4	0	67	43	86	33	628	64,1	8,32
R4GAI6	KRT15	Keratin 15 OS=Anolis carolinensis OX=28377 GN=KRT15 PE=3 SV=2	KRT15	282,7	215,3	0	63	40	114	31	505	53,3	5,08
G1KUG5	LOC100564200	IF rod domain-containing protein OS=Anolis carolinensis OX=28377 GN=LOC100564200 PE=3 SV=2	keratin, type I cytoskeletal 14	265,3	211,7	0	73	34	113	24	429	47,2	5,05
G1KS08	KRT24L	KRT24L (Ehrlich et al. 2020)	KRT24L	254,8	180,9	0	68	35	87	35	504	53,1	5,19
KRT14L2*	LOC100564200	KRT14L2	KRT14L2	246,8	136,2	0	67	32	73	26	455	50,2	5,35
KRT84L1*		KRT84L1 (Ehrlich et al. 2020) = HB4 (Eckhart et al. 2008)	**KRT84L1/HB4 -hair keratin homolog type II**	239,1	142,4	0	78	43	71	42	542	60,5	7,15
KRT36L2*	KRT36L2/HA2	KRT36L2 (Ehrlich et al. 2020) = HA2 (Eckhart et al. 2008)	**KRT36L2/HA2 - hair keratin homolog type I**	220,5	132,3	0	60	31	66	30	446	50,5	5,49
B6Z1W5	LOC100337545	Keratin aHB1 OS=Anolis carolinensis OX=28377 GN=LOC100337545 PE=2 SV=1	**KRT84L4/HB1 -hair keratin homolog type II**	190,6	133,7	0	75	39	62	30	552	61	7,93
B6Z1W2	KRT36L1/HA1	Keratin 36 OS=Anolis carolinensis OX=28377 GN=KRT36 PE=2 SV=1 = HA1 (Eckhart et al. 2008)	**KRT36L1/HA1 - hair keratin homolog type I**	158,3	85,0	0	61	28	38	28	525	58,2	5,06
KRT84L2*		KRT84L2 (Ehrlich et al. 2020) = HB2 (Eckhart et al. 2008)	**KRT84L2/HB2 -hair keratin homolog type II**	158,2	84,1	0	47	27	42	23	555	61,7	6,9
KRT9L2*		KRT9L2	KRT9L2	141,0	70,8	0	38	19	29	1	828	81,9	6,81
G1KWV8		IF rod domain-containing protein OS=Anolis carolinensis OX=28377 PE=4 SV=2	keratin, type I cytoskeletal 10 X1	133,3	67,1	0	51	18	24	1	594	61,3	6,21
KRT84L3*		KRT84L3 (Ehrlich et al. 2020) = HB3 (Eckhart et al. 2008)	**KRT84L3/HB3 -hair keratin homolog type II**	124,2	70,6	0	45	23	29	23	521	57,7	6,73
R4G9U3	LOC100552076	IF rod domain-containing protein OS=Anolis carolinensis OX=28377 GN=LOC100552076 PE=3 SV=1	keratin, type I cytoskeletal 24	121,3	59,6	0	49	22	47	21	497	53,8	5,25
R4GB02		Keratin, type II cytoskeletal 5 OS=Anolis carolinensis OX=28377 PE=3 SV=1	keratin, type II cytoskeletal 5	109,4	58,5	0	41	26	36	20	646	66,6	7,5
G1KH77		IF rod domain-containing protein OS=Anolis carolinensis OX=28377 PE=3 SV=1	keratin, type II cytoskeletal 6A	89,6	62,2	0	31	20	28	7	576	61,2	7,61
H9GI12	krt8	Keratin, type II cytoskeletal 8 OS=Anolis carolinensis OX=28377 GN=krt8 PE=3 SV=2	keratin, type II cytoskeletal 8	69,7	50,6	0	29	15	27	9	483	53,3	5,5
G1KVF3		Keratin, type II cytoskeletal 5 OS=Anolis carolinensis OX=28377 PE=3 SV=2	keratin, type II cytoskeletal 5	58,2	44,7	0	18	13	28	2	507	53,9	5,49
KRT80*	LOC100562605	KRT80	KRT80	39,8	13,7	0	15	11	12	11	1324	143,6	6,25
G1KHP6	LOC100567055	IF rod domain-containing protein OS=Anolis carolinensis OX=28377 GN=LOC100567055 PE=3 SV=2	keratin, type II cytoskeletal cochleal	39,3	27,6	0	14	10	21	1	522	56,6	6,04
H9GI59	krt18	IF rod domain-containing protein OS=Anolis carolinensis OX=28377 GN=krt18 PE=3 SV=2	Krt18	34,8	18,4	0	26	9	21	7	441	49,2	5,5
KRT78L1*		KRT78L1	KRT78L1	27,3	22,3	0	10	8	15	1	545	59	7,18
A0A803TKX0	LOC100563808	IF rod domain-containing protein OS=Anolis carolinensis OX=28377 GN=LOC100563808 PE=3 SV=1	keratin, type I cytoskeletal 19	26,8	24,8	0	15	8	14	4	436	47,2	5,06
R4GBD3	KRT23	Keratin 23 OS=Anolis carolinensis OX=28377 GN=KRT23 PE=3 SV=1	krt23	11,8	2,2	0	7	3	4	3	432	48,8	5,55
HBS2*		HBS2	HBS2 keratin	10,9	4,4	0	6	4	4	3	754	78,5	7,69
A0A803T7J2	LOC100553851	IF rod domain-containing protein OS=Anolis carolinensis OX=28377 GN=LOC100553851 PE=3 SV=1	keratin, type I cytoskeletal 19	5,1	3,7	0	3	2	4	1	619	65	6,2
A0A803TVC5		Keratin OS=Anolis carolinensis OX=28377 PE=4 SV=1	CBP, Li-Ac-37	290,8	175,3	0	64	32	75	32	492	43,3	10,4
EDGY2*		EDGY2	EDGY2	149,5	72,4	0	69	18	34	18	324	33,5	8,85
LOR2*		LOR2	LOR2	52,2	20,3	0	27	7	10	7	543	46,8	9,03
EDCC1*		EDCC1	**EDCC1**	50,9	45,5	0	22	2	21	2	140	13,6	5,22
EDSCP*		EDSCP	EDSCP	29,1	14,7	0	9	2	3	2	232	24,5	7,49
A0A088BHB3	EDCM	Epidermal differentiation protein OS=Anolis carolinensis OX=28377 GN=EDCM PE=2 SV=1	EDCM	27,3	13,5	0	67	5	6	5	72	8,1	9,06
EDCC2*		EDCC2	**EDCC2**	26,6	18,3	0	13	1	7	1	149	14,7	6,07
A0A803TK53	LOC107983365	Epidermal differentiation protein OS=Anolis carolinensis OX=28377 GN=LOC107983365 PE=4 SV=1	EDWM	25,2	13,3	0	27	7	7	7	329	34,5	8,4
EDGGY2*		EDGGY2	EDGGY2	24,6	9,7	0	63	2	4	2	94	9,6	8,16
EDGGY1*		EDGGY1	EDGGY1	19,9	16,0	0	61	2	6	2	67	7,3	7,78
H9GQG3		Keratin OS=Anolis carolinensis OX=28377 PE=3 SV=2	CBP	17,2	7,2	0	11	1	3	1	170	16,1	8,22
A0A803T8V4	LOC100558171	Keratin OS=Anolis carolinensis OX=28377 GN=LOC100558171 PE=3 SV=1	CBP	10,7	3,5	0	12	1	1	1	217	21,6	8,5
EDPCCC3 partial		EDPCCC3 partial	EDPCCC3	9,2	2,1	0	38	3	3	3	216	22,4	8,1
H9GCU0	LOC100557315	Keratin OS=Anolis carolinensis OX=28377 GN=LOC100557315 PE=3 SV=2	CBP	8,6	5,2	0	14	2	2	2	184	17,9	5,25
EDGY1*		EDGY1	EDGY1	7,6	4,3	0	17	2	3	2	123	13,7	8,6
H9GCU2	LOC100558368	Keratin OS=Anolis carolinensis OX=28377 GN=LOC100558368 PE=3 SV=2	CBP	7,2	2,4	0	20	3	4	3	177	17,1	7,61
EDPCCC4*		EDPCCC4	EDPCCC4	6,1	3,2	0	15	1	1	1	100	10	7,05
EDCPGS*		EDCPGS	EDCPGS	5,4	0,0	0	11	2	2	2	267	25,7	7,78
A0A803U1H9	li-ac-35	Keratin OS=Anolis carolinensis OX=28377 GN=li-ac-35 PE=4 SV=1	CBP, li-ac-35	4,6	2,4	0	7	1	1	1	136	14,9	9,77
H9GRP9		Keratin OS=Anolis carolinensis OX=28377 PE=3 SV=1	CBP	4,5	2,8	0	11	1	1	1	119	11,5	7,47
A0A088BIC4	EDSPR1	Epidermal differentiation protein OS=Anolis carolinensis OX=28377 GN=EDSPR1 PE=2 SV=1	EDSPR1	3,6	0,0	0	10	1	1	1	125	12,5	8,31
EDCS2*		EDCS2	EDCS2	3,0	0,0	0,001	10	1	1	1	102	10,5	8,02
G1KUH3		Junction plakoglobin OS=Anolis carolinensis OX=28377 PE=3 SV=2		240,8	141,0	0	57	30	58	28	743	81,3	6,1
R4GBL1	PKP1	Plakophilin 1 OS=Anolis carolinensis OX=28377 GN=PKP1 PE=3 SV=1		239,5	126,3	0	65	35	53	35	729	81,3	9,14
H9GPT2	DSG1	Desmoglein 4 OS=Anolis carolinensis OX=28377 GN=DSG1 PE=4 SV=2		200,0	108,5	0	31	25	40	25	1247	130,3	4,73
G1KYJ6		Desmoplakin SH3 domain-containing protein OS=Anolis carolinensis OX=28377 PE=4 SV=3		129,4	44,1	0	21	35	37	35	2001	232	7,15
G1KNU2	PKP3	Plakophilin 3 OS=Anolis carolinensis OX=28377 GN=PKP3 PE=3 SV=3		67,4	31,7	0	28	18	24	18	817	91,4	9,35
H9GPT4	DSG2	Desmoglein 2 OS=Anolis carolinensis OX=28377 GN=DSG2 PE=4 SV=2		29,5	11,9	0	12	8	8	8	1102	119	4,97
H9GED3	PLEC	SH3 domain-containing protein OS=Anolis carolinensis OX=28377 PE=4 SV=2		953,8	511,2	0	47	201	248	197	4393	501,2	6,24
H9G6P9		SH3 domain-containing protein OS=Anolis carolinensis OX=28377 PE=4 SV=2		942,5	526,7	0	59	177	234	177	2879	332,1	6,79
A0A803THA1		AHNAK nucleoprotein OS=Anolis carolinensis OX=28377 PE=4 SV=1		899,7	418,2	0	60	184	221	184	5447	582,5	6,18
A0A803TTS7	COL6A3	Collagen type VI alpha 3 chain OS=Anolis carolinensis OX=28377 GN=COL6A3 PE=4 SV=1		870,0	491,1	0	50	123	212	123	3094	335,1	6,48
A0A803T1M9	COL12A1	Collagen type XII alpha 1 chain OS=Anolis carolinensis OX=28377 GN=COL12A1 PE=4 SV=1		841,1	440,5	0	49	124	189	124	3118	338,6	5,45
G1KSP5	myh9	Myosin-9 OS=Anolis carolinensis OX=28377 GN=myh9 PE=3 SV=1		520,5	275,2	0	45	80	100	65	1960	226,8	5,63
H9GH08	LOC100562375	Albumin domain-containing protein OS=Anolis carolinensis OX=28377 GN=LOC100562375 PE=4 SV=2		483,3	303,7	0	81	54	134	1	612	69,2	6,07
A0A803TJY3	LOC100562375	Albumin domain-containing protein OS=Anolis carolinensis OX=28377 GN=LOC100562375 PE=4 SV=1		478,5	299,0	0	78	54	132	1	633	71,5	6,07
G1KKJ8	apob	Apolipoprotein B OS=Anolis carolinensis OX=28377 GN=apob PE=4 SV=2		464,1	204,1	0	31	100	115	100	4522	510,2	6,71
G1KJT9	flna	Calponin-homology (CH) domain-containing protein OS=Anolis carolinensis OX=28377 GN=flna PE=3 SV=3		416,4	190,1	0	41	75	94	68	2710	287,6	6,2
G1KM40	SPTBN1	Spectrin beta chain OS=Anolis carolinensis OX=28377 GN=SPTBN1 PE=3 SV=3		397,8	187,0	0	43	77	90	1	2345	271,8	5,59
A0A803TN83	SPTBN1	Spectrin beta chain OS=Anolis carolinensis OX=28377 GN=SPTBN1 PE=3 SV=1		395,1	185,1	0	43	77	90	1	2361	273,9	5,54
G1KRT6	FN1	Fibronectin OS=Anolis carolinensis OX=28377 GN=FN1 PE=4 SV=3		380,9	169,2	0	38	57	79	57	2476	273,2	5,71
H9G3B2	DYNC1H1	Dynein cytoplasmic 1 heavy chain 1 OS=Anolis carolinensis OX=28377 GN=DYNC1H1 PE=3 SV=2		347,9	151,5	0	24	88	95	88	4682	536,3	6,43
G1KDR6	VIM	Vimentin OS=Anolis carolinensis OX=28377 GN=VIM PE=3 SV=3		347,7	210,9	0	74	43	91	39	465	53,2	5,25
A0A803TH08	dst	SH3 domain-containing protein OS=Anolis carolinensis OX=28377 GN=dst PE=4 SV=1		345,1	166,1	0	33	74	85	73	2621	303,2	6,24
A0A803TW09	LOC100554738	Transferrin-like domain-containing protein OS=Anolis carolinensis OX=28377 GN=LOC100554738 PE=3 SV=1		336,8	186,5	0	73	45	69	45	707	76,3	6,15
G1KQS3	c3	Complement C3 OS=Anolis carolinensis OX=28377 GN=c3 PE=4 SV=3		318,2	167,5	0	39	57	74	43	1660	186,8	6,4
A0A803TVW9	actn4	Alpha-actinin-4 OS=Anolis carolinensis OX=28377 GN=actn4 PE=3 SV=1		308,5	166,4	0	62	49	72	1	927	106,6	5,3
A0A803TY64	actn4	Alpha-actinin-4 OS=Anolis carolinensis OX=28377 GN=actn4 PE=3 SV=1		304,2	167,3	0	61	49	72	2	905	104,1	5,3
A0A803T3H5		Lamin A/C OS=Anolis carolinensis OX=28377 PE=3 SV=1		291,8	188,4	0	61	42	76	40	609	67,4	8,32
A0A803T1B7		EF-hand domain-containing protein OS=Anolis carolinensis OX=28377 PE=4 SV=1		289,1	130,6	0	44	53	65	3	1298	149,4	5,58
H9G7W7		IQ motif containing GTPase activating protein 1 OS=Anolis carolinensis OX=28377 PE=4 SV=2		281,1	143,1	0	33	52	62	50	1922	217,4	7,42
A0A803TVY1	POSTN	Periostin OS=Anolis carolinensis OX=28377 GN=POSTN PE=4 SV=1		277,3	131,6	0	59	37	51	4	836	93,2	7,31
A0A803SYR2	POSTN	Periostin OS=Anolis carolinensis OX=28377 GN=POSTN PE=4 SV=1		272,1	130,1	0	57	37	51	4	871	97,2	7,56
A0A803STS1		EF-hand domain-containing protein OS=Anolis carolinensis OX=28377 PE=4 SV=1		269,5	118,7	0	46	51	61	1	1196	137,5	5,49
H9GBH0	PPL	Periplakin OS=Anolis carolinensis OX=28377 GN=PPL PE=4 SV=2		243,7	120,0	0	38	56	67	56	1762	206	5,47
H9GHJ1	TLN1	Talin 1 OS=Anolis carolinensis OX=28377 GN=TLN1 PE=4 SV=3		243,5	102,3	0	33	51	59	47	2292	244,7	6,39
G1KD78	COL6A2	Collagen type VI alpha 2 chain OS=Anolis carolinensis OX=28377 GN=COL6A2 PE=4 SV=2		227,3	118,0	0	27	33	53	12	1019	108,4	6,04
A0A803TZC8		Moesin OS=Anolis carolinensis OX=28377 GN=MSN PE=4 SV=1		226,2	112,5	0	53	37	53	28	612	71,8	6,58
G1KDW3	COL1A1	Alpha-1 type I collagen OS=Anolis carolinensis OX=28377 GN=COL1A1 PE=4 SV=3		225,6	201,9	0	28	30	129	20	1450	137,5	5,73
H9GPS3	LOC100565442	Cadherin domain-containing protein OS=Anolis carolinensis OX=28377 GN=LOC100565442 PE=4 SV=2		223,7	106,6	0	57	35	55	35	855	94,4	5,71
H9GMQ9	ANXA2	Annexin OS=Anolis carolinensis OX=28377 GN=ANXA2 PE=3 SV=2		220,4	122,3	0	55	26	45	26	406	45	8,21
A0A803TQG2	FLNB	Filamin B OS=Anolis carolinensis OX=28377 GN=FLNB PE=3 SV=1		215,7	102,9	0	25	44	52	38	2634	281,8	5,49
H9GAA9	ACTN1	Alpha-actinin-1 OS=Anolis carolinensis OX=28377 GN=ACTN1 PE=3 SV=2		210,1	110,1	0	50	37	50	23	893	103,1	5,35
A0A803SLL7	myh11	Myosin heavy chain 14 OS=Anolis carolinensis OX=28377 GN=myh11 PE=3 SV=1		203,6	92,3	0	24	37	48	28	1990	230,2	5,72
A0A803SPD9	VCL	Vinculin OS=Anolis carolinensis OX=28377 GN=VCL PE=3 SV=1		201,5	115,3	0	39	39	49	39	1066	116,8	6,18
A0A803T2G8	CLTC	Clathrin heavy chain OS=Anolis carolinensis OX=28377 GN=CLTC PE=3 SV=1		199,8	90,2	0	30	37	43	23	1694	193,5	5,74
G1KQE0	HSP90AB1	Heat shock protein 90 alpha family class B member 1 OS=Anolis carolinensis OX=28377 GN=HSP90AB1 PE=3 SV=2		197,5	94,1	0	49	33	44	27	727	83,4	5,06
A0A803TZD8		Complement C3 OS=Anolis carolinensis OX=28377 PE=4 SV=1		196,5	81,1	0	39	45	51	2	1512	170,3	7,53
A0A803TES0		Methanethiol oxidase OS=Anolis carolinensis OX=28377 PE=3 SV=1		194,8	104,0	0	73	20	44	13	422	47,3	5,73
G1KQX8	LOC100557873	Alpha-2-macroglobulin-like protein 1 OS=Anolis carolinensis OX=28377 GN=LOC100557873 PE=3 SV=3		190,7	96,1	0	32	36	47	35	1442	162,2	5,11
H9GTP6		Uncharacterized protein OS=Anolis carolinensis OX=28377 PE=4 SV=2		187,6	71,6	0	15	47	52	45	5755	647,5	5,52
H9GMK9	LOC100562036	Tubulin alpha chain OS=Anolis carolinensis OX=28377 GN=LOC100562036 PE=3 SV=3		187,6	115,3	0	72	23	42	2	449	49,9	5,1
A0A803T2N4		Alpha-macroglobulin receptor-binding domain-containing protein OS=Anolis carolinensis OX=28377 PE=4 SV=1		186,1	76,2	0	44	22	43	3	543	60,1	6,43
A0A803TS16		Fibronectin type-III domain-containing protein OS=Anolis carolinensis OX=28377 PE=4 SV=1		184,7	103,2	0	36	42	59	18	1352	146,3	9,03
A0A803TXT4		Complement C3 OS=Anolis carolinensis OX=28377 PE=4 SV=1		184,6	76,1	0	36	41	48	1	1510	170,8	7,39
H9GI83	VCP	Transitional endoplasmic reticulum ATPase OS=Anolis carolinensis OX=28377 GN=VCP PE=3 SV=2		183,9	103,0	0	54	38	54	27	807	89,4	5,3
R4GAT8	MATN1	Matrilin 1 OS=Anolis carolinensis OX=28377 GN=MATN1 PE=4 SV=2		176,7	127,5	0	54	30	57	30	572	62,6	7,53
A0A803TA36		Alpha-macroglobulin receptor-binding domain-containing protein OS=Anolis carolinensis OX=28377 PE=4 SV=1		175,3	76,5	0	47	20	41	1	473	52,8	6,48
L7MZZ9	tubb4b	Tubulin beta chain OS=Anolis carolinensis OX=28377 GN=tubb4b PE=3 SV=1		175,0	92,6	0	63	22	31	3	445	49,8	4,89
G1KNN1	LOC100561212	Tubulin alpha chain OS=Anolis carolinensis OX=28377 GN=LOC100561212 PE=3 SV=2		172,7	101,7	0	69	21	37	6	448	49,9	5,02
G1KT22	ATP5F1B	ATP synthase subunit beta OS=Anolis carolinensis OX=28377 GN=ATP5F1B PE=3 SV=2		171,7	83,2	0	57	19	33	19	528	56,5	5,27
G1KNZ5	COL1A2	Collagen type I alpha 2 chain OS=Anolis carolinensis OX=28377 GN=COL1A2 PE=4 SV=2		170,5	146,9	0	24	25	99	23	1354	128,6	9,16
A0A803TE46		Anaphylatoxin-like domain-containing protein OS=Anolis carolinensis OX=28377 PE=4 SV=1		168,1	69,7	0	35	41	46	1	1487	167,3	7,05
G1KE14	UBA1	E1 ubiquitin-activating enzyme OS=Anolis carolinensis OX=28377 GN=UBA1 PE=3 SV=1		167,2	82,5	0	38	28	39	28	1059	118,9	5,64
G1K957	LAMA3	Laminin subunit alpha 3 OS=Anolis carolinensis OX=28377 GN=LAMA3 PE=4 SV=3		163,5	70,4	0	15	39	47	5	3214	357,1	6,44
A0A803TEU5	LAMA3	Laminin subunit alpha 3 OS=Anolis carolinensis OX=28377 GN=LAMA3 PE=4 SV=1		163,2	74,6	0	15	37	44	1	3085	342,6	6,6
G1KRK2	HSPA2	Heat shock protein family A (Hsp70) member 2 OS=Anolis carolinensis OX=28377 GN=HSPA2 PE=3 SV=1		163,0	76,7	0	43	26	41	18	635	69,8	5,76
A0A803T951	FBN1	Fibrillin 1 OS=Anolis carolinensis OX=28377 GN=FBN1 PE=4 SV=1		162,1	77,5	0	15	32	42	31	2202	239,9	4,78
H9GBL1	GAPDH	Glyceraldehyde-3-phosphate dehydrogenase OS=Anolis carolinensis OX=28377 GN=GAPDH PE=3 SV=1		158,6	75,8	0	51	14	29	14	333	35,7	8,27
A0A803TLZ0	LOC100567488	Beta-actin OS=Anolis carolinensis OX=28377 GN=LOC100567488 PE=3 SV=1		157,8	95,3	0	55	16	52	1	376	41,8	5,48
G1KB81	LMNB1	Lamin B1 OS=Anolis carolinensis OX=28377 GN=LMNB1 PE=3 SV=2		156,4	81,6	0	50	26	36	24	591	66,9	5,15
L7MZT7	LOC100553546	Methanethiol oxidase OS=Anolis carolinensis OX=28377 GN=LOC100553546 PE=3 SV=2		151,1	87,5	0	75	29	35	20	454	50,9	5,6
G1KDJ9	LOC100562152	Tubulin alpha chain OS=Anolis carolinensis OX=28377 GN=LOC100562152 PE=3 SV=2		150,8	91,5	0	59	22	36	9	449	49,8	5,14
G1KH79		Inter-alpha-trypsin inhibitor heavy chain H3 OS=Anolis carolinensis OX=28377 PE=3 SV=3		150,0	82,4	0	29	23	40	18	900	101,5	6,16
H9G9N5	HSPA8	Heat shock protein family A (Hsp70) member 8 OS=Anolis carolinensis OX=28377 GN=HSPA8 PE=3 SV=1		148,1	72,9	0	45	23	35	10	646	70,8	5,52
G1KMS6	tktl1	transketolase OS=Anolis carolinensis OX=28377 GN=tktl1 PE=3 SV=1		148,0	65,7	0	59	24	27	23	626	67,8	7,64
G1KY87	PAPLN	Papilin, proteoglycan like sulfated glycoprotein OS=Anolis carolinensis OX=28377 GN=PAPLN PE=4 SV=2		147,9	59,1	0	31	28	34	27	1333	146,6	7,2
H9GLK7	glud1	Glutamate dehydrogenase 1, mitochondrial OS=Anolis carolinensis OX=28377 GN=glud1 PE=3 SV=1		143,5	65,5	0	50	23	32	11	549	60,5	8,05
A0A803T154	PLS3	Plastin-3 OS=Anolis carolinensis OX=28377 GN=PLS3 PE=4 SV=1		143,5	78,4	0	57	28	35	3	625	70,2	6
G1KPP3		Fibronectin type-III domain-containing protein OS=Anolis carolinensis OX=28377 PE=4 SV=3		143,3	76,3	0	38	36	46	25	1246	136,3	5,59
A0A803TJZ0	LAMB2	Laminin subunit beta 2 OS=Anolis carolinensis OX=28377 GN=LAMB2 PE=4 SV=1		141,5	58,4	0	21	29	31	27	1711	189	7,46
H9GD14	sptbn2	Spectrin beta chain OS=Anolis carolinensis OX=28377 GN=sptbn2 PE=3 SV=2		140,4	67,2	0	19	32	36	28	2278	261,3	5,64
R4GCN4	LOC100554364	Heat shock protein family A (Hsp70) member 8 OS=Anolis carolinensis OX=28377 GN=LOC100554364 PE=3 SV=2		138,3	70,0	0	47	24	31	10	652	71,3	5,57
A0A803STT9		Uncharacterized protein OS=Anolis carolinensis OX=28377 PE=3 SV=1		137,8	59,7	0	21	26	37	1	1404	157,4	7,2
G1KNV6	VAT1	Vesicle amine transport 1 OS=Anolis carolinensis OX=28377 GN=VAT1 PE=3 SV=3		136,6	81,1	0	60	16	26	16	410	43,8	6,13
A0A803T0Q2	COL6A2	Collagen type VI alpha 2 chain OS=Anolis carolinensis OX=28377 GN=COL6A2 PE=4 SV=1		135,0	72,8	0	21	23	38	2	915	96,8	5,94
A0A803SSK1	PLS3	Plastin-3 OS=Anolis carolinensis OX=28377 GN=PLS3 PE=4 SV=1		134,6	70,1	0	55	26	32	1	607	68	6,29
A0A803U0S7		Tenascin C OS=Anolis carolinensis OX=28377 PE=4 SV=1		134,5	63,4	0	27	22	30	22	1288	142,7	5,24
A0A803T9A6		Inter-alpha-trypsin inhibitor heavy chain H3 OS=Anolis carolinensis OX=28377 PE=3 SV=1		134,2	74,3	0	29	22	28	15	864	97,2	5,71
A0A803T871		Alpha-2-macroglobulin OS=Anolis carolinensis OX=28377 PE=3 SV=1		134,2	58,1	0	20	26	36	1	1477	165	6,61
A0A803SX86	ATP5F1A	ATP synthase subunit alpha OS=Anolis carolinensis OX=28377 GN=ATP5F1A PE=3 SV=1		132,8	83,0	0	50	23	32	23	560	60,5	9,13
A0A803TSR9		VWFA domain-containing protein OS=Anolis carolinensis OX=28377 PE=4 SV=1		130,5	61,2	0	37	26	32	1	896	98	6,67
R4GAH2	PKM	Pyruvate kinase OS=Anolis carolinensis OX=28377 GN=PKM PE=3 SV=2		130,0	74,0	0	57	22	30	2	527	57,6	7,31
L7MZS1		Methanethiol oxidase OS=Anolis carolinensis OX=28377 PE=3 SV=2		129,5	52,4	0	60	20	31	2	474	52,9	5,49
A0A803T123		VWFA domain-containing protein OS=Anolis carolinensis OX=28377 PE=4 SV=1		129,1	61,2	0	37	26	32	1	897	98,1	6,51
H9GL50		Methanethiol oxidase OS=Anolis carolinensis OX=28377 PE=3 SV=2		129,1	52,6	0	68	21	30	2	458	51	5,72
H9GQF6		Ig-like domain-containing protein OS=Anolis carolinensis OX=28377 PE=4 SV=3		129,0	68,3	0	52	19	31	19	484	53,7	6,16
H9G8M3	hspa5	78 kDa glucose-regulated protein OS=Anolis carolinensis OX=28377 GN=hspa5 PE=3 SV=2		128,9	68,9	0	37	21	29	19	655	72,3	5,16
G1KP96	LTA4H	Leukotriene A(4) hydrolase OS=Anolis carolinensis OX=28377 GN=LTA4H PE=3 SV=2		126,9	74,2	0	48	27	32	27	613	69,3	6,49
A0A803TU95	hnrnpa3	RRM domain-containing protein OS=Anolis carolinensis OX=28377 GN=hnrnpa3 PE=4 SV=1		126,5	52,2	0	39	16	21	16	362	37,5	8,4
A0A803T189	ITGB4	Integrin beta OS=Anolis carolinensis OX=28377 GN=ITGB4 PE=3 SV=1		126,3	53,7	0	22	27	30	27	1805	202,2	5,77
G1KNL0	GC	Vitamin D-binding protein OS=Anolis carolinensis OX=28377 GN=GC PE=4 SV=2		126,1	58,7	0	49	22	30	22	485	54,5	5,92
A0A803SWT7	MARS1	Methionine--tRNA ligase, cytoplasmic OS=Anolis carolinensis OX=28377 GN=MARS1 PE=3 SV=1		126,0	58,3	0	32	22	27	22	892	100,8	6,98
H9GGM8	LOC100556620	Globin domain-containing protein OS=Anolis carolinensis OX=28377 GN=LOC100556620 PE=3 SV=1		125,7	83,4	0	95	17	39	17	142	16	7,97
G1KM62	MATN4	Matrilin 4 OS=Anolis carolinensis OX=28377 GN=MATN4 PE=4 SV=2		125,4	72,9	0	38	21	30	21	622	69,4	7,05
H9GHQ2	PKM	Pyruvate kinase OS=Anolis carolinensis OX=28377 GN=PKM PE=3 SV=2		125,0	70,8	0	53	21	29	1	527	57,7	6,84
A0A803T638	EPRS1	Glutamyl-prolyl-tRNA synthetase 1 OS=Anolis carolinensis OX=28377 GN=EPRS1 PE=3 SV=1		122,9	43,4	0	24	27	29	27	1689	189,6	8,1

Suppl. Table S8. Proteomic analysis of back skin of *A. carolinensis*

Accession (Uniprot) or new annotation*	Gene Symbol	Description	Notes	Sum PEP Score	Score Sequest HT: Sequest HT	Exp. q-value: Combined	Coverage [%]	# Peptides	# PSMs	# Unique Peptides	# AAs	MW [kDa]	calc. pI
R4G9N5	LOC100553064	IF rod domain-containing protein OS=Anolis carolinensis OX=28377 GN=LOC100553064 PE=4 SV=1	keratin, type I cytoskeletal 10	810,4	637,2	0	74	60	243	60	657	66,3	5,82
KRT78L2*	LOC100560037	KRT78L2	KRT78L2	619,7	453,3	0	54	64	215	61	652	66,5	8,21
G1KS08	KRT24L	KRT24L (Ehrlich et al. 2020)	KRT24L	497,8	468,8	0	78	53	217	25	504	53,1	5,19
KRT78L4*	LOC100560037	KRT78L4	KRT78L4	482,3	364,9	0	70	53	131	45	628	64,1	8,32
G1KGX8	LOC100566088	IF rod domain-containing protein OS=Anolis carolinensis OX=28377 GN=LOC100566088 PE=3 SV=1	keratin, type II cytoskeletal 5	481,6	481,5	0	66	58	259	47	632	65,1	7,03
R4GAI6	KRT15	Keratin 15 OS=Anolis carolinensis OX=28377 GN=KRT15 PE=3 SV=2	KRT15	392,2	309,2	0	69	52	161	43	505	53,3	5,08
A0A803SQT9	krt36L2/HA2	Keratin 36 OS=Anolis carolinensis OX=28377 GN=krt36 PE=3 SV=1	**KRT36L2/HA2 - hair keratin homolog type I**	245,4	177,4	0	51	30	97	1	457	50,7	5,21
R4G9U3	LOC100552076	IF rod domain-containing protein OS=Anolis carolinensis OX=28377 GN=LOC100552076 PE=3 SV=1	keratin, type I cytoskeletal 24	243,0	151,8	0	68	40	86	38	497	53,8	5,25
H9GI59	krt18	IF rod domain-containing protein OS=Anolis carolinensis OX=28377 GN=krt18 PE=3 SV=2	KRT18	200,9	123,1	0	69	21	51	19	441	49,2	5,5
H9GI12	krt8	Keratin, type II cytoskeletal 8 OS=Anolis carolinensis OX=28377 GN=krt8 PE=3 SV=2	KRT8	197,0	109,7	0	65	31	44	26	483	53,3	5,5
KRT14L2*	LOC100564200	KRT14L2	KRT14L2	167,2	106,1	0	62	28	48	25	455	50,2	5,35
R4GB02		Keratin, type II cytoskeletal 5 OS=Anolis carolinensis OX=28377 PE=3 SV=1	keratin, type II cytoskeletal 4	121,2	68,5	0	47	26	33	20	646	66,6	7,5
A0A803SR03	LOC100564200	IF rod domain-containing protein OS=Anolis carolinensis OX=28377 GN=LOC100564200 PE=3 SV=1	keratin, type I cytoskeletal 14	68,8	52,2	0	32	14	36	10	458	50,6	5,12
G1KHP6	LOC100567055	IF rod domain-containing protein OS=Anolis carolinensis OX=28377 GN=LOC100567055 PE=3 SV=2	keratin, type II cytoskeletal cochleal	36,8	28,0	0	20	11	23	4	522	56,6	6,04
A0A803TVI4		Keratin, type II cytoskeletal 5 OS=Anolis carolinensis OX=28377 PE=3 SV=1	keratin, type II cytoskeletal 5	9,3	9,9	0	7	3	8	1	450	50,7	7,47
G1KHQ9	LOC100562605	IF rod domain-containing protein OS=Anolis carolinensis OX=28377 GN=LOC100562605 PE=4 SV=3	krt80	6,8	0,0	0	3	3	3	3	1103	120	7,46
A0A803TKX0	LOC100563808	IF rod domain-containing protein OS=Anolis carolinensis OX=28377 GN=LOC100563808 PE=3 SV=1	keratin, type I cytoskeletal 19	5,0	4,4	0	6	3	5	1	436	47,2	5,06
HBS2*		HBS2	HBS2 keratin	3,4	0,0	0	3	2	2	2	754	78,5	7,69
A0A803TVC5		Keratin OS=Anolis carolinensis OX=28377 PE=4 SV=1	CBP, Li-Ac-37	312,9	193,2	0	63	32	68	32	492	43,3	10,4
EDGY2*		EDGY2	EDGY2	133,6	67,9	0	65	15	30	15	324	33,5	8,85
EDSCP*		EDSCP	EDSCP	35,5	18,2	0	9	2	4	2	232	24,5	7,49
A0A803T8V4	LOC100558171	Keratin OS=Anolis carolinensis OX=28377 GN=LOC100558171 PE=3 SV=1	CBP	31,4	18,8	0	16	3	5	3	217	21,6	8,5
H9GCU0	LOC100557315	Keratin OS=Anolis carolinensis OX=28377 GN=LOC100557315 PE=3 SV=2	CBP	22,8	12,2	0	29	4	5	3	184	17,9	5,25
H9GCU2	LOC100558368	Keratin OS=Anolis carolinensis OX=28377 GN=LOC100558368 PE=3 SV=2	CBP	18,9	11,3	0	30	3	5	2	177	17,1	7,61
H9GQG3		Keratin OS=Anolis carolinensis OX=28377 PE=3 SV=2	CBP	18,4	8,2	0	11	1	2	1	170	16,1	8,22
LOR2*		LOR2	LOR2	12,5	4,5	0	14	3	3	3	543	46,8	9,03
A0A803TK53	LOC107983365	Epidermal differentiation protein OS=Anolis carolinensis OX=28377 GN=LOC107983365 PE=4 SV=1	EDWM	10,2	3,0	0	12	3	3	3	329	34,5	8,4
A0A803U1H9	li-ac-35	Keratin OS=Anolis carolinensis OX=28377 GN=li-ac-35 PE=4 SV=1	CBP, Li-Ac-35	4,8	6,4	0	7	1	3	1	136	14,9	9,77
EDCS2*		EDCS2	EDCS2	2,5	0,0	0,003	10	1	1	1	102	10,5	8,02
G1KUH3		Junction plakoglobin OS=Anolis carolinensis OX=28377 PE=3 SV=2		365,3	188,9	0	66	40	73	38	743	81,3	6,1
R4GBL1	PKP1	Plakophilin 1 OS=Anolis carolinensis OX=28377 GN=PKP1 PE=3 SV=1		272,3	151,9	0	64	37	55	37	729	81,3	9,14
H9GPT2	DSG1	Desmoglein 4 OS=Anolis carolinensis OX=28377 GN=DSG1 PE=4 SV=2		249,7	123,6	0	35	29	59	29	1247	130,3	4,73
G1KYJ6		Desmoplakin SH3 domain-containing protein OS=Anolis carolinensis OX=28377 PE=4 SV=3		211,0	98,3	0	29	48	53	48	2001	232	7,15
G1KNU2	PKP3	Plakophilin 3 OS=Anolis carolinensis OX=28377 GN=PKP3 PE=3 SV=3		93,7	46,8	0	33	18	23	18	817	91,4	9,35
A0A803THA1		AHNAK nucleoprotein OS=Anolis carolinensis OX=28377 PE=4 SV=1		1186,7	616,0	0	66	209	286	209	5447	582,5	6,18
H9G6P9		SH3 domain-containing protein OS=Anolis carolinensis OX=28377 PE=4 SV=2		1118,6	638,1	0	65	206	284	206	2879	332,1	6,79
A0A803TC99		SH3 domain-containing protein OS=Anolis carolinensis OX=28377 PE=4 SV=1		1107,2	592,6	0	51	217	279	212	4396	501,3	6,21
A0A803TTS7	COL6A3	Collagen type VI alpha 3 chain OS=Anolis carolinensis OX=28377 GN=COL6A3 PE=4 SV=1		1062,7	608,1	0	52	144	258	144	3094	335,1	6,48
A0A803T1M9	COL12A1	Collagen type XII alpha 1 chain OS=Anolis carolinensis OX=28377 GN=COL12A1 PE=4 SV=1		969,9	571,6	0	50	128	231	128	3118	338,6	5,45
A0A803TYG0		Fibronectin type-III domain-containing protein OS=Anolis carolinensis OX=28377 PE=3 SV=1		700,7	424,0	0	75	97	170	97	1304	147,9	6,09
A0A803TIS5	akap12	A kinase-anchoring proteins AKAP-5 and AKAP-12 calmodulin (CaM)-binding domain-containing protein OS=Anolis carolinensis OX=28377 GN=akap12 PE=4 SV=1		579,2	290,2	0	48	100	128	100	2620	288,4	4,49
H9GH08	LOC100562375	Albumin domain-containing protein OS=Anolis carolinensis OX=28377 GN=LOC100562375 PE=4 SV=2		456,3	262,5	0	67	53	98	1	612	69,2	6,07
A0A803T9W0	LOC100562375	Albumin domain-containing protein OS=Anolis carolinensis OX=28377 GN=LOC100562375 PE=4 SV=1		452,5	262,2	0	65	53	97	1	630	71,2	6,02
G1KDR6	VIM	Vimentin OS=Anolis carolinensis OX=28377 GN=VIM PE=3 SV=3		412,2	234,3	0	69	49	100	45	465	53,2	5,25
G1KSP5	myh9	Myosin-9 OS=Anolis carolinensis OX=28377 GN=myh9 PE=3 SV=1		396,3	196,4	0	37	63	82	52	1960	226,8	5,63
H9G3B2	DYNC1H1	Dynein cytoplasmic 1 heavy chain 1 OS=Anolis carolinensis OX=28377 GN=DYNC1H1 PE=3 SV=2		373,2	165,0	0	26	95	102	95	4682	536,3	6,43
A0A803TH08	dst	SH3 domain-containing protein OS=Anolis carolinensis OX=28377 GN=dst PE=4 SV=1		370,7	171,4	0	34	76	90	75	2621	303,2	6,24
G1KM40	SPTBN1	Spectrin beta chain OS=Anolis carolinensis OX=28377 GN=SPTBN1 PE=3 SV=3		343,0	155,4	0	37	65	82	63	2345	271,8	5,59
G1KD78	COL6A2	Collagen type VI alpha 2 chain OS=Anolis carolinensis OX=28377 GN=COL6A2 PE=4 SV=2		329,3	181,0	0	31	38	63	14	1019	108,4	6,04
H9GPS3	LOC100565442	Cadherin domain-containing protein OS=Anolis carolinensis OX=28377 GN=LOC100565442 PE=4 SV=2		315,2	165,7	0	62	42	69	42	855	94,4	5,71
A0A803TW09	LOC100554738	Transferrin-like domain-containing protein OS=Anolis carolinensis OX=28377 GN=LOC100554738 PE=3 SV=1		287,4	150,9	0	73	48	62	48	707	76,3	6,15
H9GMQ9	ANXA2	Annexin OS=Anolis carolinensis OX=28377 GN=ANXA2 PE=3 SV=2		283,0	155,5	0	65	31	55	31	406	45	8,21
A0A803TY64	actn4	Alpha-actinin-4 OS=Anolis carolinensis OX=28377 GN=actn4 PE=3 SV=1		272,5	149,6	0	56	43	60	1	905	104,1	5,3
A0A803TVW9	actn4	Alpha-actinin-4 OS=Anolis carolinensis OX=28377 GN=actn4 PE=3 SV=1		271,3	150,6	0	54	43	60	1	927	106,6	5,3
A0A803T1B7		EF-hand domain-containing protein OS=Anolis carolinensis OX=28377 PE=4 SV=1		269,7	113,4	0	41	49	56	49	1298	149,4	5,58
A0A803T3H5		Lamin A/C OS=Anolis carolinensis OX=28377 PE=3 SV=1		257,2	133,5	0	66	46	63	45	609	67,4	8,32
H9GBH0	PPL	Periplakin OS=Anolis carolinensis OX=28377 GN=PPL PE=4 SV=2		255,1	139,2	0	34	54	69	54	1762	206	5,47
G1KJT9	flna	Calponin-homology (CH) domain-containing protein OS=Anolis carolinensis OX=28377 GN=flna PE=3 SV=3		245,2	113,0	0	26	47	55	43	2710	287,6	6,2
G1KDW3	COL1A1	Alpha-1 type I collagen OS=Anolis carolinensis OX=28377 GN=COL1A1 PE=4 SV=3		244,9	250,7	0	27	32	157	21	1450	137,5	5,73
G1KKJ8	apob	Apolipoprotein B OS=Anolis carolinensis OX=28377 GN=apob PE=4 SV=2		234,4	105,0	0	17	56	64	56	4522	510,2	6,71
H9G7W7		IQ motif containing GTPase activating protein 1 OS=Anolis carolinensis OX=28377 PE=4 SV=2		226,0	103,4	0	30	46	52	43	1922	217,4	7,42
G1KRK2	HSPA2	Heat shock protein family A (Hsp70) member 2 OS=Anolis carolinensis OX=28377 GN=HSPA2 PE=3 SV=1		223,6	112,9	0	57	38	53	25	635	69,8	5,76
A0A803TES0		Methanethiol oxidase OS=Anolis carolinensis OX=28377 PE=3 SV=1		212,5	134,6	0	75	22	49	13	422	47,3	5,73
A0A803T0Q2	COL6A2	Collagen type VI alpha 2 chain OS=Anolis carolinensis OX=28377 GN=COL6A2 PE=4 SV=1		206,0	117,1	0	23	25	41	1	915	96,8	5,94
G1KT22	ATP5F1B	ATP synthase subunit beta OS=Anolis carolinensis OX=28377 GN=ATP5F1B PE=3 SV=2		205,6	103,0	0	62	21	37	21	528	56,5	5,27
A0A803TLZ0	LOC100567488	Beta-actin OS=Anolis carolinensis OX=28377 GN=LOC100567488 PE=3 SV=1		204,5	124,8	0	65	23	58	1	376	41,8	5,48
R4GCN4	LOC100554364	Heat shock protein family A (Hsp70) member 8 OS=Anolis carolinensis OX=28377 GN=LOC100554364 PE=3 SV=2		204,3	107,4	0	57	31	48	10	652	71,3	5,57
G1KQE0	HSP90AB1	Heat shock protein 90 alpha family class B member 1 OS=Anolis carolinensis OX=28377 GN=HSP90AB1 PE=3 SV=2		203,7	96,1	0	49	33	41	27	727	83,4	5,06
G1KQS3	c3	Complement C3 OS=Anolis carolinensis OX=28377 GN=c3 PE=4 SV=3		194,4	98,6	0	28	41	48	36	1660	186,8	6,4
L7MZZ9	tubb4b	Tubulin beta chain OS=Anolis carolinensis OX=28377 GN=tubb4b PE=3 SV=1		186,8	102,2	0	66	28	35	1	445	49,8	4,89
G1KRT6	FN1	Fibronectin OS=Anolis carolinensis OX=28377 GN=FN1 PE=4 SV=3		181,8	84,3	0	20	30	40	30	2476	273,2	5,71
A0A803TZC8		Moesin OS=Anolis carolinensis OX=28377 GN=MSN PE=4 SV=1		179,9	96,6	0	51	33	45	25	612	71,8	6,58
H9GBL1	GAPDH	Glyceraldehyde-3-phosphate dehydrogenase OS=Anolis carolinensis OX=28377 GN=GAPDH PE=3 SV=1		176,5	89,2	0	57	19	32	19	333	35,7	8,27
H9GMK9	LOC100562036	Tubulin alpha chain OS=Anolis carolinensis OX=28377 GN=LOC100562036 PE=3 SV=3		172,3	106,1	0	68	26	40	1	449	49,9	5,1
L7N034	LOC100555394	Myosin heavy chain 4 OS=Anolis carolinensis OX=28377 GN=LOC100555394 PE=3 SV=2		169,6	87,8	0	22	34	47	9	1865	214,5	5,85
G1KM87	DCN	Decorin OS=Anolis carolinensis OX=28377 GN=DCN PE=3 SV=1		167,6	89,2	0	68	27	49	27	357	39,7	8,31
H9G9N5	HSPA8	Heat shock protein family A (Hsp70) member 8 OS=Anolis carolinensis OX=28377 GN=HSPA8 PE=3 SV=1		167,5	89,9	0	50	28	42	9	646	70,8	5,52
L7MZT7	LOC100553546	Methanethiol oxidase OS=Anolis carolinensis OX=28377 GN=LOC100553546 PE=3 SV=2		163,8	83,9	0	75	32	39	20	454	50,9	5,6
A0A803TE62		AHNAK nucleoprotein OS=Anolis carolinensis OX=28377 PE=4 SV=1		162,1	78,1	0	40	30	34	10	1468	156,5	5,06
A0A803T4D9	myh3	Myosin heavy chain 3 OS=Anolis carolinensis OX=28377 GN=myh3 PE=3 SV=1		156,3	77,2	0	20	27	35	4	1727	198,3	5,94
H9GI83	VCP	Transitional endoplasmic reticulum ATPase OS=Anolis carolinensis OX=28377 GN=VCP PE=3 SV=2		153,9	73,6	0	53	32	38	20	807	89,4	5,3
A0A803TUG7	LOC100561212	Tubulin alpha chain OS=Anolis carolinensis OX=28377 GN=LOC100561212 PE=3 SV=1		153,9	83,9	0	71	25	35	7	433	48,3	5,01
H9GHJ1	TLN1	Talin 1 OS=Anolis carolinensis OX=28377 GN=TLN1 PE=4 SV=3		153,7	68,0	0	21	34	39	32	2292	244,7	6,39
H9GNH2	SFN	Stratifin OS=Anolis carolinensis OX=28377 GN=SFN PE=3 SV=1		152,0	86,0	0	79	21	33	20	246	27,8	5,07
A0A803T951	FBN1	Fibrillin 1 OS=Anolis carolinensis OX=28377 GN=FBN1 PE=4 SV=1		150,6	74,4	0	15	31	40	30	2202	239,9	4,78
A0A803SLL7	myh11	Myosin heavy chain 14 OS=Anolis carolinensis OX=28377 GN=myh11 PE=3 SV=1		147,8	71,5	0	20	31	34	23	1990	230,2	5,72
G1KNZ5	COL1A2	Collagen type I alpha 2 chain OS=Anolis carolinensis OX=28377 GN=COL1A2 PE=4 SV=2		145,9	193,2	0	18	21	132	18	1354	128,6	9,16
G1KKD7	ANXA6	Annexin OS=Anolis carolinensis OX=28377 GN=ANXA6 PE=3 SV=2		145,4	67,5	0	48	27	31	27	693	77,5	5,73
A0A803T2G8	CLTC	Clathrin heavy chain OS=Anolis carolinensis OX=28377 GN=CLTC PE=3 SV=1		144,0	72,2	0	21	28	32	21	1694	193,5	5,74
G1KB81	LMNB1	Lamin B1 OS=Anolis carolinensis OX=28377 GN=LMNB1 PE=3 SV=2		142,2	72,4	0	54	30	36	29	591	66,9	5,15
A0A803T1H6		VWFA domain-containing protein OS=Anolis carolinensis OX=28377 PE=4 SV=1		141,7	70,4	0	58	16	28	16	284	31,6	6,34
H9GTP6		Uncharacterized protein OS=Anolis carolinensis OX=28377 PE=4 SV=2		140,2	47,1	0	13	42	48	39	5755	647,5	5,52
R4GAH2	PKM	Pyruvate kinase OS=Anolis carolinensis OX=28377 GN=PKM PE=3 SV=2		139,4	77,8	0	54	22	28	3	527	57,6	7,31
A0A803TQG2	FLNB	Filamin B OS=Anolis carolinensis OX=28377 GN=FLNB PE=3 SV=1		138,5	63,7	0	15	27	31	23	2634	281,8	5,49
H9GD14	sptbn2	Spectrin beta chain OS=Anolis carolinensis OX=28377 GN=sptbn2 PE=3 SV=2		138,2	64,3	0	18	31	35	29	2278	261,3	5,64
G1KNV6	VAT1	Vesicle amine transport 1 OS=Anolis carolinensis OX=28377 GN=VAT1 PE=3 SV=3		138,0	75,6	0	56	17	24	17	410	43,8	6,13
H9G3B6	hsp90aa1	Heat shock protein HSP 90-alpha OS=Anolis carolinensis OX=28377 GN=hsp90aa1 PE=3 SV=2		137,3	66,1	0	47	29	40	23	728	84,1	5,05
G1KL13		Uncharacterized protein OS=Anolis carolinensis OX=28377 PE=4 SV=3		132,3	71,8	0	42	25	29	5	1434	152,4	5,36
L7MZG9	ACTG2	Actin, gamma-enteric smooth muscle OS=Anolis carolinensis OX=28377 GN=ACTG2 PE=3 SV=1		127,9	78,0	0	56	19	44	2	376	41,9	5,48
H9G8M3	hspa5	78 kDa glucose-regulated protein OS=Anolis carolinensis OX=28377 GN=hspa5 PE=3 SV=2		127,5	65,9	0	42	24	28	22	655	72,3	5,16
A0A803SPD9	VCL	Vinculin OS=Anolis carolinensis OX=28377 GN=VCL PE=3 SV=1		126,7	60,0	0	27	21	26	21	1066	116,8	6,18
H9GHQ2	PKM	Pyruvate kinase OS=Anolis carolinensis OX=28377 GN=PKM PE=3 SV=2		126,0	70,7	0	48	20	26	1	527	57,7	6,84
G1KQX8	LOC100557873	Alpha-2-macroglobulin-like protein 1 OS=Anolis carolinensis OX=28377 GN=LOC100557873 PE=3 SV=3		125,6	65,1	0	24	25	31	23	1442	162,2	5,11
H9G990	EEF1A1	Elongation factor 1-alpha OS=Anolis carolinensis OX=28377 GN=EEF1A1 PE=3 SV=1		125,1	77,4	0	47	17	26	11	462	50,1	9,07
G1KDJ9	LOC100562152	Tubulin alpha chain OS=Anolis carolinensis OX=28377 GN=LOC100562152 PE=3 SV=2		124,6	72,7	0	51	20	30	6	449	49,8	5,14
H9GGM8	LOC100556620	Globin domain-containing protein OS=Anolis carolinensis OX=28377 GN=LOC100556620 PE=3 SV=1		123,4	59,0	0	76	13	25	13	142	16	7,97
G1KE69	VILL	Villin like OS=Anolis carolinensis OX=28377 GN=VILL PE=3 SV=2		122,5	62,5	0	34	22	27	21	862	97	6,02
H9GL50		Methanethiol oxidase OS=Anolis carolinensis OX=28377 PE=3 SV=2		121,8	66,6	0	52	15	25	2	458	51	5,72
A0A803T2N4		Alpha-macroglobulin receptor-binding domain-containing protein OS=Anolis carolinensis OX=28377 PE=4 SV=1		120,7	58,1	0	40	18	28	9	543	60,1	6,43
A0A803TGE9	ACTN1	Alpha-actinin-1 OS=Anolis carolinensis OX=28377 GN=ACTN1 PE=3 SV=1		118,7	57,1	0	33	22	26	11	888	102,8	5,44
A0A803T134		PDZ domain-containing protein OS=Anolis carolinensis OX=28377 PE=4 SV=1		118,0	60,4	0	43	20	22	1	1353	145,4	5,12
H9G4K1		Aldehyde dehydrogenase family 3 member A2 OS=Anolis carolinensis OX=28377 PE=3 SV=3		117,8	64,5	0	33	19	28	19	563	62,6	8,13
G1KP96	LTA4H	Leukotriene A(4) hydrolase OS=Anolis carolinensis OX=28377 GN=LTA4H PE=3 SV=2		112,5	49,5	0	38	21	27	21	613	69,3	6,49
R4GDL8	LOC100567732	PLA2c domain-containing protein OS=Anolis carolinensis OX=28377 GN=LOC100567732 PE=4 SV=1		112,5	52,7	0	45	22	27	22	599	68	8,12
H9GLQ2	ALDOA	Fructose-bisphosphate aldolase OS=Anolis carolinensis OX=28377 GN=ALDOA PE=3 SV=1		112,2	69,1	0	58	18	26	18	364	39,6	8,44
A0A803TU95	hnrnpa3	RRM domain-containing protein OS=Anolis carolinensis OX=28377 GN=hnrnpa3 PE=4 SV=1		111,7	45,7	0	48	14	20	14	362	37,5	8,4
A0A803TJX1	MYH10	Myosin heavy chain 10 OS=Anolis carolinensis OX=28377 GN=MYH10 PE=3 SV=1		111,2	47,4	0	14	25	28	17	2097	242,8	6,01
A0A803TY90	DPYSL3	Dihydropyrimidinase like 3 OS=Anolis carolinensis OX=28377 GN=DPYSL3 PE=3 SV=1		108,5	52,6	0	50	22	28	21	682	73,3	6,09
G1KLU8	ANXA1	Annexin OS=Anolis carolinensis OX=28377 GN=ANXA1 PE=3 SV=1		108,4	48,0	0	67	21	27	21	342	38,3	7,14
L7MZS1		Methanethiol oxidase OS=Anolis carolinensis OX=28377 PE=3 SV=2		108,3	58,5	0	49	16	28	2	474	52,9	5,49
H9GJS5	cct3	T-complex protein 1 subunit gamma OS=Anolis carolinensis OX=28377 GN=cct3 PE=3 SV=2		106,8	47,7	0	46	19	23	19	545	60,3	7,23
A0A803TJX9		Tubulin beta chain OS=Anolis carolinensis OX=28377 PE=3 SV=1		106,4	62,2	0	50	18	22	5	357	40	4,89
H9G7M9		phosphoribosylformylglycinamidine synthase OS=Anolis carolinensis OX=28377 PE=3 SV=3		106,0	56,1	0	25	19	25	19	1280	138,5	5,57
G1KMZ9	ANXA5	Annexin OS=Anolis carolinensis OX=28377 GN=ANXA5 PE=3 SV=2		105,8	65,1	0	58	16	24	16	321	36,3	5,83
A0A803SX86	ATP5F1A	ATP synthase subunit alpha OS=Anolis carolinensis OX=28377 GN=ATP5F1A PE=3 SV=1		105,7	52,2	0	50	22	28	22	560	60,5	9,13
H9G4M1		RRM domain-containing protein OS=Anolis carolinensis OX=28377 PE=4 SV=3		105,2	55,2	0	35	22	26	22	640	69,3	4,93
G1KJG1	IDH1	Isocitrate dehydrogenase [NADP] OS=Anolis carolinensis OX=28377 GN=IDH1 PE=3 SV=1		103,9	52,6	0	45	21	24	20	415	46,8	7,71
G1KKB8	P4HB	Protein disulfide-isomerase OS=Anolis carolinensis OX=28377 GN=P4HB PE=3 SV=3		103,5	53,2	0	56	19	26	19	511	57,3	4,75
G1KRC2	ATIC	Bifunctional purine biosynthesis protein ATIC OS=Anolis carolinensis OX=28377 GN=ATIC PE=3 SV=2		102,6	51,2	0	45	16	19	16	593	64,4	8,27
G1KI82	LOC100552501	glutathione transferase OS=Anolis carolinensis OX=28377 GN=LOC100552501 PE=3 SV=2		102,2	57,3	0	62	14	23	6	239	27,8	6,27
G1K9R8	HNRNPK	Heterogeneous nuclear ribonucleoprotein K OS=Anolis carolinensis OX=28377 GN=HNRNPK PE=4 SV=2		101,1	57,7	0	47	17	26	17	426	47,1	5,88
H9GK64	LOC100554762	RRM domain-containing protein OS=Anolis carolinensis OX=28377 GN=LOC100554762 PE=4 SV=1		100,6	48,1	0	59	9	14	8	152	16,5	8,92
H9GA84		Creatine kinase M-type OS=Anolis carolinensis OX=28377 PE=3 SV=2		99,1	53,3	0	50	13	20	12	312	35,1	6,95
H9GLK9	mvp	Major vault protein OS=Anolis carolinensis OX=28377 GN=mvp PE=4 SV=2		97,4	52,8	0	34	20	24	20	858	95,4	5,39
G1KD77		VWFA domain-containing protein OS=Anolis carolinensis OX=28377 PE=4 SV=2		97,3	78,7	0	26	13	37	13	427	47	6
H9G9P8	PDIA3	Protein disulfide-isomerase OS=Anolis carolinensis OX=28377 GN=PDIA3 PE=3 SV=2		97,2	43,5	0	34	17	23	17	587	65,1	8,78
G1KNK2	tmpo	Thymopoietin OS=Anolis carolinensis OX=28377 GN=TMPO PE=3 SV=2		96,9	52,1	0	37	15	20	15	456	50,7	8,98
H9GLK7	glud1	Glutamate dehydrogenase 1, mitochondrial OS=Anolis carolinensis OX=28377 GN=glud1 PE=3 SV=1		96,0	51,5	0	32	17	23	6	549	60,5	8,05
A0A803TS86	TRIM29	Tripartite motif containing 29 OS=Anolis carolinensis OX=28377 GN=TRIM29 PE=4 SV=1		95,6	46,1	0	33	18	22	18	543	62,4	7,5
A0A803T503	ESYT1	Extended synaptotagmin 1 OS=Anolis carolinensis OX=28377 GN=ESYT1 PE=4 SV=1		95,6	41,1	0	28	23	27	23	1042	116,8	6,06
G1KE14	UBA1	E1 ubiquitin-activating enzyme OS=Anolis carolinensis OX=28377 GN=UBA1 PE=3 SV=1		93,8	36,4	0	28	21	24	21	1059	118,9	5,64
G1KIK2	OGN	Mimecan OS=Anolis carolinensis OX=28377 GN=OGN PE=3 SV=2		93,8	51,0	0	38	17	23	17	302	34,4	5,6
A0A803SYH4		EH domain containing 2 OS=Anolis carolinensis OX=28377 PE=4 SV=1		93,2	46,8	0	49	16	21	15	421	48,2	6,49
G1KWY8	LOC100560217	Serpin domain-containing protein OS=Anolis carolinensis OX=28377 GN=LOC100560217 PE=3 SV=2		93,2	48,4	0	39	11	15	11	428	48,3	6,46
A0A803T9A6		Inter-alpha-trypsin inhibitor heavy chain H3 OS=Anolis carolinensis OX=28377 PE=3 SV=1		91,0	49,3	0	22	14	17	10	864	97,2	5,71
H9GFC4	HSPD1	60 kDa heat shock protein, mitochondrial OS=Anolis carolinensis OX=28377 GN=HSPD1 PE=3 SV=2		90,3	39,6	0	30	19	20	19	677	71,9	8,31
A0A803TLZ1	rpsa	Small ribosomal subunit protein uS2 OS=Anolis carolinensis OX=28377 GN=rpsa PE=3 SV=1		89,9	42,1	0	64	14	17	14	296	32,9	4,87
G1KGF3	NPEPPS	Aminopeptidase puromycin sensitive OS=Anolis carolinensis OX=28377 GN=NPEPPS PE=3 SV=3		89,7	37,5	0	28	21	24	21	1059	118,2	6,27
H9GF00	ALDH2	Aldehyde dehydrogenase, mitochondrial OS=Anolis carolinensis OX=28377 GN=ALDH2 PE=3 SV=2		89,3	43,0	0	35	17	20	16	555	60,9	7,85
G1KNT0	aldh9a1	4-trimethylaminobutyraldehyde dehydrogenase OS=Anolis carolinensis OX=28377 GN=aldh9a1 PE=3 SV=2		88,6	46,4	0	45	16	19	16	496	54	5,97
A0A803TA20	LOC100566382	Catalase OS=Anolis carolinensis OX=28377 GN=LOC100566382 PE=3 SV=1		88,5	52,1	0	37	17	19	14	592	67,2	8,76
G1KBK2		Rab GDP dissociation inhibitor OS=Anolis carolinensis OX=28377 PE=3 SV=3		87,9	35,7	0	45	16	18	15	424	48,1	6,7
H9G589	MDH2	Malate dehydrogenase OS=Anolis carolinensis OX=28377 GN=MDH2 PE=3 SV=3		87,8	43,4	0	57	14	17	14	339	35,8	8,48

Suppl. Table S9. Proteomic analysis of claws of *P. vitticeps*

Accession (Uniprot) or new annotation*	Gene Symbol	Description	Note	Sum PEP Score	Score Sequest HT: Sequest HT	Exp. q-value: Combined	Coverage [%]	# Peptides	# PSMs	# Unique Peptides	# AAs	MW [kDa]	calc. pI
A0A6J0T4Z5	LOC110074843	Keratin, type I cuticular Ha6-like OS=Pogona vitticeps OX=103695 GN=LOC110074843 PE=3 SV=1	**KRT36L1 - hair keratin homolog type I**	359,045	296,5	<0,001	63	44	178	44	397	45	4,81
A0A6J0T2W2	LOC110074898	Keratin, type I cytoskeletal 14-like OS=Pogona vitticeps OX=103695 GN=LOC110074898 PE=3 SV=1	KRT14L1	141,948	70,4	<0,001	59	28	43	26	449	49,5	5,55
A0A6J0T2V3	LOC110074892	Keratin, type I cytoskeletal 14-like OS=Pogona vitticeps OX=103695 GN=LOC110074892 PE=3 SV=1	KRT14L2	135,845	62,69	<0,001	57	28	39	25	458	50,5	5,12
A0A6J0T2L7	LOC110074851	Keratin, type I cuticular Ha6-like OS=Pogona vitticeps OX=103695 GN=LOC110074851 PE=4 SV=1	**KRT36L2 - hair keratin homolog type I**	58,788	35,99	<0,001	27	16	21	15	447	50,4	5,55
A0A6J0SXS2	LOC110074850	Keratin, type I cytoskeletal 15-like isoform X1 OS=Pogona vitticeps OX=103695 GN=LOC110074850 PE=3 SV=1	KRT15	2,075	0	<0,001	6	2	2	1	513	54,1	5,34
A0A6J0SDW8	LOC110070014	Keratin, type II cuticular Hb5-like OS=Pogona vitticeps OX=103695 GN=LOC110070014 PE=4 SV=1	**KRT84L1 - hair keratin homolog type II**	658,769	556,73	<0,001	91	85	338	82	525	58,7	6,02
A0A6J0SA49	LOC110070015	Keratin, type II cytoskeletal cochleal-like isoform X1 OS=Pogona vitticeps OX=103695 GN=LOC110070015 PE=3 SV=1	**KRT84L4 - hair keratin homolog type II**	210,06	94,18	<0,001	66	47	71	44	531	57,3	7,66
A0A6J0SEM6	LOC110070016	Keratin, type II cytoskeletal 8-like OS=Pogona vitticeps OX=103695 GN=LOC110070016 PE=3 SV=1	**KRT84L2 - hair keratin homolog type II**	111,503	49,79	<0,001	59	36	44	33	554	62,3	6,09
A0A6J0SA54	LOC110070018	Keratin, type II cytoskeletal cochleal-like OS=Pogona vitticeps OX=103695 GN=LOC110070018 PE=3 SV=1	KRT5L4	110,718	56,04	<0,001	46	25	36	22	417	46,9	5,48
KRT84L3	LOC110070017	Hb2 keratin prediction	**KRT84L3 - hair keratin homolog type II**	34,56	11,34	<0,001	18	12	13	12	549	61,6	8,25
A0A6J0VBD4	LOC110090296	Keratin, type II cytoskeletal 5-like isoform X2 OS=Pogona vitticeps OX=103695 GN=LOC110090296 PE=3 SV=1	KRT5L2	31,355	19,51	<0,001	18	10	11	8	608	63,3	7,12
A0A6J0VFV2	LOC110090297	Keratin, type II cytoskeletal cochleal-like OS=Pogona vitticeps OX=103695 GN=LOC110090297 PE=4 SV=1	KRT78L2	5,386	2,55	<0,001	6	2	2	2	654	66,8	8,29
CBP1*	CBP1	CBP1		43,405	18,28	<0,001	43	12	15	12	357	37,3	7,31
EDCCL2*	EDCCL2	EDCCL2	**EDCCL2**	31,643	24,38	<0,001	37	3	13	3	126	12,6	7,36
EDGY1*	EDGY1	EDGY1		7,062	2,63	<0,001	21	3	3	3	129	14,7	8,76
EDYM1*	EDYM1	EDYM1	**EDYM1**	6,176	2,2	<0,001	24	3	5	3	194	20,6	8,76
EDCCL4*	EDCCL4	EDCCL4	**EDCCL4**	6,049	3,17	<0,001	37	1	1	1	71	7,7	5,92
EDCCL1*	EDCCL1	EDCCL1	**EDCCL1**	2,648	0	<0,001	12	1	1	1	122	12,4	4,72
EDCP*	EDCP	EDCP		1,42	1,74	0,004	3	1	1	1	229	23	7,66
A0A6J0TYQ4	DSP	Desmoplakin OS=Pogona vitticeps OX=103695 GN=DSP PE=4 SV=1		84,672	24,77	<0,001	9	27	34	27	2879	332,1	6,77
A0A6J0SNS3	PKP1	Plakophilin-1 OS=Pogona vitticeps OX=103695 GN=PKP1 PE=3 SV=1		42,757	12,57	<0,001	29	14	16	14	739	82,2	9,13
A0A6J0TDZ5	LOC110077646	Desmoglein-1-beta-like OS=Pogona vitticeps OX=103695 GN=LOC110077646 PE=4 SV=1		20,434	10,2	<0,001	5	3	4	3	1224	128,3	4,78
A0A6J0T2Q4	JUP	Junction plakoglobin OS=Pogona vitticeps OX=103695 GN=JUP PE=3 SV=1		8,454	4,13	<0,001	8	4	4	4	748	82,2	6,19
A0A6J0UWW8	LOC110087251	Desmocollin-1-like isoform X1 OS=Pogona vitticeps OX=103695 GN=LOC110087251 PE=4 SV=1		6,615	5,61	<0,001	4	2	2	2	904	100,3	5,34
A0A6J0STP6	PKP3	Plakophilin-3 OS=Pogona vitticeps OX=103695 GN=PKP3 PE=3 SV=1		5,514	0	<0,001	4	3	3	3	835	92,8	9,41
A0A6J0UB89	COL6A3	Collagen alpha-3(VI) chain isoform X2 OS=Pogona vitticeps OX=103695 GN=COL6A3 PE=3 SV=1		211,867	105,44	<0,001	19	49	60	49	3156	342,4	6,47
A0A6J0SYP5	COL12A1	Collagen alpha-1(XII) chain OS=Pogona vitticeps OX=103695 GN=COL12A1 PE=4 SV=1		155,55	57,87	<0,001	19	47	59	47	3106	337,6	5,48
A0A6J0V7Y0	LOC110089918	Alpha-fetoprotein-like OS=Pogona vitticeps OX=103695 GN=LOC110089918 PE=4 SV=1		99,882	31,45	<0,001	39	22	30	15	613	69,7	6,15
A0A6J0VDX6	LOC110091302	Tubulin beta chain OS=Pogona vitticeps OX=103695 GN=LOC110091302 PE=3 SV=1		95,159	49	<0,001	56	18	23	2	444	49,6	4,92
A0A6J0V2P6	TUBB4B	Tubulin beta chain OS=Pogona vitticeps OX=103695 GN=TUBB4B PE=3 SV=1		84,486	43,25	<0,001	52	17	22	1	445	49,8	4,89
A0A6J0UH78	LOC110084467	Actin, cytoplasmic type 5-like OS=Pogona vitticeps OX=103695 GN=LOC110084467 PE=3 SV=1		79,401	45,06	<0,001	42	15	33	15	376	41,8	5,58
A0A6J0UNM8	VIM	Vimentin OS=Pogona vitticeps OX=103695 GN=VIM PE=3 SV=1		76,102	31,42	<0,001	44	21	23	20	462	53	5,17
A0A6J0VJJ0	LMNA	Lamin OS=Pogona vitticeps OX=103695 GN=LMNA PE=3 SV=1		72,047	29,26	<0,001	29	19	22	19	665	74,5	6,86
A0A6J0T377	ANXA2	Annexin OS=Pogona vitticeps OX=103695 GN=ANXA2 PE=3 SV=1		67,798	29,26	<0,001	39	12	16	12	339	38,6	8,05
A0A6J0VE48	AHNAK	LOW QUALITY PROTEIN: neuroblast differentiation-associated protein AHNAK OS=Pogona vitticeps OX=103695 GN=AHNAK PE=4 SV=1		61,329	10,85	<0,001	12	22	25	22	5378	574,3	5,96
A0A6J0U9C9	GSN	Gelsolin OS=Pogona vitticeps OX=103695 GN=GSN PE=3 SV=1		60,599	25,9	<0,001	35	15	19	1	603	66,5	6,73
A0A6J0UB74	HSPA2	Heat shock-related 70 kDa protein 2 OS=Pogona vitticeps OX=103695 GN=HSPA2 PE=3 SV=1		59,118	20,58	<0,001	32	16	23	8	636	70,1	5,95
A0A6J0UEN6	GSN	Gelsolin OS=Pogona vitticeps OX=103695 GN=GSN PE=3 SV=1		58,496	25,04	<0,001	28	15	19	1	753	83,3	6,1
A0A6J0T3Z2	LOC110075272	Ovotransferrin-like OS=Pogona vitticeps OX=103695 GN=LOC110075272 PE=3 SV=1		57,234	29,55	<0,001	29	15	17	15	702	76,1	6,87
A0A6J0U1G5	COL7A1	Collagen alpha-1(VII) chain OS=Pogona vitticeps OX=103695 GN=COL7A1 PE=4 SV=1		54,758	38,65	<0,001	6	15	20	15	2914	300,6	5,81
A0A6J0VBQ9	HSPB6	Heat shock protein beta-6 OS=Pogona vitticeps OX=103695 GN=HSPB6 PE=3 SV=1		50,291	13,8	<0,001	65	9	14	9	171	19,3	6,96
A0A6J0TGC1	LOC110078124	Hemoglobin subunit beta-1-like isoform X1 OS=Pogona vitticeps OX=103695 GN=LOC110078124 PE=3 SV=1		48,997	28,33	<0,001	71	9	11	4	147	16,3	8,47
A0A6J0UWX5	LOC110087640	Histone H2B OS=Pogona vitticeps OX=103695 GN=LOC110087640 PE=3 SV=1		45,992	15,65	<0,001	59	13	16	1	126	14	10,27
A0A6J0V5V6	LOC110089112	Hemoglobin subunit alpha-D-like OS=Pogona vitticeps OX=103695 GN=LOC110089112 PE=3 SV=1		45,774	17,33	<0,001	46	5	13	5	142	16	7,18
A0A6J0VBV3	LOC110089939	Alpha-fetoprotein-like isoform X1 OS=Pogona vitticeps OX=103695 GN=LOC110089939 PE=4 SV=1		44,437	14,25	<0,001	20	13	18	6	612	69,6	6,42
A0A6J0V8B5	POSTN	Periostin isoform X1 OS=Pogona vitticeps OX=103695 GN=POSTN PE=4 SV=1		44,196	7	<0,001	19	17	23	17	896	99,9	7,3
A0A6J0SFD4	LOC110071744	Histone H2B OS=Pogona vitticeps OX=103695 GN=LOC110071744 PE=3 SV=1		43,46	15,65	<0,001	59	13	15	1	126	14	10,32
A0A6J0TWC0	TRIM29	Tripartite motif-containing protein 29 OS=Pogona vitticeps OX=103695 GN=TRIM29 PE=4 SV=1		42,785	10,97	<0,001	19	9	10	9	599	67,8	7,46
A0A6J0VGY4	COL6A1	Collagen alpha-1(VI) chain OS=Pogona vitticeps OX=103695 GN=COL6A1 PE=4 SV=1		42,565	10,25	<0,001	11	12	14	12	1017	108,5	5,74
A0A6J0VCT5	LOC110089618	Heat shock cognate 71 kDa protein-like isoform X1 OS=Pogona vitticeps OX=103695 GN=LOC110089618 PE=3 SV=1		41,998	12,74	<0,001	27	14	19	4	644	70,6	5,57
A0A6J0T108	LOC110075416	Leukocyte elastase inhibitor-like OS=Pogona vitticeps OX=103695 GN=LOC110075416 PE=3 SV=1		41,81	20,27	<0,001	14	5	8	5	450	50,9	5,4
A0A6J0VF06	COL6A2	Collagen alpha-2(VI) chain isoform X1 OS=Pogona vitticeps OX=103695 GN=COL6A2 PE=4 SV=1		40,943	27,07	<0,001	11	8	13	8	1018	108,1	5,76
A0A6J0SSP7	ENO1	phosphopyruvate hydratase OS=Pogona vitticeps OX=103695 GN=ENO1 PE=3 SV=1		40,836	19,47	<0,001	21	5	8	3	434	47,2	7,08
A0A6J0SMQ8	LOC110071759	Histone H3-like OS=Pogona vitticeps OX=103695 GN=LOC110071759 PE=3 SV=1		40,346	17,29	<0,001	39	13	18	13	242	27	11,39
A0A6J0UNX2	GAPDH	Glyceraldehyde-3-phosphate dehydrogenase OS=Pogona vitticeps OX=103695 GN=GAPDH PE=3 SV=1		35,453	16,03	<0,001	28	5	5	5	333	35,8	8,54
A0A6J0TJ00	LOC110078124	Hemoglobin subunit beta-1-like isoform X2 OS=Pogona vitticeps OX=103695 GN=LOC110078124 PE=3 SV=1		34,193	17,56	<0,001	43	6	9	1	147	16,4	8,7
A0A6J0TM70	COL1A1	Collagen alpha-1(I) chain OS=Pogona vitticeps OX=103695 GN=COL1A1 PE=4 SV=1		33,383	19,04	<0,001	6	7	9	7	1453	137,9	5,87
A0A6J0SYH1	LOC110073662	Tubulin alpha chain OS=Pogona vitticeps OX=103695 GN=LOC110073662 PE=3 SV=1		32,957	18,64	<0,001	25	6	8	4	451	50,1	5,06
A0A6J0TKE3	HSPA8	Heat shock cognate 71 kDa protein OS=Pogona vitticeps OX=103695 GN=HSPA8 PE=3 SV=1		32,135	6,7	<0,001	25	14	16	5	646	70,8	5,52
A0A6J0T0H5	COL1A2	Collagen alpha-2(I) chain OS=Pogona vitticeps OX=103695 GN=COL1A2 PE=4 SV=1		31,63	17,81	<0,001	10	10	11	10	1363	129,2	8,94
A0A6J0T3F0	EEF1A1	Elongation factor 1-alpha OS=Pogona vitticeps OX=103695 GN=EEF1A1 PE=3 SV=1		31,478	17,81	<0,001	17	5	6	5	462	50,1	9,07
A0A6J0T442	LGALS1	Galectin OS=Pogona vitticeps OX=103695 GN=LGALS1 PE=4 SV=1		29,519	13,8	<0,001	53	7	11	7	134	14,6	6,67
A0A6J0URI5	SFN	14-3-3 protein sigma OS=Pogona vitticeps OX=103695 GN=SFN PE=3 SV=1		28,815	10,53	<0,001	38	8	9	8	246	27,9	5,01
A0A6J0S9P1	DCN	Decorin OS=Pogona vitticeps OX=103695 GN=DCN PE=3 SV=1		27,747	11,47	<0,001	30	7	9	7	357	39,5	8,69
A0A6J0SNF4	HNRNPA2B1	Heterogeneous nuclear ribonucleoproteins A2/B1 isoform X7 OS=Pogona vitticeps OX=103695 GN=HNRNPA2B1 PE=4 SV=1		26,79	10,55	<0,001	35	5	7	5	320	34,5	9,13
A0A6J0V7R7	TNC	LOW QUALITY PROTEIN: tenascin OS=Pogona vitticeps OX=103695 GN=TNC PE=4 SV=1		26,55	3,59	<0,001	8	9	13	9	1434	158,3	5,49
A0A6J0V3Y8	LOC110089136	Hemoglobin subunit alpha-1-like OS=Pogona vitticeps OX=103695 GN=LOC110089136 PE=3 SV=1		25,723	9,99	<0,001	23	2	2	2	97	10,7	8,09
A0A6J0T2U3	ACTN4	Alpha-actinin-4 OS=Pogona vitticeps OX=103695 GN=ACTN4 PE=3 SV=1		25,595	7,93	<0,001	16	9	10	5	904	104,3	5,25
A0A6J0TNT3	TUBB6	Tubulin beta chain OS=Pogona vitticeps OX=103695 GN=TUBB6 PE=3 SV=1		25,146	14,94	<0,001	16	6	8	1	446	50	4,84
A0A6J0SUG5	PGK1	Phosphoglycerate kinase OS=Pogona vitticeps OX=103695 GN=PGK1 PE=3 SV=1		23,947	6,44	<0,001	15	5	5	5	417	44,5	7,71
A0A6J0TRD6	BGN	Biglycan OS=Pogona vitticeps OX=103695 GN=BGN PE=3 SV=1		22,899	10,05	<0,001	7	1	2	1	368	41,5	6,68
A0A6J0TQ90	LOC110079741	Uricase OS=Pogona vitticeps OX=103695 GN=LOC110079741 PE=3 SV=1		22,764	9,96	<0,001	17	5	6	5	328	37,4	6,84
A0A6J0UT19	RPL4	60S ribosomal protein L4 OS=Pogona vitticeps OX=103695 GN=RPL4 PE=3 SV=1		22,318	6,66	<0,001	23	5	7	5	372	42,1	10,95
A0A6J0V0N4	TPM3	Tropomyosin alpha-3 chain isoform X8 OS=Pogona vitticeps OX=103695 GN=TPM3 PE=3 SV=1		21,234	10,63	<0,001	17	3	5	2	254	29,2	4,75
A0A6J0U4Z9	ANXA1	Annexin OS=Pogona vitticeps OX=103695 GN=ANXA1 PE=3 SV=1		19,747	2,91	<0,001	24	5	6	5	264	29,6	6,58
A0A6J0U1S9	LOC110081855	Histone H1-like OS=Pogona vitticeps OX=103695 GN=LOC110081855 PE=3 SV=1		19,467	10,87	<0,001	27	5	6	2	225	22,7	10,92
A0A6J0UWY0	LOC110086246	Protein-glutamine gamma-glutamyltransferase E-like OS=Pogona vitticeps OX=103695 GN=LOC110086246 PE=3 SV=1	TGM	19,43	6,38	<0,001	10	4	5	4	680	75	7,27
A0A6J0T843	LOC110076065	Histone H1.10 OS=Pogona vitticeps OX=103695 GN=LOC110076065 PE=3 SV=1		19,368	9,85	<0,001	29	6	6	4	215	21,7	10,98
A0A6J0SEP3	LOC110071417	Histone H2A OS=Pogona vitticeps OX=103695 GN=LOC110071417 PE=3 SV=1		19,243	4,6	<0,001	56	7	9	6	129	13,9	10,9
A0A6J0VAZ2	LOC110090193	A.superbus venom factor 1-like OS=Pogona vitticeps OX=103695 GN=LOC110090193 PE=4 SV=1		18,855	3,19	<0,001	7	9	10	9	1654	185,8	6,43
A0A6J0SZC0	TPM1	Tropomyosin alpha-1 chain OS=Pogona vitticeps OX=103695 GN=TPM1 PE=3 SV=1		18,531	9,08	<0,001	13	3	4	2	336	38,5	4,97
A0A6J0UEN1	HSPA5	78 kDa glucose-regulated protein OS=Pogona vitticeps OX=103695 GN=HSPA5 PE=3 SV=1		18,323	1,84	<0,001	11	7	9	5	651	72	5,14
A0A6J0SAM2	CFL1	Cofilin-1 OS=Pogona vitticeps OX=103695 GN=CFL1 PE=3 SV=1		17,635	8,89	<0,001	31	4	5	3	167	18,7	8,02
A0A6J0TE35	PKM	Pyruvate kinase OS=Pogona vitticeps OX=103695 GN=PKM PE=3 SV=1		17,501	7,76	<0,001	21	5	6	5	527	57,5	7,71
A0A6J0T1S0	MSN	Moesin OS=Pogona vitticeps OX=103695 GN=MSN PE=4 SV=1		17,011	4,51	<0,001	8	6	6	6	577	68	6,37
A0A6J0T7K1	MYH9	Myosin-9 OS=Pogona vitticeps OX=103695 GN=MYH9 PE=3 SV=1		16,629	4,24	<0,001	6	8	8	7	1960	226,6	5,6
A0A6J0T411	LOC110075966	Histone H1.0 OS=Pogona vitticeps OX=103695 GN=LOC110075966 PE=3 SV=1		15,251	4,63	<0,001	29	5	6	5	192	20,4	10,84
A0A6J0SJC9	EEF2	Elongation factor 2 OS=Pogona vitticeps OX=103695 GN=EEF2 PE=4 SV=1		14,977	4,46	<0,001	8	5	5	2	858	95,3	6,93
A0A6J0V7A6	LOC110088484	Protein S100-A7-like OS=Pogona vitticeps OX=103695 GN=LOC110088484 PE=4 SV=1		14,786	9,24	<0,001	29	3	4	3	121	13,6	5,82
A0A6J0UK52	LOC110085344	Histone H2A OS=Pogona vitticeps OX=103695 GN=LOC110085344 PE=3 SV=1		14,538	5,04	<0,001	34	4	5	3	128	13,6	10,58
A0A6J0UW73	RPS14	Small ribosomal subunit protein uS11 OS=Pogona vitticeps OX=103695 GN=RPS14 PE=3 SV=1		14,378	5,42	<0,001	21	3	4	3	151	16,3	10,05
A0A6J0SXT0	RPL8	Large ribosomal subunit protein uL2 OS=Pogona vitticeps OX=103695 GN=RPL8 PE=3 SV=1		13,931	3,68	<0,001	16	3	4	3	257	28	10,96
A0A6J0T6C4	ACTN2	Alpha-actinin-2 OS=Pogona vitticeps OX=103695 GN=ACTN2 PE=3 SV=1		13,907	9,8	<0,001	6	4	6	1	895	103,7	5,59
A0A6J0TD66	ACTN1	Alpha-actinin-1 OS=Pogona vitticeps OX=103695 GN=ACTN1 PE=3 SV=1		13,605	5,4	<0,001	7	5	6	1	914	105,5	5,35
A0A6J0SQA9	ANXA5	Annexin OS=Pogona vitticeps OX=103695 GN=ANXA5 PE=3 SV=1		13,175	7,99	<0,001	10	3	4	3	319	35,9	6,35
A0A6J0UB03	VCP	Transitional endoplasmic reticulum ATPase OS=Pogona vitticeps OX=103695 GN=VCP PE=3 SV=1		12,857	6,49	<0,001	8	5	5	5	846	94,2	5,48
A0A6J0U0U1	LOC110081254	glutathione transferase OS=Pogona vitticeps OX=103695 GN=LOC110081254 PE=3 SV=1		12,697	6,09	<0,001	7	1	2	1	221	25	9,01
A0A6J0TR51	FLNA	Filamin-A OS=Pogona vitticeps OX=103695 GN=FLNA PE=3 SV=1		12,112	2,46	<0,001	4	7	7	7	2652	281,4	6,1
A0A6J0UHB6	RPS13	Small ribosomal subunit protein uS15 OS=Pogona vitticeps OX=103695 GN=RPS13 PE=3 SV=1		12,019	6,2	<0,001	26	2	2	2	151	17,2	10,62
A0A6J0UG87	ALDOC	Fructose-bisphosphate aldolase OS=Pogona vitticeps OX=103695 GN=ALDOC PE=3 SV=1		11,512	11,19	<0,001	14	3	4	2	364	39,5	5,47
A0A6J0U140	ALDOA	Fructose-bisphosphate aldolase OS=Pogona vitticeps OX=103695 GN=ALDOA PE=3 SV=1		11,507	9,41	<0,001	8	2	3	1	364	39,6	8,24
A0A6J0UNM0	OGN	Mimecan OS=Pogona vitticeps OX=103695 GN=OGN PE=3 SV=1		11,215	2,63	<0,001	10	4	5	4	307	34,9	5,73
A0A6J0U503	RPL19	Ribosomal protein L19 OS=Pogona vitticeps OX=103695 GN=RPL19 PE=3 SV=1		10,622	0	<0,001	29	5	5	5	196	23,5	11,47
A0A6J0TX71	HNRNPUL2	Heterogeneous nuclear ribonucleoprotein U-like protein 2 OS=Pogona vitticeps OX=103695 GN=HNRNPUL2 PE=4 SV=1		10,613	3,71	<0,001	7	5	5	5	773	87,3	4,87
A0A6J0V3M5	LOC110088264	Histone H1-like OS=Pogona vitticeps OX=103695 GN=LOC110088264 PE=3 SV=1		10,488	5,38	<0,001	16	2	3	1	240	23,9	10,99
A0A6J0TEI4	UBB	Polyubiquitin-B OS=Pogona vitticeps OX=103695 GN=UBB PE=4 SV=1		10,464	4,6	<0,001	38	5	5	5	229	25,8	7,49
A0A6J0U018	LOC110081556	DnaJ homolog subfamily A member 1-like OS=Pogona vitticeps OX=103695 GN=LOC110081556 PE=3 SV=1		10,287	5,39	<0,001	9	3	4	3	409	46	8
A0A6J0UNK9	ASPN	Asporin OS=Pogona vitticeps OX=103695 GN=ASPN PE=3 SV=1		10,258	4,63	<0,001	12	2	2	2	372	42,3	7,94
A0A6J0TT98	VDAC2	Voltage-dependent anion-selective channel protein 2 OS=Pogona vitticeps OX=103695 GN=VDAC2 PE=3 SV=1		10,165	2,83	<0,001	15	3	3	3	320	34,7	9,11
A0A6J0UD27	RPS15A	Small ribosomal subunit protein uS8 OS=Pogona vitticeps OX=103695 GN=RPS15A PE=3 SV=1		10,15	1,68	<0,001	28	4	4	4	130	14,8	9,55
A0A6J0TVA7	SERBP1	Plasminogen activator inhibitor 1 RNA-binding protein isoform X1 OS=Pogona vitticeps OX=103695 GN=SERBP1 PE=3 SV=1		10,127	4,1	<0,001	11	2	3	2	460	48,8	7,18
A0A6J0V754	LOC110088463	Protein S100 OS=Pogona vitticeps OX=103695 GN=LOC110088463 PE=3 SV=1		10,071	4,8	<0,001	34	3	3	3	101	11,4	6,54
A0A6J0VCC3	P4HB	Protein disulfide-isomerase OS=Pogona vitticeps OX=103695 GN=P4HB PE=3 SV=1		10,056	4,62	<0,001	8	3	3	3	518	58,3	4,78
A0A6J0U4D1	EIF5A	Eukaryotic translation initiation factor 5A OS=Pogona vitticeps OX=103695 GN=EIF5A PE=3 SV=1		10,025	3,44	<0,001	25	2	2	2	154	16,8	5,24
A0A6J0UPQ6	PLEC	Plectin isoform X9 OS=Pogona vitticeps OX=103695 GN=PLEC PE=4 SV=1		9,998	0	<0,001	1	6	6	6	4586	523,8	6,1
A0A6J0V0Z8	LOC110088549	L-lactate dehydrogenase OS=Pogona vitticeps OX=103695 GN=LOC110088549 PE=3 SV=1		9,976	2,53	<0,001	15	4	4	3	332	36,6	7,88
A0A6J0V4A1	PGAM1	Phosphoglycerate mutase OS=Pogona vitticeps OX=103695 GN=PGAM1 PE=3 SV=1		9,881	3,22	<0,001	14	2	2	2	263	30,2	6,65
A0A6J0STL3	CSRP1	Cysteine and glycine-rich protein 1 OS=Pogona vitticeps OX=103695 GN=CSRP1 PE=4 SV=1		9,605	3,12	<0,001	28	3	3	3	193	20,4	8,57
A0A6J0SKS3	RPS3A	Small ribosomal subunit protein eS1 OS=Pogona vitticeps OX=103695 GN=RPS3A PE=3 SV=1		9,423	6,37	<0,001	11	2	3	2	264	29,8	9,72
A0A6J0T1L3	DUSP14	Dual specificity protein phosphatase 14 OS=Pogona vitticeps OX=103695 GN=DUSP14 PE=3 SV=1		9,347	2,54	<0,001	23	4	4	4	198	22,7	9,69
A0A6J0U461	MPZ	Myelin protein P0 OS=Pogona vitticeps OX=103695 GN=MPZ PE=3 SV=1		9,178	6,15	<0,001	15	2	3	2	248	27,7	9,48
A0A6J0SVD0	P4HA1	Prolyl 4-hydroxylase subunit alpha-1 OS=Pogona vitticeps OX=103695 GN=P4HA1 PE=3 SV=1		9,161	2,05	<0,001	9	3	3	3	625	71,6	7,55
A0A6J0UMZ2	RPS17	Small ribosomal subunit protein eS17 OS=Pogona vitticeps OX=103695 GN=RPS17 PE=3 SV=1		9,118	3,02	<0,001	5	2	2	2	378	44,2	10,08
A0A6J0TDZ9	PDIA3	Protein disulfide-isomerase OS=Pogona vitticeps OX=103695 GN=PDIA3 PE=3 SV=1		8,799	0	<0,001	7	4	5	4	502	56,3	5,95
A0A6J0SE73	LUM	Lumican OS=Pogona vitticeps OX=103695 GN=LUM PE=3 SV=1		8,585	4,08	<0,001	7	3	4	3	349	39,6	6,37
A0A6J0UNB6	LOC110085931	Elongation factor 2-like OS=Pogona vitticeps OX=103695 GN=LOC110085931 PE=4 SV=1		8,485	0	<0,001	6	4	4	1	859	95,6	6,87
A0A6J0SKW6	FGG	Fibrinogen gamma chain OS=Pogona vitticeps OX=103695 GN=FGG PE=4 SV=1		8,4	4,38	<0,001	7	2	3	2	434	49,5	5,59

Suppl. Table S9. Proteomic analysis of claws of *P. vitticeps* (continued)

Accession (Uniprot) or new annotation*	Gene Symbol	Description	Note	Sum PEP Score	Score Sequest HT: Sequest HT	Exp. q-value: Combined	Coverage [%]	# Peptides	# PSMs	# Unique Peptides	# AAs	MW [kDa]	calc. pI
A0A6J0U133	LOC110080992	Glucosylceramidase OS=Pogona vitticeps OX=103695 GN=LOC110080992 PE=3 SV=1		8,389	3,27	<0,001	17	3	3	3	333	38,6	6
A0A6J0TUR4	LOC110080606	Protein-glutamine gamma-glutamyltransferase 5-like OS=Pogona vitticeps OX=103695 GN=LOC110080606 PE=3 SV=1	TGM9	8,305	4,31	<0,001	4	3	5	3	778	87,4	6,71
A0A6J0VBM6	RPSA	Small ribosomal subunit protein uS2 OS=Pogona vitticeps OX=103695 GN=RPSA PE=3 SV=1		8,296	5,03	<0,001	18	4	4	4	296	32,9	4,87
A0A6J0UNX7	PTMS	Parathymosin isoform X1 OS=Pogona vitticeps OX=103695 GN=PTMS PE=3 SV=1		8,024	3,55	<0,001	17	1	1	1	105	12	4,35
A0A6J0TEN2	RPS6	40S ribosomal protein S6 OS=Pogona vitticeps OX=103695 GN=RPS6 PE=3 SV=1		7,889	0	<0,001	25	5	6	5	249	28,7	10,84
A0A6J0UXS9	ITIH2	Inter-alpha-trypsin inhibitor heavy chain H2 OS=Pogona vitticeps OX=103695 GN=ITIH2 PE=4 SV=1		7,53	2,64	<0,001	2	2	3	2	796	88,7	7,62
A0A6J0TT96	LOC110080185	40S ribosomal protein S4 OS=Pogona vitticeps OX=103695 GN=LOC110080185 PE=3 SV=1		7,511	2,74	<0,001	11	2	2	2	263	29,6	10,15
A0A6J0UKQ9	TPI1	Triosephosphate isomerase OS=Pogona vitticeps OX=103695 GN=TPI1 PE=3 SV=1		7,467	0	<0,001	21	4	4	4	248	26,7	7,15
A0A6J0UPE4	HNRNPA3	Heterogeneous nuclear ribonucleoprotein A3 OS=Pogona vitticeps OX=103695 GN=HNRNPA3 PE=4 SV=1		7,373	2,81	<0,001	15	3	3	2	376	39,4	8,78
A0A6J0T540	EIF3A	Eukaryotic translation initiation factor 3 subunit A OS=Pogona vitticeps OX=103695 GN=EIF3A PE=3 SV=1		7,368	2,28	<0,001	6	4	4	4	1336	159,9	6,93
A0A6J0V970	SERPINH1	Serpin H1 OS=Pogona vitticeps OX=103695 GN=SERPINH1 PE=3 SV=1		7,359	2,47	<0,001	10	4	4	4	471	53,1	9,39
A0A6J0SV67	ATP5B	ATP synthase subunit beta OS=Pogona vitticeps OX=103695 GN=ATP5B PE=3 SV=1		7,246	0	<0,001	11	3	3	3	531	56,6	5,41
A0A6J0VJX3	LAMC2	Laminin subunit gamma-2 OS=Pogona vitticeps OX=103695 GN=LAMC2 PE=4 SV=1		7,241	2,55	<0,001	3	2	2	2	1170	128,4	6,84
A0A6J0SU07	RPS26	40S ribosomal protein S26 OS=Pogona vitticeps OX=103695 GN=RPS26 PE=3 SV=1		7,216	2,42	<0,001	22	2	2	2	115	13	11
A0A6J0UIR7	BLMH	Bleomycin hydrolase OS=Pogona vitticeps OX=103695 GN=BLMH PE=3 SV=1		7,212	0	<0,001	10	4	4	4	459	53,3	6,24
A0A6J0V606	GDI2	Rab GDP dissociation inhibitor OS=Pogona vitticeps OX=103695 GN=GDI2 PE=3 SV=1		7,209	3,88	<0,001	8	2	2	2	434	49,1	6,18
A0A6J0TA36	LOC110076508	Uncharacterized protein LOC110076508 OS=Pogona vitticeps OX=103695 GN=LOC110076508 PE=3 SV=1		7,122	2,2	<0,001	3	3	3	3	1146	123,1	8,62
A0A6J0VH69	LOC110090680	Alpha-1-antiproteinase 2-like OS=Pogona vitticeps OX=103695 GN=LOC110090680 PE=3 SV=1		7,105	3,89	<0,001	6	2	3	2	404	45,6	6,2
A0A6J0TZM2	LOC110081523	Methanethiol oxidase OS=Pogona vitticeps OX=103695 GN=LOC110081523 PE=3 SV=1		6,956	2,29	<0,001	9	3	3	3	471	52,5	6,15
A0A6J0SRT8	HSPG2	LOW QUALITY PROTEIN: basement membrane-specific heparan sulfate proteoglycan core protein OS=Pogona vitticeps OX=103695 GN=HSPG2 PE=4 SV=1		6,802	0	<0,001	1	3	4	3	3995	429	5,97
A0A6J0U3W9	S100A11	Protein S100 OS=Pogona vitticeps OX=103695 GN=S100A11 PE=3 SV=1		6,723	2,47	<0,001	21	3	3	3	109	12,1	7,28
A0A6J0SS38	LOC110072471	Cytochrome c OS=Pogona vitticeps OX=103695 GN=LOC110072471 PE=3 SV=1		6,59	2	<0,001	26	2	3	2	105	11,8	9,41
A0A6J0V3W9	AHSG	Alpha-2-HS-glycoprotein OS=Pogona vitticeps OX=103695 GN=AHSG PE=4 SV=1		6,588	2,48	<0,001	11	2	2	2	351	38,2	6,68
A0A6J0T800	ST13	Hsc70-interacting protein OS=Pogona vitticeps OX=103695 GN=ST13 PE=3 SV=1		6,404	2,5	<0,001	9	2	2	2	362	40,4	5,06
A0A6J0UYI5	ACAT2	acetyl-CoA C-acetyltransferase OS=Pogona vitticeps OX=103695 GN=ACAT2 PE=3 SV=1		6,376	2,09	<0,001	6	3	3	3	459	48,1	8,22
A0A6J0SX62	RPS7	40S ribosomal protein S7 OS=Pogona vitticeps OX=103695 GN=RPS7 PE=3 SV=1		6,356	2,47	<0,001	18	2	2	2	194	22,1	10,1
A0A6J0T7D3	CTSD	Cathepsin D OS=Pogona vitticeps OX=103695 GN=CTSD PE=3 SV=1		6,291	2,88	<0,001	12	3	3	3	399	43,8	5,91
A0A6J0TDK9	RPL21	60S ribosomal protein L21 OS=Pogona vitticeps OX=103695 GN=RPL21 PE=3 SV=1		6,251	1,75	<0,001	16	2	3	2	160	18,7	10,48
A0A6J0UND1	ATP5A1	ATP synthase subunit alpha OS=Pogona vitticeps OX=103695 GN=ATP5A1 PE=3 SV=1		6,229	2,03	<0,001	7	4	4	4	553	59,9	9,13
A0A6J0V2M5	RPL3	Large ribosomal subunit protein uL3 OS=Pogona vitticeps OX=103695 GN=RPL3 PE=3 SV=1		6,229	1,78	<0,001	11	4	4	4	403	46,1	10,26
A0A6J0T078	HSP90AB1	Heat shock protein HSP 90-beta OS=Pogona vitticeps OX=103695 GN=HSP90AB1 PE=3 SV=1		6,142	0	<0,001	2	3	3	3	727	83,4	5,07
A0A6J0TTK4	SOD1	Superoxide dismutase [Cu-Zn] OS=Pogona vitticeps OX=103695 GN=SOD1 PE=3 SV=1		6,021	4,76	<0,001	23	2	2	2	164	16,8	6,67
A0A6J0TUG1	RPL10A	Ribosomal protein OS=Pogona vitticeps OX=103695 GN=RPL10A PE=3 SV=1		6,009	0	<0,001	18	3	3	3	217	24,6	9,82
A0A6J0UGD9	PPIA	Peptidyl-prolyl cis-trans isomerase OS=Pogona vitticeps OX=103695 GN=PPIA PE=3 SV=1		5,999	3,96	<0,001	16	1	1	1	165	17,9	7,42
A0A6J0VBV7	LOC110089944	Alpha-fetoprotein-like OS=Pogona vitticeps OX=103695 GN=LOC110089944 PE=4 SV=1		5,883	3,1	<0,001	3	1	1	1	608	69,4	5,08
A0A6J0TP95	HSPA9	Stress-70 protein, mitochondrial OS=Pogona vitticeps OX=103695 GN=HSPA9 PE=3 SV=1		5,847	0	<0,001	6	2	2	2	670	72,8	6,2
A0A6J0SDR5	LOC110071136	Alpha-2-macroglobulin-like OS=Pogona vitticeps OX=103695 GN=LOC110071136 PE=4 SV=1		5,82	1,69	<0,001	7	2	2	2	402	43,3	8,27
A0A6J0U455	ATIC	Bifunctional purine biosynthesis protein ATIC OS=Pogona vitticeps OX=103695 GN=ATIC PE=3 SV=1		5,805	0	<0,001	7	2	2	2	595	64,5	8
A0A6J0V9J0	LOC110089440	Uncharacterized protein LOC110089440 OS=Pogona vitticeps OX=103695 GN=LOC110089440 PE=4 SV=1		5,762	3,92	<0,001	4	2	2	2	201	22,4	4,65
A0A6J0U242	LOC110081979	Cytochrome c oxidase subunit 6A1, mitochondrial OS=Pogona vitticeps OX=103695 GN=LOC110081979 PE=3 SV=1		5,722	7,98	<0,001	11	1	3	1	115	12,8	9,88
A0A6J0V0M4	LOC110088485	Protein S100-A9-like OS=Pogona vitticeps OX=103695 GN=LOC110088485 PE=4 SV=1		5,673	6,35	<0,001	53	3	3	3	118	13,4	6,68
A0A6J0U6T7	TLN1	LOW QUALITY PROTEIN: talin-1 OS=Pogona vitticeps OX=103695 GN=TLN1 PE=4 SV=1		5,571	2,1	<0,001	1	2	2	2	2436	258,4	6,13
A0A6J0TXJ0	LOC110081131	RNA-binding protein 3-like isoform X1 OS=Pogona vitticeps OX=103695 GN=LOC110081131 PE=4 SV=1		5,564	0	<0,001	13	2	2	2	152	16,6	8,92
A0A6J0T7Z2	ACO2	Aconitate hydratase, mitochondrial OS=Pogona vitticeps OX=103695 GN=ACO2 PE=3 SV=1		5,543	4,08	<0,001	4	1	1	1	748	82	7,09
A0A6J0UA06	AGT	Angiotensinogen OS=Pogona vitticeps OX=103695 GN=AGT PE=3 SV=1		5,526	5,6	<0,001	3	1	2	1	454	51,3	5,77
A0A6J0U986	LOC110081962	glutathione transferase OS=Pogona vitticeps OX=103695 GN=LOC110081962 PE=3 SV=1		5,446	0	<0,001	9	1	2	1	256	29,2	8,1
A0A6J0SVW8	RPS8	40S ribosomal protein S8 OS=Pogona vitticeps OX=103695 GN=RPS8 PE=3 SV=1		5,428	1,93	<0,001	12	2	2	2	208	24,2	10,23
A0A6J0VBW9	WDR1	WD repeat-containing protein 1 isoform X3 OS=Pogona vitticeps OX=103695 GN=WDR1 PE=3 SV=1		5,414	2,16	<0,001	7	2	2	2	512	56,7	6,83
A0A6J0TWT1	PLS3	Plastin-3 OS=Pogona vitticeps OX=103695 GN=PLS3 PE=4 SV=1		5,376	1,66	<0,001	8	3	3	3	628	70,6	5,73
A0A6J0UNJ9	DPYSL3	Dihydropyrimidinase-related protein 3 isoform X1 OS=Pogona vitticeps OX=103695 GN=DPYSL3 PE=3 SV=1		5,307	2,45	<0,001	3	2	2	2	671	72,5	6,32
A0A6J0SAG0	LOC110070085	Ribonuclease-like OS=Pogona vitticeps OX=103695 GN=LOC110070085 PE=3 SV=1		5,28	0	<0,001	20	3	3	3	141	15,8	10,1
A0A6J0V1D6	RBMX	RNA-binding motif protein, X chromosome isoform X2 OS=Pogona vitticeps OX=103695 GN=RBMX PE=4 SV=1		5,228	2,97	<0,001	4	1	2	1	380	41,1	10,17
A0A6J0U5P2	LOC110081327	Uncharacterized protein LOC110081327 OS=Pogona vitticeps OX=103695 GN=LOC110081327 PE=3 SV=1		5,228	2,35	<0,001	16	2	2	2	257	28,1	8,06
A0A6J0SZY7	RPL9	Large ribosomal subunit protein uL6 OS=Pogona vitticeps OX=103695 GN=RPL9 PE=3 SV=1		5,171	0	<0,001	14	2	2	2	192	21,8	10,14
A0A6J0SUQ6	HNRNPA1	Heterogeneous nuclear ribonucleoprotein A1 isoform X1 OS=Pogona vitticeps OX=103695 GN=HNRNPA1 PE=4 SV=1		5,145	0	<0,001	17	4	4	3	365	38	8,21
A0A6J0U404	EIF4A1	Eukaryotic initiation factor 4A-I OS=Pogona vitticeps OX=103695 GN=EIF4A1 PE=3 SV=1		5,124	0	<0,001	12	2	2	2	406	46,1	5,48
A0A6J0V8T1	CFL2	Cofilin-2 OS=Pogona vitticeps OX=103695 GN=CFL2 PE=3 SV=1		5,101	0	<0,001	13	2	2	1	166	18,7	7,88
A0A6J0T8Y4	RPL13	60S ribosomal protein L13 OS=Pogona vitticeps OX=103695 GN=RPL13 PE=3 SV=1		5,076	0	<0,001	13	2	2	2	211	24,4	11,65
A0A6J0TYZ5	LOC110081325	Uncharacterized protein LOC110081325 isoform X3 OS=Pogona vitticeps OX=103695 GN=LOC110081325 PE=3 SV=1		5,041	2,26	<0,001	10	1	1	1	211	22,6	7,88
A0A6J0V203	FKBP9	peptidylprolyl isomerase OS=Pogona vitticeps OX=103695 GN=FKBP9 PE=4 SV=1		5,031	3,26	<0,001	3	1	2	1	584	64,8	5,3
A0A6J0VDV9	RPS2	Small ribosomal subunit protein uS5 OS=Pogona vitticeps OX=103695 GN=RPS2 PE=3 SV=1		5,03	0	<0,001	9	3	3	3	372	40,5	10,29
A0A6J0UBU0	RPL35	Large ribosomal subunit protein uL29 OS=Pogona vitticeps OX=103695 GN=RPL35 PE=4 SV=1		4,995	0	<0,001	24	2	2	2	99	11,7	11,3
A0A6J0UM22	YWHAE	14-3-3 protein epsilon OS=Pogona vitticeps OX=103695 GN=YWHAE PE=3 SV=1		4,971	2,73	<0,001	12	1	1	1	233	26,5	4,87
A0A6J0UDC7	CCT3	T-complex protein 1 subunit gamma OS=Pogona vitticeps OX=103695 GN=CCT3 PE=3 SV=1		4,752	0	<0,001	5	3	3	3	545	60,4	7,15
A0A6J0UQZ9	ETFB	Electron transfer flavoprotein subunit beta OS=Pogona vitticeps OX=103695 GN=ETFB PE=3 SV=1		4,668	0	<0,001	13	2	2	2	257	27,9	8,1
A0A6J0U626	LOC110081888	Myosin-10-like isoform X3 OS=Pogona vitticeps OX=103695 GN=LOC110081888 PE=3 SV=1		4,597	0	<0,001	2	2	2	1	2021	233,4	5,67
A0A6J0TDU3	IQGAP1	Ras GTPase-activating-like protein IQGAP1 OS=Pogona vitticeps OX=103695 GN=IQGAP1 PE=4 SV=1		4,561	1,83	<0,001	2	2	2	2	1659	189,1	6,68
A0A6J0T835	LOC110075898	Fatty acid-binding protein 5 OS=Pogona vitticeps OX=103695 GN=LOC110075898 PE=3 SV=1		4,535	2,34	<0,001	10	1	1	1	135	15,2	7,06
A0A6J0TD68	MATN4	Matrilin-4 OS=Pogona vitticeps OX=103695 GN=MATN4 PE=4 SV=1		4,417	0	<0,001	8	3	4	3	622	69,5	7,02
A0A6J0SFT2	LOC110070601	Tubulin alpha chain OS=Pogona vitticeps OX=103695 GN=LOC110070601 PE=3 SV=1		4,376	2,04	<0,001	8	3	3	2	452	50,5	5,36
A0A6J0UWG3	TTLL12	Tubulin--tyrosine ligase-like protein 12 OS=Pogona vitticeps OX=103695 GN=TTLL12 PE=4 SV=1		4,359	0	<0,001	3	2	2	2	625	73	5,31
A0A6J0UYL4	ENDOD1	Endonuclease domain-containing 1 protein OS=Pogona vitticeps OX=103695 GN=ENDOD1 PE=4 SV=1		4,3	0	<0,001	2	2	2	2	488	53,4	5,73
A0A6J0V223	CKB	creatine kinase OS=Pogona vitticeps OX=103695 GN=CKB PE=3 SV=1		4,29	0	<0,001	10	3	3	3	381	42,8	6,14
A0A6J0UAB1	CDH13	Cadherin-13 OS=Pogona vitticeps OX=103695 GN=CDH13 PE=4 SV=1		4,266	2,07	<0,001	3	1	1	1	712	78,2	5,01
A0A6J0UC50	CAP1	Adenylyl cyclase-associated protein OS=Pogona vitticeps OX=103695 GN=CAP1 PE=3 SV=1		4,261	2,73	<0,001	5	1	1	1	475	51,6	8,16
A0A6J0T3J2	RPL24	Large ribosomal subunit protein eL24 OS=Pogona vitticeps OX=103695 GN=RPL24 PE=3 SV=1		4,25	0	<0,001	20	3	3	3	157	17,7	11,25
A0A6J0UX03	HSPB8	Heat shock protein beta-8 OS=Pogona vitticeps OX=103695 GN=HSPB8 PE=3 SV=1		4,243	0	<0,001	14	2	2	2	154	16,9	8
A0A6J0STY0	CD44	CD44 antigen OS=Pogona vitticeps OX=103695 GN=CD44 PE=4 SV=1		4,24	2,41	<0,001	1	1	1	1	1049	115,4	4,82
A0A6J0V4G9	ILF2	Interleukin enhancer-binding factor 2 OS=Pogona vitticeps OX=103695 GN=ILF2 PE=4 SV=1		4,18	2,43	<0,001	5	1	1	1	352	38,9	4,94
A0A6J0U1W0	ECM1	Extracellular matrix protein 1 isoform X2 OS=Pogona vitticeps OX=103695 GN=ECM1 PE=4 SV=1		4,163	0	<0,001	5	2	2	2	357	39,6	5,1
A0A6J0UBH2	TAGLN2	Transgelin OS=Pogona vitticeps OX=103695 GN=TAGLN2 PE=3 SV=1		4,149	1,95	<0,001	6	2	2	2	199	22,2	8,37
A0A6J0T122	SERPINB5	Serpin B5 OS=Pogona vitticeps OX=103695 GN=SERPINB5 PE=3 SV=1		4,148	0	<0,001	6	3	3	3	375	42,8	5,91
A0A6J0VEJ7	IDH2	Isocitrate dehydrogenase [NADP] OS=Pogona vitticeps OX=103695 GN=IDH2 PE=3 SV=1		4,048	2,18	<0,001	3	1	1	1	400	44,9	7,52
A0A6J0TY39	LOC110081153	Transforming protein RhoA-like OS=Pogona vitticeps OX=103695 GN=LOC110081153 PE=4 SV=1		4,022	0	<0,001	14	2	2	2	193	21,8	6,1
A0A6J0VEP0	METAP2	Methionine aminopeptidase 2 OS=Pogona vitticeps OX=103695 GN=METAP2 PE=3 SV=1		3,982	0	<0,001	3	1	2	1	408	45,5	5,9
A0A6J0U3T9	RPL18	60S ribosomal protein L18 OS=Pogona vitticeps OX=103695 GN=RPL18 PE=3 SV=1		3,981	0	<0,001	11	2	2	2	188	21,6	11,78
A0A6J0SPZ5	SUOX	sulfite oxidase OS=Pogona vitticeps OX=103695 GN=SUOX PE=4 SV=1		3,93	0	<0,001	4	2	2	2	540	59,6	6,29
A0A6J0T6V2	PLD3	5'-3' exonuclease PLD3 OS=Pogona vitticeps OX=103695 GN=PLD3 PE=3 SV=1		3,895	2,08	<0,001	3	1	1	1	540	60,2	6,21
A0A6J0UE10	PRDX5	Peroxiredoxin-5 OS=Pogona vitticeps OX=103695 GN=PRDX5 PE=3 SV=1		3,894	0	<0,001	9	1	1	1	206	21,6	9,19
A0A6J0UGB6	RPS16	40S ribosomal protein S16 OS=Pogona vitticeps OX=103695 GN=RPS16 PE=3 SV=1		3,822	0	<0,001	22	2	2	2	146	16,4	10,21
A0A6J0UUU5	ACAA1	3-ketoacyl-CoA thiolase, peroxisomal OS=Pogona vitticeps OX=103695 GN=ACAA1 PE=3 SV=1		3,807	2,32	<0,001	4	1	1	1	428	44,5	8,73
A0A6J0VCB2	ACAA2	3-ketoacyl-CoA thiolase, mitochondrial OS=Pogona vitticeps OX=103695 GN=ACAA2 PE=3 SV=1		3,797	2,21	<0,001	3	1	1	1	397	42	7,87
A0A6J0TLI1	APOA1	Apolipoprotein A-I OS=Pogona vitticeps OX=103695 GN=APOA1 PE=3 SV=1		3,766	0	<0,001	10	2	2	2	279	32,4	6,33
A0A6J0UXV7	LOC110087420	Aldehyde dehydrogenase OS=Pogona vitticeps OX=103695 GN=LOC110087420 PE=3 SV=1		3,749	2,51	<0,001	6	1	1	1	462	51,6	7,43
A0A6J0U414	LOC110080991	Glucosylceramidase-like OS=Pogona vitticeps OX=103695 GN=LOC110080991 PE=4 SV=1		3,745	1,65	<0,001	17	2	2	2	109	11,9	8,28
A0A6J0U9K9	LOC110082539	Microtubule-associated protein OS=Pogona vitticeps OX=103695 GN=LOC110082539 PE=4 SV=1		3,738	0	<0,001	1	1	1	1	2614	273,9	7,14
A0A6J0TLU1	RPL13A	Large ribosomal subunit protein uL13 OS=Pogona vitticeps OX=103695 GN=RPL13A PE=3 SV=1		3,724	0	<0,001	4	1	1	1	271	30,8	10,99
A0A6J0TWI2	EIF4A3	RNA helicase OS=Pogona vitticeps OX=103695 GN=EIF4A3 PE=3 SV=1		3,722	2,09	<0,001	3	1	1	1	410	46,6	6,73
A0A6J0SQ15	FGB	Fibrinogen beta chain OS=Pogona vitticeps OX=103695 GN=FGB PE=4 SV=1		3,697	0	<0,001	11	3	3	3	497	56,1	8,41
A0A6J0T1C6	CS	Citrate synthase OS=Pogona vitticeps OX=103695 GN=CS PE=3 SV=1		3,672	2,05	<0,001	3	1	1	1	469	52	8,25
A0A6J0UN91	AHCY	Adenosylhomocysteinase OS=Pogona vitticeps OX=103695 GN=AHCY PE=3 SV=1		3,668	0	<0,001	12	3	3	3	433	47,5	6,71
A0A6J0STJ4	TGFBI	Transforming growth factor-beta-induced protein ig-h3 OS=Pogona vitticeps OX=103695 GN=TGFBI PE=4 SV=1		3,643	5,53	<0,001	3	1	3	1	676	73,9	7,39
A0A6J0V082	ZNF185	Zinc finger protein 185 OS=Pogona vitticeps OX=103695 GN=ZNF185 PE=4 SV=1		3,613	0	<0,001	10	1	2	1	130	15	7,77
A0A6J0SHX6	LOC110071316	Alpha-2-macroglobulin-like OS=Pogona vitticeps OX=103695 GN=LOC110071316 PE=4 SV=1		3,596	0	<0,001	14	2	2	2	266	29,5	9,31
A0A6J0TAC9	F13A1	Coagulation factor XIII A chain OS=Pogona vitticeps OX=103695 GN=F13A1 PE=3 SV=1		3,574	2,86	<0,001	1	1	1	1	738	83,4	6,09
A0A6J0SWW4	DDX3X	RNA helicase OS=Pogona vitticeps OX=103695 GN=DDX3X PE=3 SV=1		3,535	5	<0,001	2	1	2	1	711	78	7,28
A0A6J0SZH3	KCTD12	BTB/POZ domain-containing protein KCTD12 OS=Pogona vitticeps OX=103695 GN=KCTD12 PE=4 SV=1		3,499	0	<0,001	3	1	1	1	381	42,1	9,76
A0A6J0UEQ7	VTN	Vitronectin isoform X1 OS=Pogona vitticeps OX=103695 GN=VTN PE=4 SV=1		3,481	0	<0,001	7	2	2	2	451	51,5	5,5
A0A6J0U6X8	PARK7	protein deglycase OS=Pogona vitticeps OX=103695 GN=PARK7 PE=3 SV=1		3,446	2,44	<0,001	8	1	2	1	189	19,9	6,92
A0A6J0U9A6	RAB14	Ras-related protein Rab-14 OS=Pogona vitticeps OX=103695 GN=RAB14 PE=3 SV=1		3,312	0	<0,001	7	1	1	1	215	23,9	6,21
A0A6J0UNP4	PPP1CA	Serine/threonine-protein phosphatase OS=Pogona vitticeps OX=103695 GN=PPP1CA PE=3 SV=1		3,292	0	<0,001	6	1	2	1	313	35,6	6,76
A0A6J0UVX0	ALAD	Delta-aminolevulinic acid dehydratase OS=Pogona vitticeps OX=103695 GN=ALAD PE=3 SV=1		3,212	1,96	<0,001	3	1	1	1	332	36,2	7,24
A0A6J0U9H5	RPL11	Large ribosomal subunit protein uL5 OS=Pogona vitticeps OX=103695 GN=RPL11 PE=3 SV=1		3,178	1,73	<0,001	12	2	2	2	190	21,5	9,89
A0A6J0TFK1	LMNB1	Lamin-B1 OS=Pogona vitticeps OX=103695 GN=LMNB1 PE=3 SV=1		3,144	0	<0,001	2	1	1	1	591	66,7	5,17
A0A6J0UA83	RPL6	60S ribosomal protein L6 OS=Pogona vitticeps OX=103695 GN=RPL6 PE=3 SV=1		3,108	0	<0,001	4	1	1	1	260	29,8	10,89
A0A6J0TJ91	RPL32	60S ribosomal protein L32 OS=Pogona vitticeps OX=103695 GN=RPL32 PE=3 SV=1		3,053	0	<0,001	10	1	1	1	135	15,9	11,47
A0A6J0TK75	COL18A1	Collagen alpha-1(XVIII) chain OS=Pogona vitticeps OX=103695 GN=COL18A1 PE=4 SV=1		3,05	0	<0,001	1	1	1	1	1842	191,7	5,63
A0A6J0U134	RPL34	Large ribosomal subunit protein eL34 OS=Pogona vitticeps OX=103695 GN=RPL34 PE=3 SV=1		3,043	0	<0,001	21	2	2	2	117	13,3	11,47
A0A6J0UEJ8	RPL12	Large ribosomal subunit protein uL11 OS=Pogona vitticeps OX=103695 GN=RPL12 PE=3 SV=1		3,031	1,67	<0,001	5	1	2	1	165	17,7	9,26
A0A6J0VF15	RPS19	Small ribosomal subunit protein eS19 OS=Pogona vitticeps OX=103695 GN=RPS19 PE=3 SV=1		3,008	2,02	<0,001	7	1	1	1	145	16	10,32
A0A6J0V1R2	QDPR	Dihydropteridine reductase OS=Pogona vitticeps OX=103695 GN=QDPR PE=3 SV=1		2,994	0	<0,001	8	1	1	1	241	25,5	7,36
A0A6J0SRE6	AKR1A1	alcohol dehydrogenase (NADP(+)) OS=Pogona vitticeps OX=103695 GN=AKR1A1 PE=3 SV=1		2,991	2,1	<0,001	9	2	2	2	327	37,2	7,58
A0A6J0VCH2	CLIC1	Chloride intracellular channel protein OS=Pogona vitticeps OX=103695 GN=CLIC1 PE=3 SV=1		2,987	6,58	<0,001	5	1	3	1	241	27,1	5,41
A0A6J0T6Z0	NID1	Nidogen-1 OS=Pogona vitticeps OX=103695 GN=NID1 PE=4 SV=1		2,978	0	<0,001	2	2	2	2	1238	135,8	5,1
A0A6J0TQT0	LOC110078540	Trans-1,2-dihydrobenzene-1,2-diol dehydrogenase OS=Pogona vitticeps OX=103695 GN=LOC110078540 PE=3 SV=1		2,958	0	<0,001	7	2	2	2	352	39,2	6,58
A0A6J0V608	AARS	Alanine--tRNA ligase OS=Pogona vitticeps OX=103695 GN=AARS PE=3 SV=1		2,926	0	<0,001	2	1	1	1	968	107,4	5,57
A0A6J0UJ01	FSCN1	Fascin OS=Pogona vitticeps OX=103695 GN=FSCN1 PE=3 SV=1		2,923	1,6	<0,001	3	2	2	2	356	39,5	7,65
A0A6J0SYZ6	AVIL	Advillin isoform X2 OS=Pogona vitticeps OX=103695 GN=AVIL PE=3 SV=1		2,89	0	<0,001	5	2	2	2	678	77,1	5,22
A0A6J0SP08	RPL36	60S ribosomal protein L36 OS=Pogona vitticeps OX=103695 GN=RPL36 PE=3 SV=1		2,888	0	<0,001	12	2	2	2	105	12,2	11,47
A0A6J0V1U9	NCL	Nucleolin OS=Pogona vitticeps OX=103695 GN=NCL PE=4 SV=1		2,886	0	<0,001	3	2	2	2	694	75,3	4,79
A0A6J0SIC4	LOC110071204	Alpha-2-macroglobulin-like OS=Pogona vitticeps OX=103695 GN=LOC110071204 PE=4 SV=1		2,856	0	<0,001	9	2	2	2	347	38,2	5,81
A0A6J0TGV9	ALDOB	Fructose-bisphosphate aldolase OS=Pogona vitticeps OX=103695 GN=ALDOB PE=3 SV=1		2,853	0	<0,001	8	2	2	2	309	33,3	7,77
A0A6J0UC09	LDHB	L-lactate dehydrogenase OS=Pogona vitticeps OX=103695 GN=LDHB PE=3 SV=1		2,821	1,78	<0,001	6	2	2	1	366	40,2	7,44
A0A6J0V5V8	RPL35A	Large ribosomal subunit protein eL33 OS=Pogona vitticeps OX=103695 GN=RPL35A PE=3 SV=1		2,809	0	<0,001	18	2	2	2	110	12,5	11,24
A0A6J0UQN1	NDRG2	Protein NDRG2 OS=Pogona vitticeps OX=103695 GN=NDRG2 PE=3 SV=1		2,786	0	<0,001	7	1	1	1	395	43,4	6,34
A0A6J0SRY5	ELAVL1	ELAV-like protein OS=Pogona vitticeps OX=103695 GN=ELAVL1 PE=3 SV=1		2,782	0	<0,001	14	2	2	2	326	36,2	9,04

Suppl. Table S9. Proteomic analysis of claws of *P. vitticeps* (continued)

Accession (Uniprot) or new annotation*	Gene Symbol	Description	Note	Sum PEP Score	Score Sequest HT: Sequest HT	Exp. q-value: Combined	Coverage [%]	# Peptides	# PSMs	# Unique Peptides	# AAs	MW [kDa]	calc. pI
A0A6J0UH63	LOC110084734	Large ribosomal subunit protein uL15 OS=Pogona vitticeps OX=103695 GN=LOC110084734 PE=3 SV=1		2,763	0	<0,001	20	2	2	2	148	16,8	10,68
A0A6J0USW5	RPS21	40S ribosomal protein S21 OS=Pogona vitticeps OX=103695 GN=RPS21 PE=3 SV=1		2,703	1,94	<0,001	12	1	1	1	83	9,1	8,5
A0A6J0VAN8	FBL	rRNA 2'-O-methyltransferase fibrillarin OS=Pogona vitticeps OX=103695 GN=FBL PE=3 SV=1		2,693	0	<0,001	6	1	1	1	328	34,8	10,54
A0A6J0V8E3	LOC110090046	Large ribosomal subunit protein uL22 OS=Pogona vitticeps OX=103695 GN=LOC110090046 PE=3 SV=1		2,657	0	<0,001	11	2	2	2	184	21,4	10,18
A0A6J0TM02	LOC110078579	17-beta-hydroxysteroid dehydrogenase 14-like isoform X1 OS=Pogona vitticeps OX=103695 GN=LOC110078579 PE=4 SV=1		2,653	1,79	<0,001	3	1	1	1	292	31	5,53
A0A6J0UGL7	MLF2	Myeloid leukemia factor 2 OS=Pogona vitticeps OX=103695 GN=MLF2 PE=3 SV=1		2,64	0	<0,001	7	1	1	1	243	27,6	7,03
A0A6J0V005	LOC110088341	T-kininogen 1-like OS=Pogona vitticeps OX=103695 GN=LOC110088341 PE=4 SV=1		2,601	0	<0,001	3	1	1	1	556	62	6,04
A0A6J0SJE2	LOC110071649	60S ribosomal protein L7a OS=Pogona vitticeps OX=103695 GN=LOC110071649 PE=3 SV=1		2,598	0	<0,001	12	1	1	1	129	14,2	10,33
A0A6J0URP1	LOC110086649	Non-histone chromosomal protein HMG-14A OS=Pogona vitticeps OX=103695 GN=LOC110086649 PE=3 SV=1		2,571	0	<0,001	12	1	1	1	128	14,1	9,85
A0A6J0V2B5	PLG	Plasminogen OS=Pogona vitticeps OX=103695 GN=PLG PE=3 SV=1		2,55	0	<0,001	4	2	2	2	796	89,5	7,15
A0A6J0U2Z5	TNN	Tenascin-N OS=Pogona vitticeps OX=103695 GN=TNN PE=4 SV=1		2,511	0	<0,001	1	1	1	1	1033	115,3	5,91
A0A6J0SXQ0	CAT	Catalase OS=Pogona vitticeps OX=103695 GN=CAT PE=3 SV=1		2,51	0	<0,001	5	2	2	2	523	59,3	7,18
A0A6J0VE88	ARHGDIB	Rho GDP-dissociation inhibitor 2 OS=Pogona vitticeps OX=103695 GN=ARHGDIB PE=3 SV=1		2,507	0	<0,001	15	1	1	1	200	23,1	5,11
A0A6J0V8Q5	RPL7	Large ribosomal subunit protein uL30 OS=Pogona vitticeps OX=103695 GN=RPL7 PE=3 SV=1		2,504	0	<0,001	11	2	2	2	291	34	10,56
A0A6J0SBT4	LOC110070514	Class I histocompatibility antigen, F10 alpha chain-like OS=Pogona vitticeps OX=103695 GN=LOC110070514 PE=3 SV=1		2,499	2,1	<0,001	2	1	1	1	533	61,4	6,58
A0A6J0TRQ7	HSPD1	60 kDa heat shock protein, mitochondrial OS=Pogona vitticeps OX=103695 GN=HSPD1 PE=3 SV=1		2,489	0	<0,001	2	1	1	1	570	60,8	5,74
A0A6J0UJ41	HSD17B4	Peroxisomal multifunctional enzyme type 2 OS=Pogona vitticeps OX=103695 GN=HSD17B4 PE=3 SV=1		2,488	0	<0,001	2	1	1	1	728	79,4	8,7
A0A6J0V297	LOC110087987	Glutathione S-transferase OS=Pogona vitticeps OX=103695 GN=LOC110087987 PE=3 SV=1		2,435	2,23	<0,001	4	1	1	1	220	25,9	6,6
A0A6J0V9D9	LOC110090244	glutathione transferase OS=Pogona vitticeps OX=103695 GN=LOC110090244 PE=3 SV=1		2,415	0	<0,001	5	1	1	1	235	26,9	8,53
A0A6J0U8P7	PCOLCE	Procollagen C-endopeptidase enhancer 1 OS=Pogona vitticeps OX=103695 GN=PCOLCE PE=4 SV=1		2,394	1,77	<0,001	2	1	1	1	460	49,7	8,43
A0A6J0TT25	RPS10	40S ribosomal protein S10 OS=Pogona vitticeps OX=103695 GN=RPS10 PE=3 SV=1		2,321	0	<0,001	9	1	1	1	165	18,8	10,15
A0A6J0TPA7	FUBP1	Far upstream element-binding protein 1 isoform X1 OS=Pogona vitticeps OX=103695 GN=FUBP1 PE=4 SV=1		2,318	0	<0,001	3	2	2	2	682	72,2	7,36
A0A6J0SM82	KHDRBS2	KH domain-containing, RNA-binding, signal transduction-associated protein 2 OS=Pogona vitticeps OX=103695 GN=KHDRBS2 PE=3 SV=1		2,309	0	<0,001	3	1	2	1	348	39	5,95
A0A6J0VFA6	RPL23	Large ribosomal subunit protein uL14 OS=Pogona vitticeps OX=103695 GN=RPL23 PE=3 SV=1		2,298	0	<0,001	6	1	1	1	140	14,9	10,51
A0A6J0UF67	HEXA	Beta-hexosaminidase subunit alpha OS=Pogona vitticeps OX=103695 GN=HEXA PE=3 SV=1		2,293	0	<0,001	6	2	2	2	453	51,4	5,71
A0A6J0SU25	CTTN	Src substrate cortactin isoform X1 OS=Pogona vitticeps OX=103695 GN=CTTN PE=4 SV=1		2,289	1,77	<0,001	2	1	1	1	550	62	5,25
A0A6J0UIE8	RTN3	Reticulon OS=Pogona vitticeps OX=103695 GN=RTN3 PE=4 SV=1		2,256	0	<0,001	12	1	1	1	226	24,6	9,01
A0A6J0T685	NACA	Nascent polypeptide-associated complex subunit alpha isoform X4 OS=Pogona vitticeps OX=103695 GN=NACA PE=4 SV=1		2,251	0	<0,001	8	1	1	1	257	27,6	4,56
A0A6J0U9B3	YWHAH	14-3-3 protein eta isoform X1 OS=Pogona vitticeps OX=103695 GN=YWHAH PE=3 SV=1		2,235	0	<0,001	7	2	2	1	247	28,2	4,81
A0A6J0UKS1	YWHAG	14-3-3 protein gamma OS=Pogona vitticeps OX=103695 GN=YWHAG PE=3 SV=1		2,225	0	<0,001	8	2	2	1	247	28,3	4,89
A0A6J0SIG9	ECHS1	Enoyl-CoA hydratase, mitochondrial OS=Pogona vitticeps OX=103695 GN=ECHS1 PE=3 SV=1		2,195	0	<0,001	5	1	1	1	296	31,6	8,72
A0A6J0U119	GANAB	Neutral alpha-glucosidase AB OS=Pogona vitticeps OX=103695 GN=GANAB PE=3 SV=1		2,189	0	<0,001	2	1	1	1	968	108,6	5,62
A0A6J0U5L5	MAPK3	Mitogen-activated protein kinase OS=Pogona vitticeps OX=103695 GN=MAPK3 PE=3 SV=1		2,181	0	<0,001	6	1	1	1	376	42,6	6,61
A0A6J0TBU9	PLBD1	Phospholipase B-like OS=Pogona vitticeps OX=103695 GN=PLBD1 PE=3 SV=1		2,173	0	<0,001	3	1	1	1	556	64	7,62
A0A6J0U6A0	CTSK	Cathepsin K OS=Pogona vitticeps OX=103695 GN=CTSK PE=3 SV=1		2,162	0	<0,001	3	1	1	1	332	37,5	8,22
A0A6J0TG41	PTX3	Pentraxin-related protein PTX3 OS=Pogona vitticeps OX=103695 GN=PTX3 PE=4 SV=1		2,128	2,05	<0,001	2	1	1	1	432	48,5	5,06
A0A6J0SNX6	FBN3	Fibrillin-3 OS=Pogona vitticeps OX=103695 GN=FBN3 PE=3 SV=1		2,121	0	<0,001	0	1	1	1	2869	308,2	4,81
A0A6J0SSR7	ASCC3	Activating signal cointegrator 1 complex subunit 3 OS=Pogona vitticeps OX=103695 GN=ASCC3 PE=3 SV=1		2,119	0	<0,001	1	1	1	1	2204	251,4	6,77
A0A6J0V132	CLNS1A	Methylosome subunit pICln OS=Pogona vitticeps OX=103695 GN=CLNS1A PE=3 SV=1		2,091	0	<0,001	4	1	1	1	242	26,9	4,2
A0A6J0SCF1	PEBP1	Phosphatidylethanolamine-binding protein 1 OS=Pogona vitticeps OX=103695 GN=PEBP1 PE=3 SV=1		2,066	0	<0,001	7	1	1	1	187	21,1	6,2
A0A6J0UC61	ALDH2	Aldehyde dehydrogenase, mitochondrial OS=Pogona vitticeps OX=103695 GN=ALDH2 PE=3 SV=1		2,059	0	<0,001	2	1	1	1	422	46,4	6,84
A0A6J0T3H9	LOC110075876	Fatty acid-binding protein, adipocyte OS=Pogona vitticeps OX=103695 GN=LOC110075876 PE=3 SV=1		2,056	0	<0,001	8	1	1	1	132	14,9	6,57
A0A6J0SYB8	OPHN1	Oligophrenin-1 OS=Pogona vitticeps OX=103695 GN=OPHN1 PE=4 SV=1		2,047	1,96	<0,001	2	1	1	1	804	90,8	7,85
A0A6J0TXL1	PKLR	Pyruvate kinase OS=Pogona vitticeps OX=103695 GN=PKLR PE=3 SV=1		2,008	0	<0,001	2	1	1	1	573	63,2	8,7
A0A6J0SYH3	CD9	Tetraspanin OS=Pogona vitticeps OX=103695 GN=CD9 PE=3 SV=1		2	0	<0,001	7	1	1	1	225	24,7	7,46
A0A6J0V484	G6PD	Glucose-6-phosphate 1-dehydrogenase OS=Pogona vitticeps OX=103695 GN=G6PD PE=3 SV=1		1,98	0	<0,001	2	1	1	1	520	59,8	6,99
A0A6J0V628	LOC110089531	Uncharacterized protein LOC110089531 OS=Pogona vitticeps OX=103695 GN=LOC110089531 PE=3 SV=1		1,973	0	<0,001	2	1	1	1	468	52,5	5,06
A0A6J0STU0	TALDO1	Transaldolase OS=Pogona vitticeps OX=103695 GN=TALDO1 PE=3 SV=1		1,967	0	<0,001	3	1	1	1	337	37,5	7,56
A0A6J0V2S8	RPS27	40S ribosomal protein S27 OS=Pogona vitticeps OX=103695 GN=RPS27 PE=3 SV=1		1,962	2	<0,001	6	1	1	1	128	14,2	9,32
A0A6J0UPD1	LOC110085354	Septin-2 OS=Pogona vitticeps OX=103695 GN=LOC110085354 PE=3 SV=1		1,961	1,61	<0,001	3	1	1	1	349	40,2	6,67
A0A6J0T656	C1QBP	Complement component 1 Q subcomponent-binding protein, mitochondrial OS=Pogona vitticeps OX=103695 GN=C1QBP PE=3 SV=1		1,937	0	<0,001	6	1	1	1	267	30	4,7
A0A6J0TX11	PMVK	Phosphomevalonate kinase OS=Pogona vitticeps OX=103695 GN=PMVK PE=4 SV=1		1,882	0	<0,001	4	1	1	1	190	21,9	5,16
A0A6J0TAL1	VPS35	Vacuolar protein sorting-associated protein 35 OS=Pogona vitticeps OX=103695 GN=VPS35 PE=3 SV=1		1,875	0	<0,001	2	1	1	1	807	92,8	5,53
A0A6J0SFE2	HTATIP2	Oxidoreductase HTATIP2 OS=Pogona vitticeps OX=103695 GN=HTATIP2 PE=4 SV=1		1,868	0	<0,001	5	1	1	1	296	31,9	8,69
A0A6J0TN98	RPL10	Large ribosomal subunit protein uL16 OS=Pogona vitticeps OX=103695 GN=RPL10 PE=3 SV=1		1,86	0	<0,001	7	1	1	1	215	24,7	10,17
A0A6J0US51	RPL5	60S ribosomal protein L5 OS=Pogona vitticeps OX=103695 GN=RPL5 PE=3 SV=1		1,839	0	<0,001	5	1	1	1	297	34,3	9,73
A0A6J0VGU9	NDUFA13	NADH dehydrogenase [ubiquinone] 1 alpha subcomplex subunit 13 OS=Pogona vitticeps OX=103695 GN=NDUFA13 PE=3 SV=1		1,825	0	<0,001	8	1	1	1	151	17,5	9,32
A0A6J0U360	LOC110082191	Cytochrome b-c1 complex subunit 2, mitochondrial OS=Pogona vitticeps OX=103695 GN=LOC110082191 PE=4 SV=1		1,792	0	<0,001	3	1	1	1	457	49,2	8,46
A0A6J0UWW9	CTSA	Carboxypeptidase OS=Pogona vitticeps OX=103695 GN=CTSA PE=3 SV=1		1,79	0	<0,001	2	1	1	1	510	57,3	7,69
A0A6J0SY58	LAMA3	Laminin subunit alpha-3 OS=Pogona vitticeps OX=103695 GN=LAMA3 PE=4 SV=1		1,774	0	<0,001	0	1	1	1	3411	378,9	6,51
A0A6J0UYU1	PAPLN	Papilin isoform X1 OS=Pogona vitticeps OX=103695 GN=PAPLN PE=4 SV=1		1,712	1,68	<0,001	1	1	1	1	1352	149,3	7,3
A0A6J0V9M6	LOC110089894	Digestive cysteine proteinase 2-like OS=Pogona vitticeps OX=103695 GN=LOC110089894 PE=3 SV=1		1,698	0	<0,001	2	1	1	1	547	61,6	7,2
A0A6J0VAP6	ARHGDIA	Rho GDP-dissociation inhibitor 1 OS=Pogona vitticeps OX=103695 GN=ARHGDIA PE=3 SV=1		1,69	0	<0,001	16	1	1	1	204	23,3	5,11
A0A6J0VL29	LOC110091619	Uncharacterized protein LOC110091619 OS=Pogona vitticeps OX=103695 GN=LOC110091619 PE=4 SV=1		1,688	5,56	<0,001	15	1	1	1	219	25,7	8,47
A0A6J0T8N9	PABPC1L	Polyadenylate-binding protein OS=Pogona vitticeps OX=103695 GN=PABPC1L PE=3 SV=1		1,679	0	<0,001	1	1	1	1	630	70,3	9,33
A0A6J0UG40	PSMA1	Proteasome subunit alpha type-1 OS=Pogona vitticeps OX=103695 GN=PSMA1 PE=3 SV=1		1,657	0	<0,001	2	1	1	1	326	36,8	8,12
A0A6J0SZD2	RPL18A	60S ribosomal protein L18a OS=Pogona vitticeps OX=103695 GN=RPL18A PE=3 SV=1		1,652	1,61	<0,001	5	1	1	1	176	20,7	10,58
A0A6J0TJ35	CCT4	T-complex protein 1 subunit delta OS=Pogona vitticeps OX=103695 GN=CCT4 PE=3 SV=1		1,632	1,7	0,002	2	1	1	1	544	58,5	7,61
A0A6J0SKK2	YBX1	LOW QUALITY PROTEIN: nuclease-sensitive element-binding protein 1 OS=Pogona vitticeps OX=103695 GN=YBX1 PE=4 SV=1		1,627	0	0,001	9	1	1	1	343	39,6	9,64
A0A6J0UQI0	FUS	RNA-binding protein FUS OS=Pogona vitticeps OX=103695 GN=FUS PE=3 SV=1		1,627	0	0,001	4	1	1	1	506	51,7	9,55
A0A6J0T606	HNRNPU	Heterogeneous nuclear ribonucleoprotein U OS=Pogona vitticeps OX=103695 GN=HNRNPU PE=4 SV=1		1,624	0	0,001	2	1	1	1	815	89,3	7,55
A0A6J0SJK3	RPS28	Small ribosomal subunit protein eS28 OS=Pogona vitticeps OX=103695 GN=RPS28 PE=3 SV=1		1,604	0	0,001	22	1	1	1	69	7,8	10,7
A0A6J0S9Y6	RACK1	Small ribosomal subunit protein RACK1 OS=Pogona vitticeps OX=103695 GN=RACK1 PE=3 SV=1		1,596	0	0,001	3	1	1	1	317	35,1	7,69
A0A6J0VGG7	DPYSL2	Dihydropyrimidinase-related protein 2 OS=Pogona vitticeps OX=103695 GN=DPYSL2 PE=3 SV=1		1,568	0	0,001	2	1	1	1	687	74,7	6,46
A0A6J0UTH2	RPL15	Ribosomal protein L15 OS=Pogona vitticeps OX=103695 GN=RPL15 PE=3 SV=1		1,568	0	0,001	5	1	1	1	204	24,1	11,62
A0A6J0VAB1	EEF1G	Elongation factor 1-gamma OS=Pogona vitticeps OX=103695 GN=EEF1G PE=4 SV=1		1,539	0	0,001	2	1	1	1	432	49,7	6,87
A0A6J0UY22	MGST1	Microsomal glutathione S-transferase 1 OS=Pogona vitticeps OX=103695 GN=MGST1 PE=3 SV=1		1,537	0	0,001	6	1	1	1	156	17,9	9
A0A6J0VBR4	USP7	Ubiquitin carboxyl-terminal hydrolase 7 OS=Pogona vitticeps OX=103695 GN=USP7 PE=3 SV=1		1,528	0	0,001	1	1	1	1	1055	123	5,8
A0A6J0U6Q5	DIAPH1	LOW QUALITY PROTEIN: protein diaphanous homolog 1 OS=Pogona vitticeps OX=103695 GN=DIAPH1 PE=3 SV=1		1,51	0	0,001	1	1	1	1	1235	137,2	5,47
A0A6J0U7I2	RPL14	Large ribosomal subunit protein eL14 OS=Pogona vitticeps OX=103695 GN=RPL14 PE=3 SV=1		1,49	0	0,001	11	1	1	1	142	16,4	10,33
A0A6J0TRX7	C9	Complement component C9 OS=Pogona vitticeps OX=103695 GN=C9 PE=3 SV=1		1,486	0	0,003	2	1	1	1	597	66,1	6,19
A0A6J0UFW8	MDH1	Malate dehydrogenase OS=Pogona vitticeps OX=103695 GN=MDH1 PE=3 SV=1		1,479	0	0,003	4	1	1	1	334	36,4	7,71
A0A6J0TEK9	VCL	Vinculin OS=Pogona vitticeps OX=103695 GN=VCL PE=3 SV=1		1,478	0	0,003	1	1	2	1	1141	125,2	5,4
A0A6J0SIU8	CAPZA1	F-actin-capping protein subunit alpha OS=Pogona vitticeps OX=103695 GN=CAPZA1 PE=3 SV=1		1,47	0	0,003	5	1	1	1	286	32,9	5,82
A0A6J0SF53	EEF1D	Elongation factor 1-delta OS=Pogona vitticeps OX=103695 GN=EEF1D PE=3 SV=1		1,466	1,63	0,003	3	1	1	1	321	35,6	5,36
A0A6J0UZ35	LOC110088132	H(+)-transporting two-sector ATPase OS=Pogona vitticeps OX=103695 GN=LOC110088132 PE=3 SV=1		1,428	0	0,004	1	1	2	1	617	68,1	5,8
A0A6J0UPL0	TXNRD1	Thioredoxin reductase 1, cytoplasmic OS=Pogona vitticeps OX=103695 GN=TXNRD1 PE=3 SV=1		1,403	0	0,004	2	1	1	1	613	66,9	7,99
A0A6J0UEY8	CAPG	Macrophage-capping protein OS=Pogona vitticeps OX=103695 GN=CAPG PE=4 SV=1		1,398	0	0,004	6	1	1	1	349	39,2	6,95
A0A6J0UDR7	KIF20B	Kinesin-like protein KIF20B OS=Pogona vitticeps OX=103695 GN=KIF20B PE=3 SV=1		1,384	0	0,004	1	1	1	1	1833	209,6	5,76
A0A6J0V5Q3	LOC110089474	Vitellogenin-1-like OS=Pogona vitticeps OX=103695 GN=LOC110089474 PE=4 SV=1		1,382	0	0,004	2	1	1	1	1599	180,3	7,99
A0A6J0U8E6	LOC110082321	Zonadhesin-like OS=Pogona vitticeps OX=103695 GN=LOC110082321 PE=4 SV=1		1,374	0	0,004	2	1	1	1	2713	281,7	6,1
A0A6J0VDH0	RNF39	RING finger protein 39 OS=Pogona vitticeps OX=103695 GN=RNF39 PE=4 SV=1		1,371	0	0,004	8	1	1	1	132	14,7	9,32
A0A6J0V0U8	RPS5	Small ribosomal subunit protein uS7 OS=Pogona vitticeps OX=103695 GN=RPS5 PE=3 SV=1		1,371	0	0,004	5	1	1	1	203	22,8	9,72
A0A6J0SIN8	CSDE1	Cold shock domain-containing protein E1 isoform X5 OS=Pogona vitticeps OX=103695 GN=CSDE1 PE=4 SV=1		1,359	0	0,004	1	1	1	1	796	88,5	6,2
A0A6J0STI4	LOC110073453	Core histone macro-H2A OS=Pogona vitticeps OX=103695 GN=LOC110073453 PE=4 SV=1		1,337	0	0,004	2	1	1	1	372	39,7	9,79

Suppl. Table S10. Proteomic analysis of back skin of *P. vitticeps*

Accession (Uniprot)	Gene Symbol	Description	Note	Sum PEP Score	Score Sequest HT: Sequest HT	Exp. q-value: Combined	Coverage [%]	# Peptides	# PSMs	# Unique Peptides	# AAs	MW [kDa]	calc. pI
A0A6J0SXS2	LOC110074850	Keratin, type I cytoskeletal 15-like isoform X1 OS=Pogona vitticeps OX=103695 GN=LOC110074850 PE=3 SV=1	KRT15	169,1	104,6	0	54	22	38	20	513	54,1	5,34
A0A6J0T561	LOC110074871	Keratin, type I cytoskeletal 15-like isoform X2 OS=Pogona vitticeps OX=103695 GN=LOC110074871 PE=3 SV=1	KRT24L	109,8	70,9	0	48	21	33	19	491	52,3	5,35
A0A6J0SY29	LOC110074904	Keratin, type I cytoskeletal 24-like isoform X2 OS=Pogona vitticeps OX=103695 GN=LOC110074904 PE=3 SV=1	KRT24	54,7	22,8	0	30	14	17	12	486	52,4	5,14
A0A6J0SXS9	LOC110074853	Keratin, type I cytoskeletal 10-like OS=Pogona vitticeps OX=103695 GN=LOC110074853 PE=4 SV=1	KRT9L1	30,8	15,9	0	17	6	8	6	441	47,5	4,78
A0A6J0T2W2	LOC110074898	Keratin, type I cytoskeletal 14-like OS=Pogona vitticeps OX=103695 GN=LOC110074898 PE=3 SV=1	KRT14L1	21,1	10,7	0	17	8	8	7	449	49,5	5,55
A0A6J0SXW5	LOC110074873	Keratin, type I cytoskeletal 10-like OS=Pogona vitticeps OX=103695 GN=LOC110074873 PE=3 SV=1	HAS4/KRT9LC4	4,2	3,8	0	5	2	2	1	368	41,4	5,2
A0A6J0VBD4	LOC110090296	Keratin, type II cytoskeletal 5-like isoform X2 OS=Pogona vitticeps OX=103695 GN=LOC110090296 PE=3 SV=1	KRT5L2	151,0	93,0	0	59	37	43	34	608	63,3	7,12
A0A6J0VFV2	LOC110090297	Keratin, type II cytoskeletal cochleal-like OS=Pogona vitticeps OX=103695 GN=LOC110090297 PE=4 SV=1	KRT78L2	77,8	36,8	0	30	19	23	17	654	66,8	8,29
A0A6J0VFU1	LOC110090289	Keratin, type II cytoskeletal 5 OS=Pogona vitticeps OX=103695 GN=LOC110090289 PE=3 SV=1	KRT78L5	33,5	19,1	0	16	6	9	4	609	64	6,65
A0A6J0VBD1	LOC110090293	Keratin, type II cytoskeletal 7-like OS=Pogona vitticeps OX=103695 GN=LOC110090293 PE=3 SV=1	HBS2/KRT78LC2	13,6	8,0	0	5	3	3	2	779	80	7,21
A0A6J0SA54	LOC110070018	Keratin, type II cytoskeletal cochleal-like OS=Pogona vitticeps OX=103695 GN=LOC110070018 PE=3 SV=1	KRT5L4	11,2	2,3	0	9	5	5	1	417	46,9	5,48
A0A6J0VFV7	LOC110090299	Keratin, type II cytoskeletal 5 OS=Pogona vitticeps OX=103695 GN=LOC110090299 PE=3 SV=1	KRT78L4	8,2	3,9	0	6	4	4	1	562	59,2	8,59
A0A6J0TYQ4	DSP	Desmoplakin OS=Pogona vitticeps OX=103695 GN=DSP PE=4 SV=1		150,3	71,7	0	15	46	55	46	2879	332,1	6,77
A0A6J0SNS3	PKP1	Plakophilin-1 OS=Pogona vitticeps OX=103695 GN=PKP1 PE=3 SV=1		49,6	29,7	0	24	13	15	13	739	82,2	9,13
A0A6J0T2Q4	JUP	Junction plakoglobin OS=Pogona vitticeps OX=103695 GN=JUP PE=3 SV=1		38,6	13,4	0	20	11	12	11	748	82,2	6,19
A0A6J0UWW8	LOC110087251	Desmocollin-1-like isoform X1 OS=Pogona vitticeps OX=103695 GN=LOC110087251 PE=4 SV=1		24,7	13,7	0	6	5	7	5	904	100,3	5,34
A0A6J0TDZ5	LOC110077646	Desmoglein-1-beta-like OS=Pogona vitticeps OX=103695 GN=LOC110077646 PE=4 SV=1		12,4	4,4	0	5	3	3	3	1224	128,3	4,78
A0A6J0STP6	PKP3	Plakophilin-3 OS=Pogona vitticeps OX=103695 GN=PKP3 PE=3 SV=1		6,2	2,4	0	2	2	2	2	835	92,8	9,41
A0A6J0UKF9	TTN	Titin OS=Pogona vitticeps OX=103695 GN=TTN PE=3 SV=1		2967,7	1584,1	0	25	735	897	735	35430	3936,1	6,37
A0A6J0T3P6	LOC110075058	Myosin-1B-like OS=Pogona vitticeps OX=103695 GN=LOC110075058 PE=3 SV=1		1899,4	1267,5	0	71	218	403	16	1934	222,4	5,87
A0A6J0T6E4	LOC110075082	Myosin-1B OS=Pogona vitticeps OX=103695 GN=LOC110075082 PE=3 SV=1		1862,2	1223,8	0	70	213	393	20	1935	222,7	5,85
A0A6J0SZ37	LOC110075078	Myosin-1B-like OS=Pogona vitticeps OX=103695 GN=LOC110075078 PE=3 SV=1		1793,9	1168,5	0	76	216	396	25	1939	223,2	5,86
A0A6J0SYW9	LOC110075059	Myosin heavy chain, skeletal muscle-like OS=Pogona vitticeps OX=103695 GN=LOC110075059 PE=3 SV=1		1493,7	947,6	0	65	182	336	13	1771	203,8	6,65
A0A6J0SZ26	LOC110075079	Myosin-1B-like isoform X4 OS=Pogona vitticeps OX=103695 GN=LOC110075079 PE=3 SV=1		1162,3	761,3	0	47	133	252	2	1930	222,1	5,76
A0A6J0SWK3	MYH7B	Myosin-7B isoform X1 OS=Pogona vitticeps OX=103695 GN=MYH7B PE=3 SV=1		1081,0	695,5	0	69	162	235	112	1943	224,1	5,86
A0A6J0U0D1	NEB	Nebulin OS=Pogona vitticeps OX=103695 GN=NEB PE=4 SV=1		1000,4	518,4	0	48	259	301	259	7006	811,6	9,04
A0A6J0SZ25	LOC110075072	Myosin-3 OS=Pogona vitticeps OX=103695 GN=LOC110075072 PE=3 SV=1		835,8	489,4	0	55	129	201	61	1944	224	5,8
A0A6J0UB89	COL6A3	Collagen alpha-3(VI) chain isoform X2 OS=Pogona vitticeps OX=103695 GN=COL6A3 PE=3 SV=1		653,8	399,3	0	37	121	166	121	3156	342,4	6,47
A0A6J0SYP5	COL12A1	Collagen alpha-1(XII) chain OS=Pogona vitticeps OX=103695 GN=COL12A1 PE=4 SV=1		469,1	275,7	0	33	95	127	95	3106	337,6	5,48
A0A6J0T2Z2	LOC110075081	Myosin-4-like OS=Pogona vitticeps OX=103695 GN=LOC110075081 PE=3 SV=1		444,4	297,1	0	22	57	111	3	1938	223,3	5,82
A0A6J0T933	ACTC1	Actin, alpha cardiac muscle 1 OS=Pogona vitticeps OX=103695 GN=ACTC1 PE=3 SV=1		390,0	419,1	0	80	40	188	2	377	42	5,39
A0A6J0U140	ALDOA	Fructose-bisphosphate aldolase OS=Pogona vitticeps OX=103695 GN=ALDOA PE=3 SV=1		358,4	249,5	0	87	43	91	25	364	39,6	8,24
A0A6J0T6F6	CKM	Creatine kinase M-type OS=Pogona vitticeps OX=103695 GN=CKM PE=3 SV=1		324,1	227,3	0	83	47	106	47	381	43,1	7,09
A0A6J0V1J1	LOC110087836	Myosin-7 OS=Pogona vitticeps OX=103695 GN=LOC110087836 PE=3 SV=1		316,6	191,9	0	17	51	83	4	1911	220,3	6,14
A0A6J0V7Y0	LOC110089918	Alpha-fetoprotein-like OS=Pogona vitticeps OX=103695 GN=LOC110089918 PE=4 SV=1		304,4	184,3	0	65	44	77	31	613	69,7	6,15
A0A6J0SU20	LOC110073536	Actin, aortic smooth muscle-like isoform X1 OS=Pogona vitticeps OX=103695 GN=LOC110073536 PE=3 SV=1		301,8	324,1	0	60	28	159	1	377	42	5,31
A0A6J0V847	ACTN3	Alpha-actinin-3 OS=Pogona vitticeps OX=103695 GN=ACTN3 PE=3 SV=1		300,9	182,4	0	56	46	75	32	896	103,7	5,48
A0A6J0U4C2	ENO3	Beta-enolase OS=Pogona vitticeps OX=103695 GN=ENO3 PE=3 SV=1		299,7	198,8	0	81	32	65	25	342	37,4	6,8
A0A6J0TM70	COL1A1	Collagen alpha-1(I) chain OS=Pogona vitticeps OX=103695 GN=COL1A1 PE=4 SV=1		258,7	156,1	0	41	42	76	29	1453	137,9	5,87
A0A6J0T2W8	TPM1	Tropomyosin alpha-1 chain OS=Pogona vitticeps OX=103695 GN=TPM1 PE=3 SV=1		249,8	146,2	0	93	42	68	30	284	32,7	4,74
A0A6J0UMU4	MYH15	Myosin-15 isoform X1 OS=Pogona vitticeps OX=103695 GN=MYH15 PE=3 SV=1		244,1	150,0	0	15	39	58	4	1979	228,2	5,86
A0A6J0TJA2	ATP2A1	Calcium-transporting ATPase OS=Pogona vitticeps OX=103695 GN=ATP2A1 PE=3 SV=1		240,2	162,4	0	44	41	62	27	994	109,1	5,25
A0A6J0UNX2	GAPDH	Glyceraldehyde-3-phosphate dehydrogenase OS=Pogona vitticeps OX=103695 GN=GAPDH PE=3 SV=1		234,6	164,5	0	91	26	62	26	333	35,8	8,54
A0A6J0V5G5	FLNC	Filamin-C OS=Pogona vitticeps OX=103695 GN=FLNC PE=3 SV=1		223,8	133,2	0	28	48	59	44	2560	273,9	6,14
A0A6J0UFN0	LOC110084468	Actin, cytoplasmic 3-like isoform X1 OS=Pogona vitticeps OX=103695 GN=LOC110084468 PE=3 SV=1		219,6	262,6	0	38	22	137	1	376	41,9	5,58
A0A6J0UVA9	MYBPC2	Myosin-binding protein C, fast-type isoform X2 OS=Pogona vitticeps OX=103695 GN=MYBPC2 PE=4 SV=1		208,1	107,9	0	42	41	48	41	1142	128,7	6,83
A0A6J0UG01	PYGM	Alpha-1,4 glucan phosphorylase OS=Pogona vitticeps OX=103695 GN=PYGM PE=3 SV=1		206,0	99,9	0	53	40	53	40	843	97,1	7,23
A0A6J0TE35	PKM	Pyruvate kinase OS=Pogona vitticeps OX=103695 GN=PKM PE=3 SV=1		203,8	137,3	0	73	32	50	31	527	57,5	7,71
A0A6J0U829	MYBPC1	Myosin-binding protein C, slow-type isoform X3 OS=Pogona vitticeps OX=103695 GN=MYBPC1 PE=4 SV=1		188,7	111,0	0	34	41	53	41	1242	139	5,95
A0A6J0S9P1	DCN	Decorin OS=Pogona vitticeps OX=103695 GN=DCN PE=3 SV=1		183,1	111,7	0	70	34	52	34	357	39,5	8,69
A0A6J0VE48	AHNAK	LOW QUALITY PROTEIN: neuroblast differentiation-associated protein AHNAK OS=Pogona vitticeps OX=103695 GN=AHNAK PE=4 SV=1		181,9	70,7	0	18	40	52	40	5378	574,3	5,96
A0A6J0T3Z2	LOC110075272	Ovotransferrin-like OS=Pogona vitticeps OX=103695 GN=LOC110075272 PE=3 SV=1		167,0	92,9	0	58	29	36	29	702	76,1	6,87
A0A6J0VF06	COL6A2	Collagen alpha-2(VI) chain isoform X1 OS=Pogona vitticeps OX=103695 GN=COL6A2 PE=4 SV=1		161,9	91,7	0	22	25	35	25	1018	108,1	5,76
A0A6J0T0H5	COL1A2	Collagen alpha-2(I) chain OS=Pogona vitticeps OX=103695 GN=COL1A2 PE=4 SV=1		155,9	130,1	0	30	33	58	33	1363	129,2	8,94
A0A6J0UKQ9	TPI1	Triosephosphate isomerase OS=Pogona vitticeps OX=103695 GN=TPI1 PE=3 SV=1		153,3	103,2	0	94	26	36	26	248	26,7	7,15
A0A6J0VGY4	COL6A1	Collagen alpha-1(VI) chain OS=Pogona vitticeps OX=103695 GN=COL6A1 PE=4 SV=1		153,0	93,2	0	29	28	43	28	1017	108,5	5,74
A0A6J0U8P2	TPM2	Tropomyosin beta chain OS=Pogona vitticeps OX=103695 GN=TPM2 PE=3 SV=1		144,0	87,7	0	71	26	37	7	284	32,7	4,74
A0A6J0TRH1	MYOM1	Myomesin-1 OS=Pogona vitticeps OX=103695 GN=MYOM1 PE=4 SV=1		136,8	77,0	0	25	36	44	36	1639	184	6,16
A0A6J0SPQ0	MYOM2	Myomesin-2 OS=Pogona vitticeps OX=103695 GN=MYOM2 PE=4 SV=1		134,3	63,6	0	28	31	39	31	1454	164,1	5,8
A0A6J0SUG5	PGK1	Phosphoglycerate kinase OS=Pogona vitticeps OX=103695 GN=PGK1 PE=3 SV=1		134,2	74,1	0	58	23	31	23	417	44,5	7,71
A0A6J0VBV3	LOC110089939	Alpha-fetoprotein-like isoform X1 OS=Pogona vitticeps OX=103695 GN=LOC110089939 PE=4 SV=1		132,4	80,6	0	32	26	44	13	612	69,6	6,42
A0A6J0UGJ9	PGAM2	Phosphoglycerate mutase OS=Pogona vitticeps OX=103695 GN=PGAM2 PE=3 SV=1		126,4	59,5	0	47	15	24	9	254	28,9	8,48
A0A6J0T747	TNNI2	Troponin I, fast skeletal muscle OS=Pogona vitticeps OX=103695 GN=TNNI2 PE=3 SV=1		123,9	75,8	0	84	22	35	4	185	21,6	8,7
A0A6J0SSP7	ENO1	phosphopyruvate hydratase OS=Pogona vitticeps OX=103695 GN=ENO1 PE=3 SV=1		117,5	67,5	0	54	17	26	8	434	47,2	7,08
A0A6J0V2R3	TPM3	Tropomyosin alpha-3 chain isoform X4 OS=Pogona vitticeps OX=103695 GN=TPM3 PE=3 SV=1		115,6	63,4	0	62	21	30	1	284	32,8	4,72
A0A6J0T7E4	TNNT3	Troponin T, fast skeletal muscle isoform X22 OS=Pogona vitticeps OX=103695 GN=TNNT3 PE=3 SV=1		115,0	63,9	0	54	22	28	3	252	29,9	8,32
A0A6J0T7C3	TNNT3	Troponin T, fast skeletal muscle isoform X2 OS=Pogona vitticeps OX=103695 GN=TNNT3 PE=3 SV=1		114,1	68,6	0	40	22	28	3	266	31,6	6,61
A0A6J0T6C4	ACTN2	Alpha-actinin-2 OS=Pogona vitticeps OX=103695 GN=ACTN2 PE=3 SV=1		113,7	63,6	0	32	22	29	9	895	103,7	5,59
A0A6J0UJM2	LOC110084444	Actin, cytoplasmic 2-like OS=Pogona vitticeps OX=103695 GN=LOC110084444 PE=3 SV=1		109,6	92,3	0	46	19	49	2	332	37,3	5,55
A0A6J0V0N4	TPM3	Tropomyosin alpha-3 chain isoform X8 OS=Pogona vitticeps OX=103695 GN=TPM3 PE=3 SV=1		109,5	61,0	0	59	19	27	2	254	29,2	4,75
A0A6J0SJW9	TPM4	Tropomyosin alpha-4 chain isoform X1 OS=Pogona vitticeps OX=103695 GN=TPM4 PE=3 SV=1		108,1	60,8	0	51	19	28	5	284	32,8	4,73
A0A6J0U6Q6	TPM2	Tropomyosin beta chain isoform X6 OS=Pogona vitticeps OX=103695 GN=TPM2 PE=3 SV=1		108,0	66,1	0	61	18	28	1	248	28,6	4,73
A0A6J0ULM7	LDB3	LIM domain-binding protein 3 isoform X6 OS=Pogona vitticeps OX=103695 GN=LDB3 PE=4 SV=1		106,5	61,0	0	35	20	27	6	735	78,9	8,32
A0A6J0V0Z8	LOC110088549	L-lactate dehydrogenase OS=Pogona vitticeps OX=103695 GN=LOC110088549 PE=3 SV=1		104,8	60,1	0	58	20	29	16	332	36,6	7,88
A0A6J0UNM8	VIM	Vimentin OS=Pogona vitticeps OX=103695 GN=VIM PE=3 SV=1		103,7	76,8	0	58	29	41	26	462	53	5,17
A0A6J0THG0	DES	Desmin OS=Pogona vitticeps OX=103695 GN=DES PE=3 SV=1		102,6	59,3	0	65	26	30	24	462	53,2	5,27
A0A6J0T7B9	TNNI2	Troponin I, fast skeletal muscle OS=Pogona vitticeps OX=103695 GN=TNNI2 PE=3 SV=1		100,9	65,3	0	89	19	32	1	163	19	7,44
A0A6J0VCT5	LOC110089618	Heat shock cognate 71 kDa protein-like isoform X1 OS=Pogona vitticeps OX=103695 GN=LOC110089618 PE=3 SV=1		98,6	46,9	0	40	24	31	12	644	70,6	5,57
A0A6J0UPQ6	PLEC	Plectin isoform X9 OS=Pogona vitticeps OX=103695 GN=PLEC PE=4 SV=1		89,7	33,4	0	8	29	29	29	4586	523,8	6,1
A0A6J0T377	ANXA2	Annexin OS=Pogona vitticeps OX=103695 GN=ANXA2 PE=3 SV=1		89,4	58,1	0	67	19	23	19	339	38,6	8,05
A0A6J0VDX6	LOC110091302	Tubulin beta chain OS=Pogona vitticeps OX=103695 GN=LOC110091302 PE=3 SV=1		85,5	52,4	0	48	15	21	4	444	49,6	4,92
A0A6J0SJ14	LOC110071755	6-phosphofructokinase OS=Pogona vitticeps OX=103695 GN=LOC110071755 PE=3 SV=1		84,5	41,5	0	28	17	25	17	745	81,3	8,51
A0A6J0V0G0	EEF1A2	Elongation factor 1-alpha OS=Pogona vitticeps OX=103695 GN=EEF1A2 PE=3 SV=1		81,8	41,9	0	50	12	15	7	463	50,5	9,03
A0A6J0VBQ9	HSPB6	Heat shock protein beta-6 OS=Pogona vitticeps OX=103695 GN=HSPB6 PE=3 SV=1		81,0	41,6	0	84	13	21	13	171	19,3	6,96
A0A6J0UQU4	LDB3	LIM domain-binding protein 3 isoform X14 OS=Pogona vitticeps OX=103695 GN=LDB3 PE=4 SV=1		79,3	44,1	0	70	16	21	2	283	31,1	9,2
A0A6J0U5F9	GPI	Glucose-6-phosphate isomerase OS=Pogona vitticeps OX=103695 GN=GPI PE=3 SV=1		72,3	37,4	0	28	13	18	13	562	62,6	7,85
A0A6J0SRC1	MYL1	Myosin light chain 1/3, skeletal muscle isoform isoform X1 OS=Pogona vitticeps OX=103695 GN=MYL1 PE=4 SV=1		69,6	33,4	0	50	10	15	10	191	20,8	4,97
A0A6J0SV67	ATP5B	ATP synthase subunit beta OS=Pogona vitticeps OX=103695 GN=ATP5B PE=3 SV=1		68,0	48,5	0	28	11	20	11	531	56,6	5,41
A0A6J0VAZ2	LOC110090193	A.superbus venom factor 1-like OS=Pogona vitticeps OX=103695 GN=LOC110090193 PE=4 SV=1		67,7	46,9	0	16	18	22	15	1654	185,8	6,43
A0A6J0UCG2	ATP2A2	Calcium-transporting ATPase OS=Pogona vitticeps OX=103695 GN=ATP2A2 PE=3 SV=1		67,1	44,9	0	18	17	26	3	1042	114,9	5,31
A0A6J0T2U3	ACTN4	Alpha-actinin-4 OS=Pogona vitticeps OX=103695 GN=ACTN4 PE=3 SV=1		64,2	42,7	0	14	12	20	3	904	104,3	5,25
A0A6J0V5X6	FHL1	Four and a half LIM domains protein 1 isoform X3 OS=Pogona vitticeps OX=103695 GN=FHL1 PE=4 SV=1		63,9	43,4	0	52	12	15	2	297	33,9	8,41
A0A6J0URS5	PGM1	Phosphoglucomutase-1 OS=Pogona vitticeps OX=103695 GN=PGM1 PE=3 SV=1		63,2	29,4	0	28	12	16	12	562	61,6	7,01
A0A6J0TEF1	MYOZ1	Myozenin-1 isoform X1 OS=Pogona vitticeps OX=103695 GN=MYOZ1 PE=3 SV=1		62,3	40,7	0	58	14	15	14	308	33,3	8,84

Supplementary Table S11. Distances between genes at sites shown in figures 1, 2B and 3B

Locus	Species		Genomic scaffold (acc. nr.)	Genes between *KRT23* and *KRT15*	Distance (bp) *KRT23* - *KRT15*
Type I keratins	Worm lizard	*Rhineura floridana*	NC_084490.1	*ψ KRT36* (remnant)	79273
Type I keratins	Wall lizard	*Podarcis muralis*	NC_041324.1	*KRT36L1, KRT36L2*	49963
Type I keratins	Snake	*Thamnophis elegans*	NC_045558.1	none	50645
Type I keratins	Anole lizard	*Anolis carolinensis*	NC_085846.1	*KRT36L1, KRT36L2*	66185
**Locus**	**Species**		**Genomic scaffold (acc. nr.)**	**Genes between *KRT7* - *ETAA1***	**Distance (bp) *KRT7* - *ETAA1***
Type II keratins	Worm lizard	*Rhineura floridana*	NC_084482.1	*ψ KRT84L*'s (remnants)	42373
Type II keratins	Wall lizard	*Podarcis muralis*	NC_041313.1	*KRT84L1*, *KRT84L2*, *KRT84L3*, *KRT84L4*	152175
Type II keratins	Snake	*Thamnophis elegans*	NC_045542.1	none	25986
Type II keratins	Anole lizard	*Anolis carolinensis*	NC_085842.1	*KRT84L1*, *KRT84L2*, *KRT84L3*, *KRT84L4*	118646
**Locus**	**Species**		**Genomic scaffold (acc. nr.)**	**Genes between *LOR1* and *CBP1***	**Distance (bp) *LOR1* - *CBP1***
EDC	Worm lizard	*Rhineura floridana*	NC_084501.1	*EDPL*	40005
EDC	Wall lizard	*Podarcis muralis*	NC_041327.1	*EDPL*, *EDYM1*, 9 *EDCC*'s	144453
EDC	Snake	*Ophiophagus hannah*	AZIM01003248.1	none	17768
EDC	Anole lizard	*Anolis carolinensis*	NW_003338916.1	*EDYM1*, 3 *EDCC*s	70851

Abbreviations: bp, base pairs; acc. nr., accession number (GenBank)
